# Author Correction: ID3 regulates the MDC1-mediated DNA damage response in order to maintain genome stability

**DOI:** 10.1038/s41467-018-04599-6

**Published:** 2018-06-06

**Authors:** Jung-Hee Lee, Seon-Joo Park, Gurusamy Hariharasudhan, Min-Ji Kim, Sung Mi Jung, Seo-Yeon Jeong, In-Youb Chang, Cheolhee Kim, Eunae Kim, Jihyeon Yu, Sangsu Bae, Ho Jin You

**Affiliations:** 1 Laboratory of Genomic Instability and Cancer Therapeutics, Cancer Mutation Research Center, Chosun University School of Medicine, Gwangju, 501-759 Republic of Korea; 2 Department of Cellular and Molecular Medicine, Chosun University School of Medicine, Gwangju, 501-759 Republic of Korea; 3 Department of Premedical Sciences, Chosun University School of Medicine, Gwangju, 501-759 Republic of Korea; 4 Department of Anatomy, Chosun University School of Medicine, Gwangju, 501-759 Republic of Korea; 5 College of Pharmacy, Chosun University, 375 Seosuk-dong, Gwangju, 501-759 Republic of Korea; 6 Department of Chemistry, Hanyang University, Seoul, 04763 Republic of Korea; 7 Department of Pharmacology, Chosun University School of Medicine, Gwangju, 501-759 Republic of Korea

Correction to: *Nature Communications* 10.1038/s41467-017-01051-z, published online 12 October 2017

This Article contains errors in Figs. 3, 4 and 7, for which we apologize. In Fig. 3, panel ‘b’, the 0.5 h time point after Ku55933 treatment images were inadvertently replaced with duplicates of the 3 h time point after Ku55933 treatment images in Fig. 3b. Additionally, in panel ‘b’, the 0.5 h time point after Nu7026 treatment images were inadvertently replaced with duplicates of the 180 min time point after siMDC1 treatment images in Fig. 3d. The correct version of this figure appears below as Fig. 1. The raw data associated with this experiment are provided as a separate Supplementary Data 1 file.

**Fig. 1:**
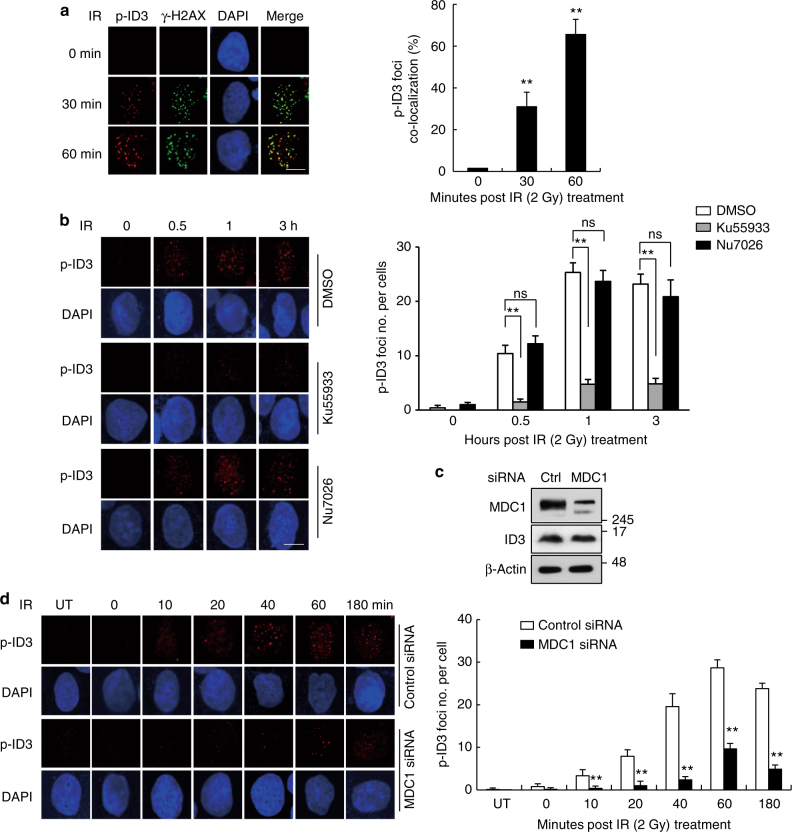
.

**Fig. 2:**
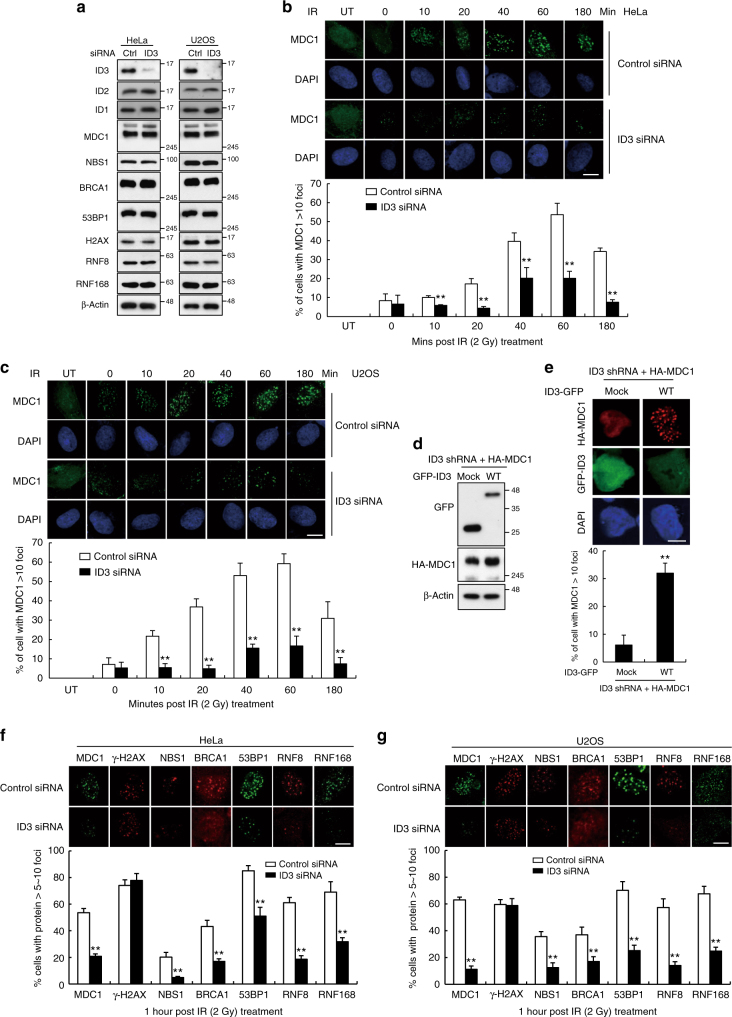
.

**Fig. 3:**
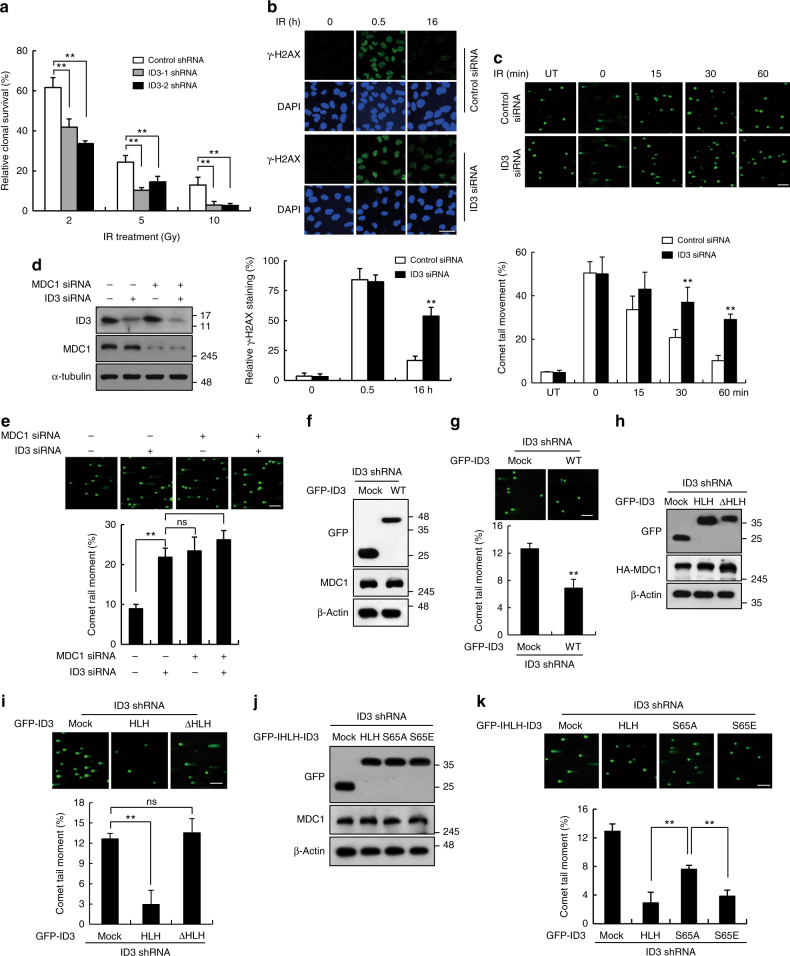
.

In Fig. 4, panel ‘g’, RNF168 foci in U2OS cell images were inadvertently replaced with duplicates of RNF168 foci in HeLa cell images in Fig. 4f. The correct version of this figure appears below as Fig. 2. The raw data associated with this experiment are provided as a separate Supplementary Data 2 file.

In Fig. 7, panel ‘b’, the DAPI images 0.5 h after IR under siID3 treatment were inadvertently replaced with DAPI images of a different field of view from the same experiment. Additionally, in panel ‘i’, the shID3 mock-treated GFP-ID3 cells image was inadvertently replace with duplications of the shID3 mock-treated GFP-ID3 cells image in Fig. 7g. The correct version of this figure appears below as Fig. 3. The raw data associated with this experiment are provided as a separate Supplementary Data 3 file.

## Electronic supplementary material

Supplementary Data 1

Supplementary Data 2

Supplementary Data 3

