# Supplementary material for: ID3 regulates the MDC1-mediated DNA damage response in order to maintain genome stability
Source: Nat Commun. 2017 Oct 12;8:903. doi: 10.1038/s41467-017-01051-z (PMC5638908; doi:10.1038/s41467-017-01051-z)
Supplement: Supplementary file 1 — Supplementary Information [file 41467_2017_1051_MOESM1_ESM.pdf]

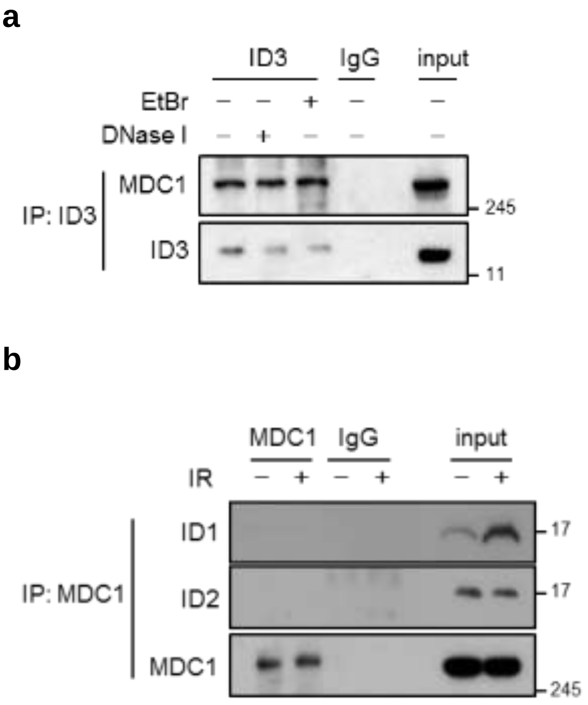

Supplementary Fig. 1 ID3 is specifically associated with MDC1. (**a**) The effect of ethidium bromide (EtBr) and DNase I on interactions between ID3 and MDC1 were measured by immunoprecipitation assay. HeLa cells were exposed to IR, and 3 h later, whole cell lysates were treated with 50 μg ml^{-1} EtBr on ice for 30 min, 100 μg ml^{-1} DNase I at 37 °C for 20 min, or were mock-treated. Cell lysates were then immunoprecipitated using the anti-ID3 antibody and detected by Western blotting using the antibodies indicated to the right of the blot. (**b**) HeLa cells were treated with and without exposure to IR. Three hours later, whole cell lysates were subjected to immunoprecipitation using anti-MDC1 and detected by Western blotting using the antibodies indicated to the right of the blot. Uncropped blots of this Figure accompanied by the location of molecular weight markers are shown in Supplementary Fig. 15.

a

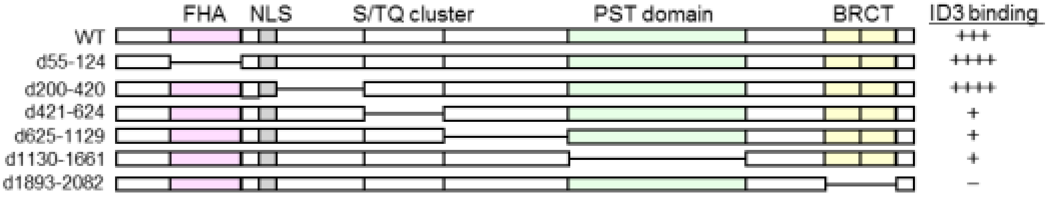

b

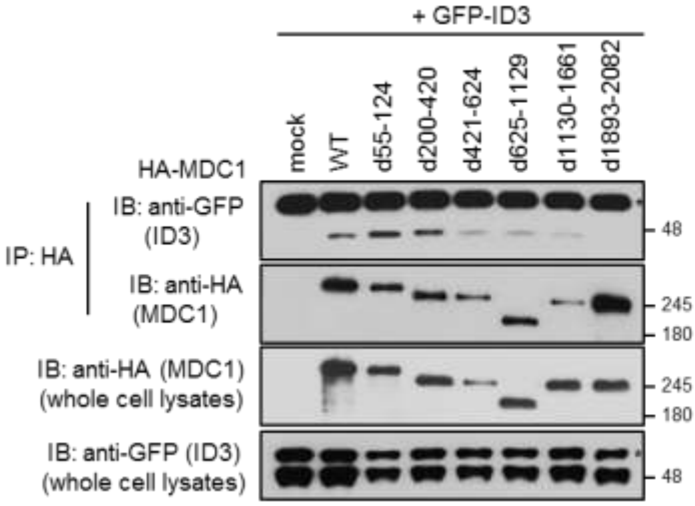

Supplementary Fig. 2 The BRCT domain (1892-2082 amino acid residues) of MDC1 is required for interactions with ID3. (**a**) A schematic representation of HA-tagged full-length MDC1 (WT) and the various deletion mutations is shown. Relative affinity of ID3 binding by each of the MDC1 deletion mutants is indicated on the right based on the *in vivo* binding assays presented in (**b**). (**b**) HEK293T cells were co-transfected with GFP-tagged ID3 and either HA-tagged wild type (WT) or deletion mutants of MDC1, followed by exposure to IR. Cell lysates were immunoprecipitated (IP) using anti-HA antibody and analyzed by immunoblotting (IB) using the indicated antibodies. Uncropped blots of this Figure accompanied by the location of molecular weight markers are shown in Supplementary Fig. 15.

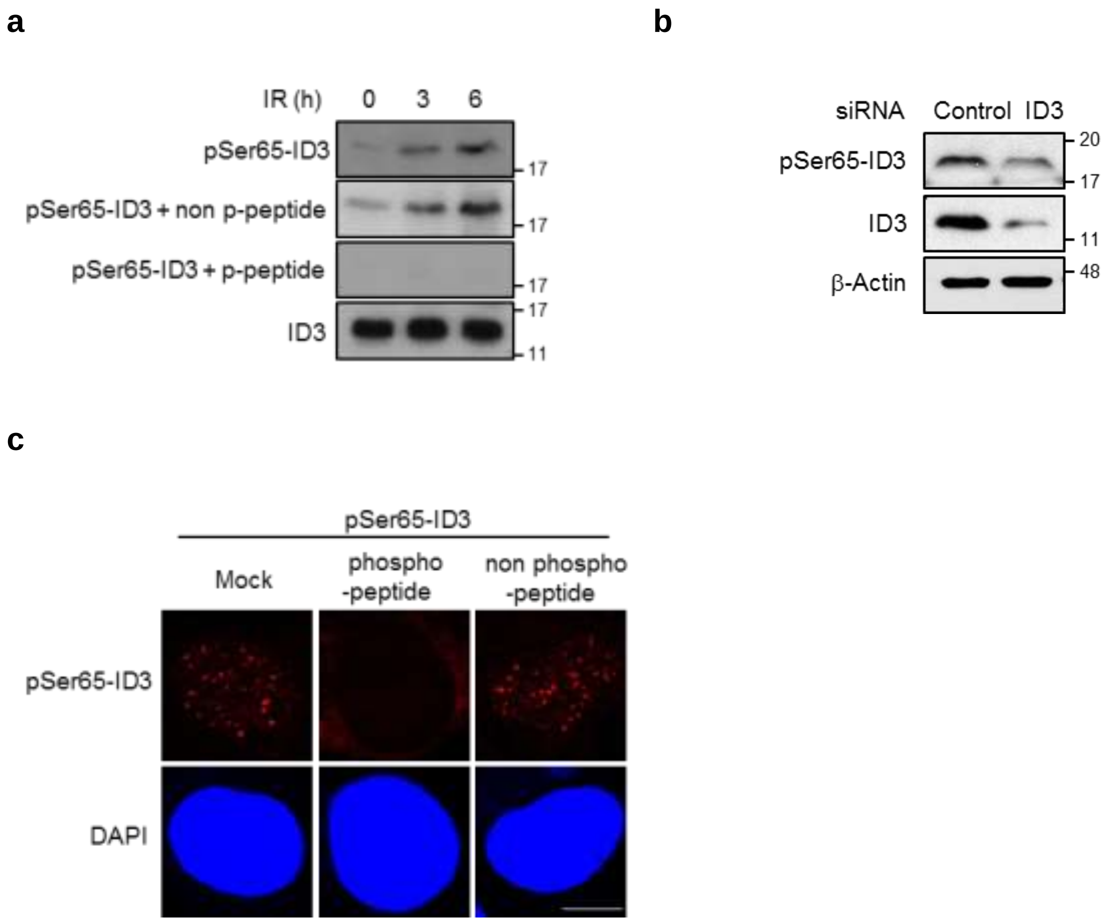

Supplementary Fig. 3 Endogenous ID3 phosphorylated at Ser65 in response to IR is specifically recognized by antibody to pSer65-ID3. (**a**) IR-induced phosphorylation of endogenous ID3 occurs at amino acid 65 in HeLa cells. HeLa cells with or without exposure to IR were collected at the indicated times following treatment. Western blotting analysis was performed using antibody against pSer65-ID3 in the presence of a control non-phosphorylated peptide or a phosphorylated peptide. (**b**) HeLa cells were transfected with either control siRNA or ID3-specific siRNA, and were then exposed to IR. At 24 h after treatment, whole cell lysates were analyzed by Western blotting using anti-pSer65-ID3 and anti-ID3 antibodies. α-tubulin was included as an internal control. (**c**) Endogenous ID3 localized to intracellular foci is specifically recognized by antibody to pSer65-ID3. HeLa cells were treated with IR. At 3 h after treatment, cells were fixed and immunofluorescent staining was carried out using antibody to pSer65-ID3 in the presence of a control non-phosphorylated peptide or a phosphorylated peptide. Nuclei were stained with DAPI. Scale bars: 10 μm. Uncropped blots of this Figure accompanied by the location of molecular weight markers are shown in Supplementary Fig. 15.

a

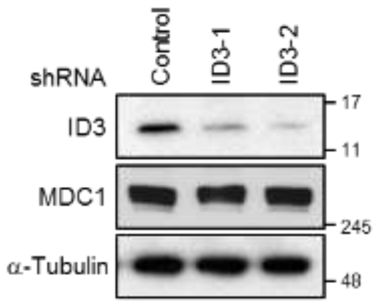

shRNA
Control
ID3-1
ID3-2
ID3
MDC1
α-Tubulin
17
11
245
48

b

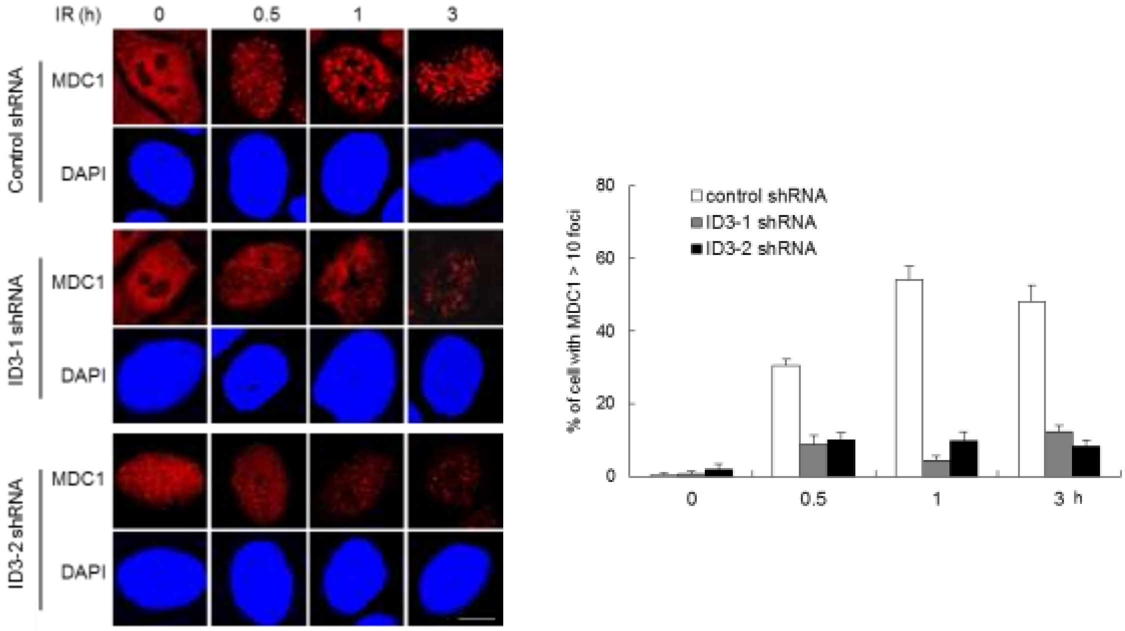

IR (h)
0
0.5
1
3
Control shRNA
ID3-1 shRNA
ID3-2 shRNA
MDC1
DAPI
% of cell with MDC1 > 10 foci
control shRNA
ID3-1 shRNA
ID3-2 shRNA
80
60
40
20
0
h

c

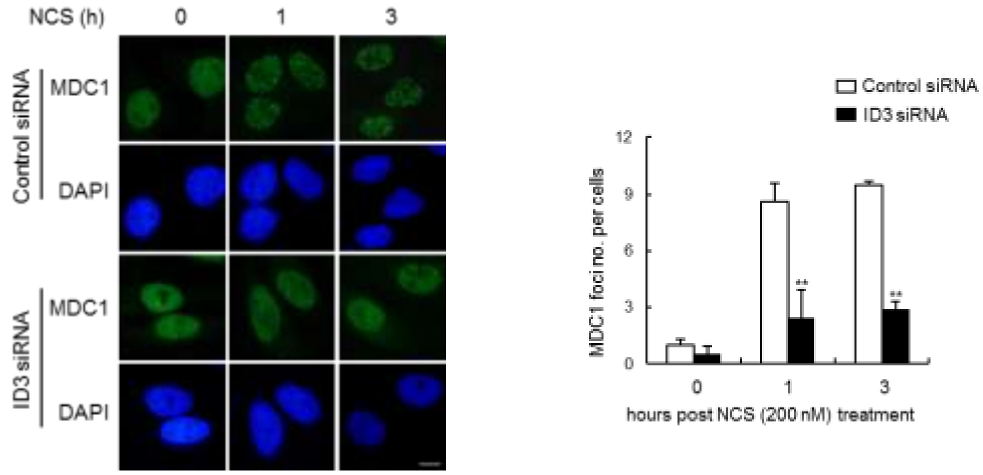

NCS (h)
0
1
3
Control siRNA
ID3 siRNA
MDC1
DAPI
MDC1 foci no. per cells
12
9
6
**
hours post NCS (200 nM) treatment

Supplementary Fig. 4 IR-induction of MDC1 foci is significantly reduced in HeLa cells with a stable ID3 knockdown. (**a**) Two shRNAs (ID3-1 and ID3-2), each designed against a different region of the *ID3* target gene, were stably transfected into HeLa cells. Western blotting was used to detect expression levels of ID3 and MDC1 in either control or stably ID3-depleted HeLa cells. (**b**) A time course of the formation of MDC1 foci per cell at the indicated times following exposure to IR is shown. Immunofluorescent staining was performed using an anti-MDC1 antibody. Nuclei were stained with DAPI. Representative images (left panel) and quantification (right panel) of the prevalence of MDC1 foci in a control and two different stable ID3 knockdown HeLa cell lines. At least 100 cells were counted at each time point (n = 3). Scale bars: 10 μm. (**c**) Neocarzinostatin (NCS)-induced MDC1 foci formation is impaired in ID3-depleted HeLa cells. HeLa cells transfected with either control siRNA or ID3 siRNA were treated with and without NCS (200 ng ml^{-1}) and fixed at the indicated time points. Immunofluorescent staining was performed using an anti-MDC1 antibody. Nuclei were stained with DAPI. Representative images (left panel) and quantification (right panel) of the number of MDC1 foci in control and ID3 knockdown cells (n = 3). Data are presented as means ± s.d. *P* values are based on Student's two-tailed *t*-test: ** $P < 0.01$. Scale bars: 10 μm. Uncropped blots of this Figure accompanied by the location of molecular weight markers are shown in Supplementary Fig. 15.

a

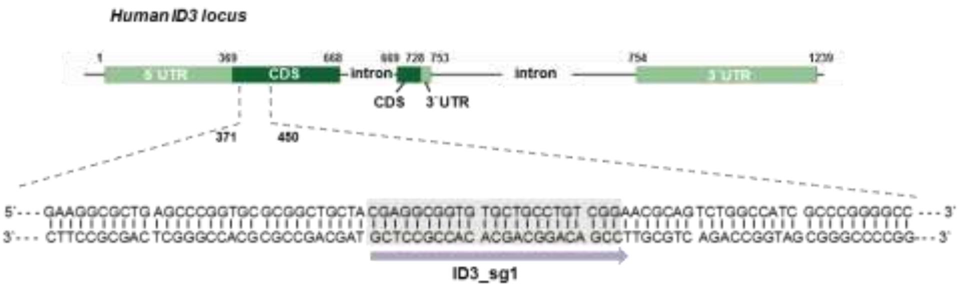

	RGEN Target (5' to 3')	Position	Direction	GC Contents (%, w/o PAM)	Out-of-frame Score	Mismatches		
						0	1	2
ID3_sg1	CGAGGCGGTGTGCTGCCTGTCGG	401	+	70	61.7	1	0	0
ID3_sg2	AACGCAGTCTGGCCATCGCCCGG	424	+	65	61.8	1	0	0
ID3_sg3	TCAGCGGCTCCTCAGCTGCCGGG	461	-	70	67.9	1	0	0
ID3_sg4	ATGTCGTCCAGCAAGCTCAGCGG	477	-	55	64.4	1	0	0
ID3_sg5	CGCCTGCGGGAACTGGTACCCGG	516	+	70	76.6	1	0	0

b

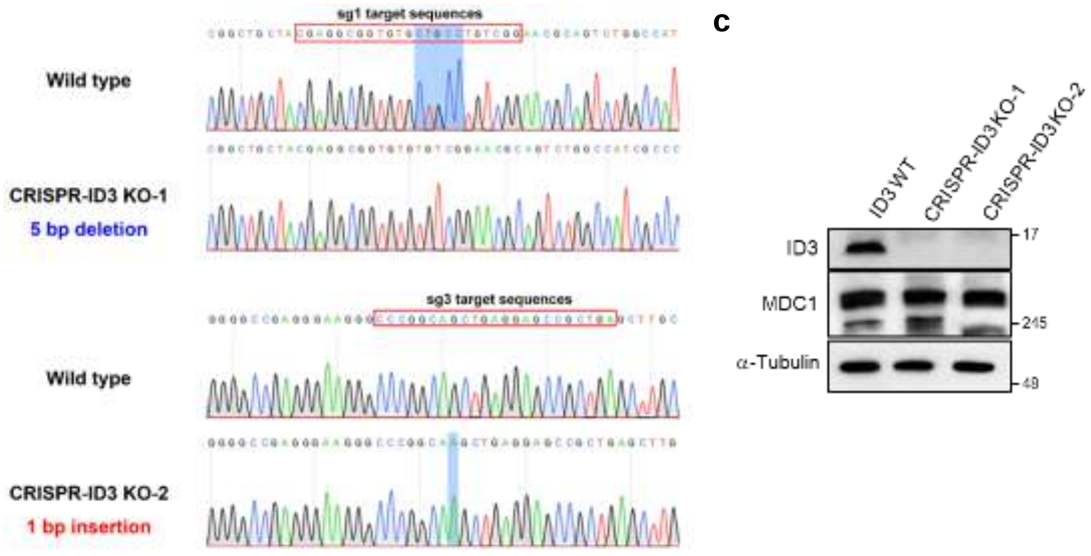

Supplementary Fig. 5 Generation of *ID3* KO cell lines using CRISPR-Cas9. (**a**) A diagram of the strategy for knocking out *ID3* using the CRISPR-Cas9 (upper panel) and the CRISPR-Cas9 target sequences for *ID3* gene knock-out (KO) (lower panel). We designed five sgRNAs (ID3_sg1-sg5) to target ID3 exon 1 and chose one ID3 sgRNA (ID3_sg1) due to high levels of transfection. Target sequences are highlighted in gray. Targets which had no potential off-target bearing 1-2 mismatches compared to the on-target were chosen. Cas9-Designer (http://www.rgenome.net/cas-designer/) was used for CRISPR target selection. (**b**) Sanger sequencing results of each *ID3* KO U2OS cell strain. CRISPR-ID3 KO-1 had 5-bp deletion and CRISPR-ID3 KO-2 had 1-bp insertion as a homogeneous mutation. Both mutations caused a codon frame-shift that led to premature stop codons within *ID3* coding sequences. (**c**) CRISPR/Cas9-mediated inactivation of *ID3* gene in two different KO clones of U2OS cells. Lack of ID3 protein expression was documented by Western blotting analysis. MDC1 and α-tubulin was included as an internal control. Uncropped blots of this Figure accompanied by the location of molecular weight markers are shown in Supplementary Fig. 15.

a

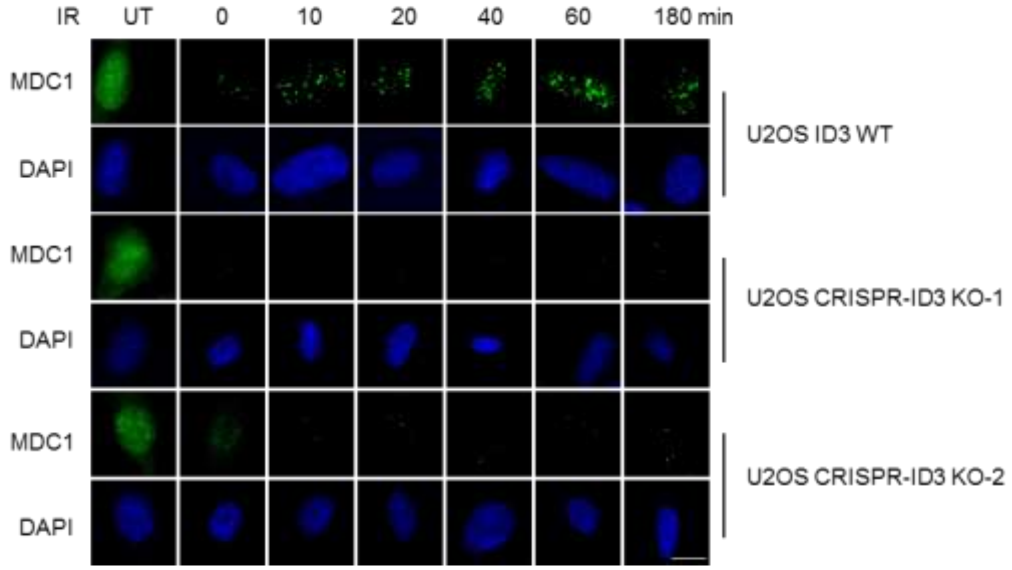
IR
UT
0
10
20
40
60
180 min
MDC1
DAPI
MDC1
DAPI
MDC1
DAPI
U2OS ID3 WT
U2OS CRISPR-ID3 KO-1
U2OS CRISPR-ID3 KO-2

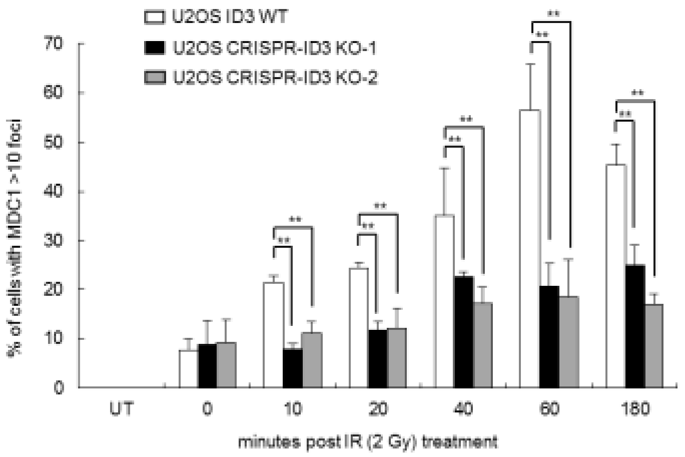
U2OS ID3 WT
U2OS CRISPR-ID3 KO-1
U2OS CRISPR-ID3 KO-2
% of cells with MDC1 >10 foci
70
60
50
40
30
20
10
0
UT
0
10
20
40
60
180
minutes post IR (2 Gy) treatment
**

b

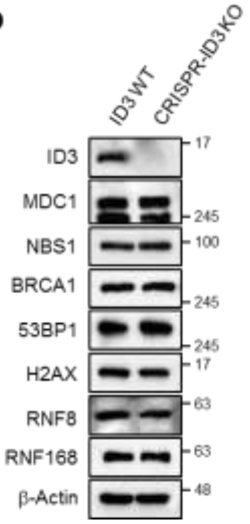
ID3 WT
CRISPR-ID3 KO
ID3
MDC1
NBS1
BRCA1
53BP1
H2AX
RNF8
RNF168
β-Actin
17
245
100
245
245
17
63
63
48

c

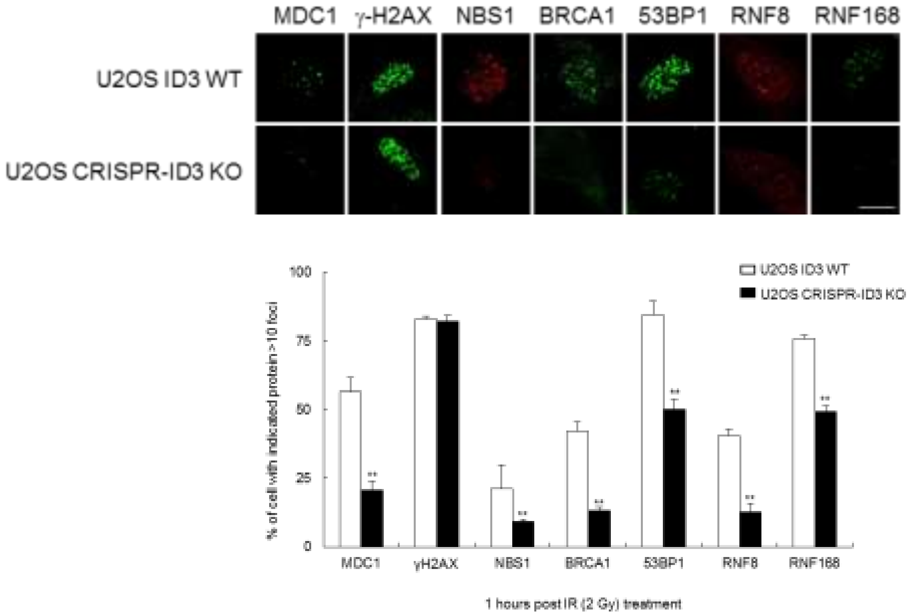
MDC1
γ-H2AX
NBS1
BRCA1
53BP1
RNF8
RNF168
U2OS ID3 WT
U2OS CRISPR-ID3 KO
% of cell with indicated protein >10 foci
100
75
50
25
0
U2OS ID3 WT
U2OS CRISPR-ID3 KO
MDC1
γH2AX
NBS1
BRCA1
53BP1
RNF8
RNF168
1 hours post IR (2 Gy) treatment
**

d

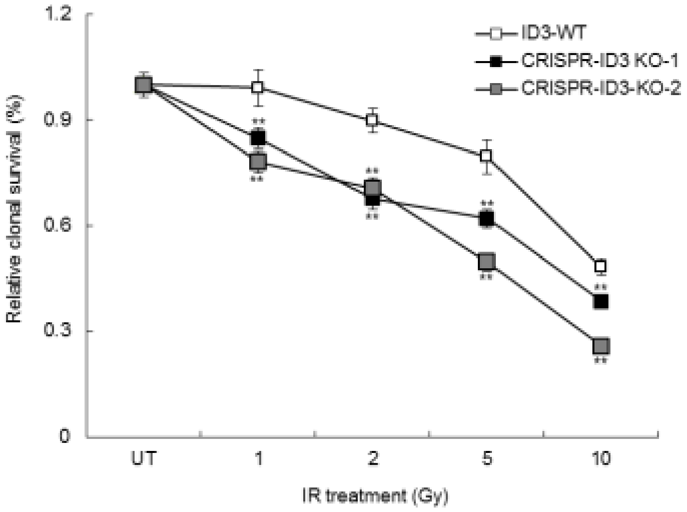

Supplementary Fig. 6 ID3 knockout U2OS cells displayed impaired MDC1 foci formation and increased sensitivity to IR. (**a**) *ID3* wild type (WT) U2OS (ID3-WT) and two different *ID3* KO U2OS (U2OS CRISPR-ID3 KO-1 and U2OS CRISPR-ID3 KO-2) cells were exposed to 2 Gy of IR and fixed at the indicated time points. Immunofluorescent staining was performed using an anti-MDC1 antibody. Nuclei were stained with DAPI. Representative images (upper panel) and quantification (lower panel) of the number of MDC1 foci in WT and *ID3* KO cells (n = 3). Scale bars: 10 μm. (**b**) Western blotting using antibodies to the indicated proteins shows the expression levels of each in *ID3* WT and KO U2OS cells. Uncropped blots of this Figure accompanied by the location of molecular weight markers are shown in Supplementary Fig. 15. (**c**) *ID3* WT and KO U2OS cells were exposed to 2 Gy of IR. Images depict representative nuclei showing MDC1, γ-H2AX, NBS1, BRCA1, 53BP1, RNF8 and RNF168 foci at 1 h after IR treatment. The lower panel shows the number of IR-induced foci in WT and *ID3* KO cells (n = 3). Scale bars: 10 μm. (**d**) *ID3* WT and KO U2OS cells were exposed to the indicated doses of IR and assessed for colony forming ability. The cell viability of untreated cells is defined as 100%. The experiment was carried out in triplicate. Data are presented as means ± s.d. P values are based on Student's two-tailed t-test: ** $P < 0.01$

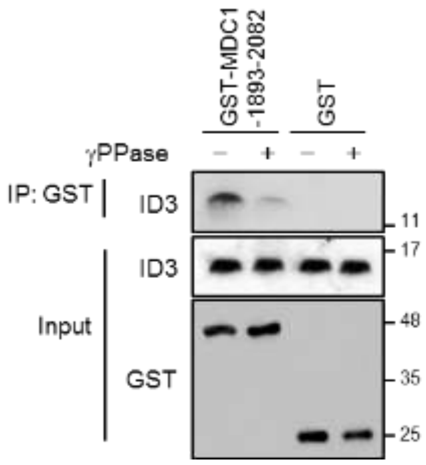

Supplementary Fig. 7 Phosphorylation-Dependent binding of ID3 to MDC1. Total cell lysates of HeLa cells that had been exposed to IR were incubated with either a GST-tagged fragment of MDC1 or a GST bead alone, in the presence or absence of γ protein phosphatase (γPPase). Immunoprecipitation was carried out using anti-GST antibody and analyzed by Western blotting using antibodies indicated to the right of the blot. Uncropped blots of this Figure accompanied by the location of molecular weight markers are shown in Supplementary Fig. 15.

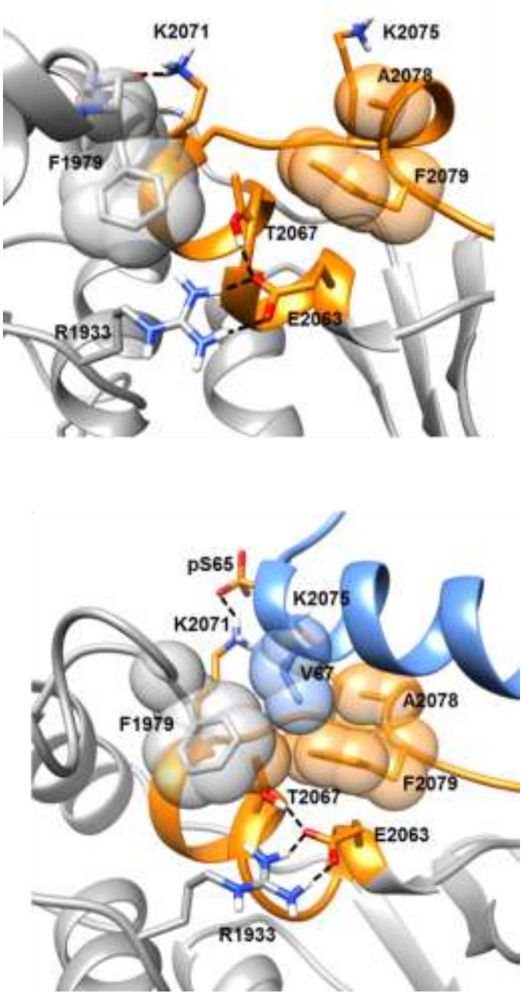

Supplementary Fig. 8 Amino acid residues of the MDC1-tBRCT domain predicted to play an important role in the formation of a complex with ID3. (**a**) A depiction of the most likely conformation of the MDC1 tBRCT domain as determined by normal MD simulations is shown. The grey ribbon represents the tBRCT domain with the ID3 binding region (residues 2063-2089) highlighted in orange. Side chains are included for relevant amino acids, such as Lys2071 (K2071) and Lys2075 (K2075), which play an important role in the recognition of phosphorylated ID3. A salt bridge network is formed by Arg1933 (R1933), Thr2067 (T2067) and Glu2063 (E2063), and sites of hydrogen bonding are indicated with a black dashed line. Phe1979 (F1979), Ala2078 (A2078) and Phe2079 (F2079) form a hydrophobic pocket. (**b**) Three dimensional structures from a docking model of the MDC1-tBRCT /ID3-pHLH complex are shown. The blue ribbon represents phosphorylated ID3-HLH (ID3-pHLH). The grey ribbon represents the tBRCT domain, with the ID3 binding region highlighted in orange. The side chains of Val67 (V67) from ID3 and Phe1979 (F1979), Ala2078 (A2078) and Phe2079 (F2079) from the tBRCT domain are shown as space filling models to demonstrate the hydrophobic core of the complex. The black dashed lines indicate hydrogen bonds.

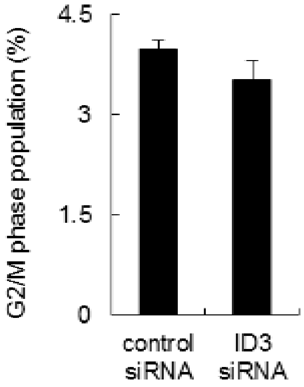

Supplementary Fig. 9 Effect of ID3 downregulation on IR-induced G2/M checkpoint. HeLa cells were transiently transfected with control or ID3 siRNA. 48 h after transfection, cells were treated with IR for 3 h and then incubated in 100 ng ml^{-1} nocodazole-containing media for 3 h before fixation. Cells in mitosis were determined by staining with propidium iodide and phosphohistone H3-Alexa Fluor 647-conjugated antibody and analyzed by flow cytometry. The percentages of phospho-histone H3 positive cells from three independent experiments were measured. Data are presented as means ± s.d. *P* values are based on Student's two-tailed *t*-test.

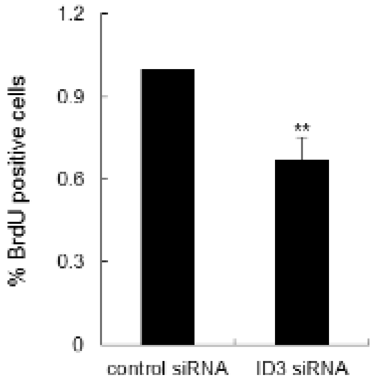

Supplementary Fig. 10 Effect of ID3 downregulation on S-phase entry. HeLa cells were transfected with either control siRNA or ID3 siRNA, and were treated with BrdU. DNA synthesis was assessed based on BrdU incorporation. Data are expressed as relative BrdU incorporation of the untreated control cells (n = 3). Data are presented as means ± s.d. *P* values are based on Student's two-tailed *t*-test: ** $P < 0.01$

a

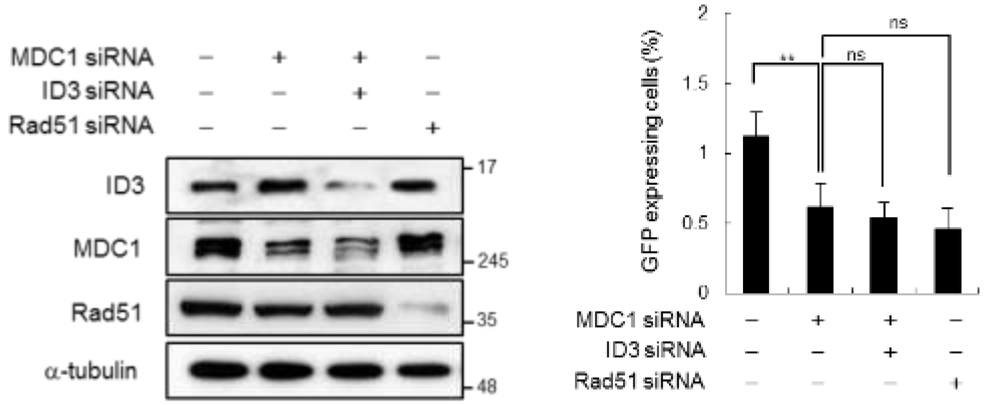

b

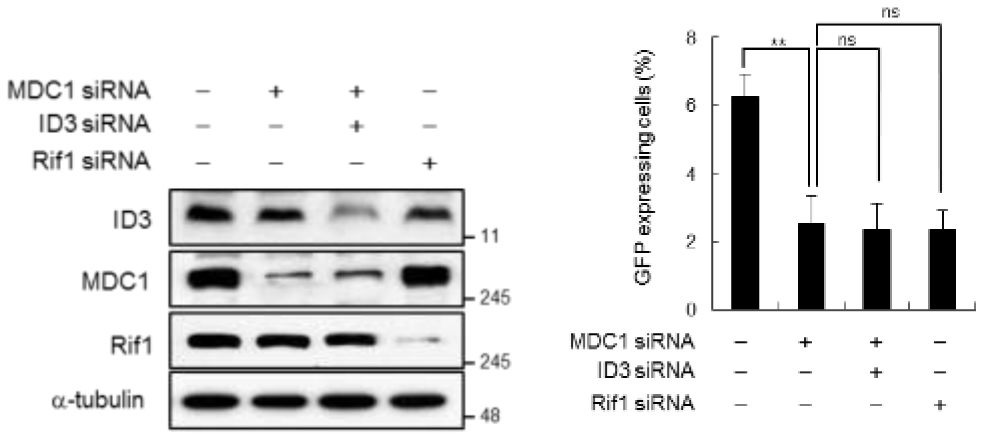

Supplementary Fig. 11 ID3 promotes HR and NHEJ. (**a**) A Western blot (left panel) of ID3 and MDC1 expression levels in the indicated siRNA-transfected DR-GFP U2OS cells is shown. HR repair efficiency (right panel) was measured in DR-GFP-U2OS cells transfected with indicated siRNAs (n = 3). α-tubulin was included as an internal control. **(b**) A Western blot (left panel) of ID3 and MDC1 proteins in the indicated siRNA-transfected EJ5-GFP HeLa cells is shown. NHEJ repair efficiency (right panel) was measured in EJ5-GFP HeLa cells transfected with indicated siRNAs (n = 3). α-tubulin was included as an internal control. Data are presented as means ± s.d. *P* values are based on Student's two-tailed *t*-test: ns, not significant, ** *P* < 0.01. Uncropped blots of this Figure accompanied by the location of molecular weight markers are shown in Supplementary Fig. 15.

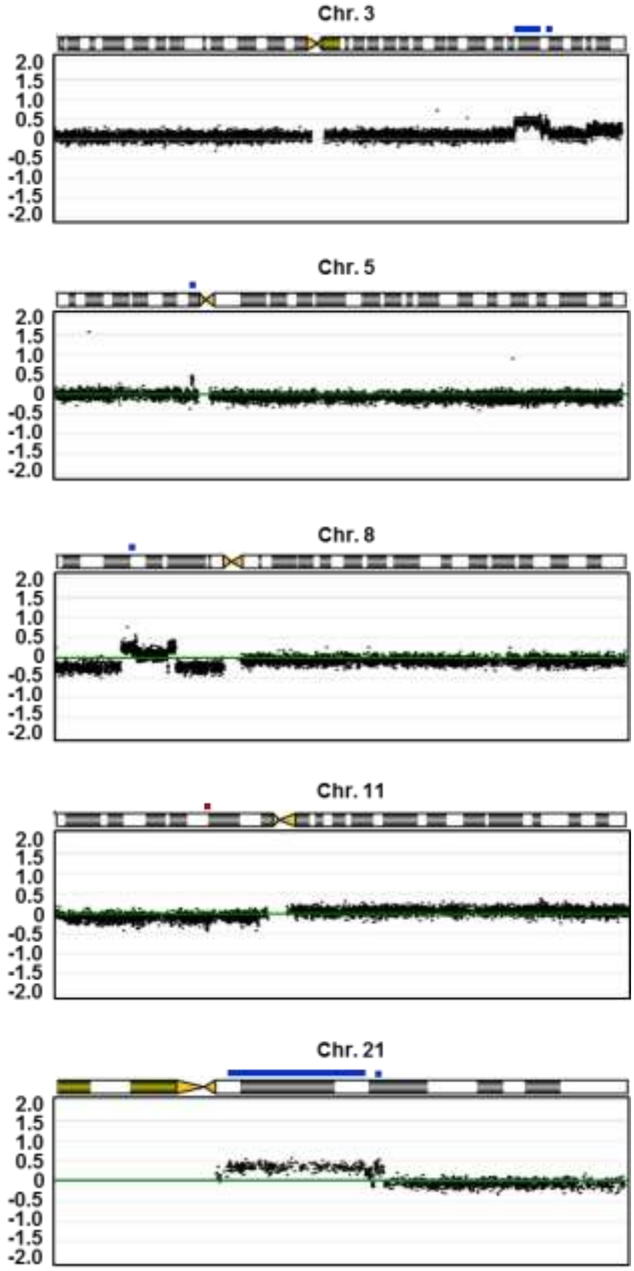

Supplementary Figure 12. Genome-Wide DNA Copy Number Variation in ID3-Depleted GM00637 Cells. Using the copy number of control siRNA-transfected MRC-5 cells as a baseline, the relative changes in copy number for ID3-specific siRNA-transfected MRC-5 cells were measured. Chromosomal regions above or below the red dotted line indicate amplifications or deletions of genomic positions, respectively. Schematic structures of chromosomes are displayed with their corresponding chromosome numbers. Array comparative genomic hybridization detected alterations of the clonal DNA copy number, including amplification in Chr3, Chr5, Chr8, and Chr21 and deletion in Chr11 in ID3-depleted GM00637 cells.

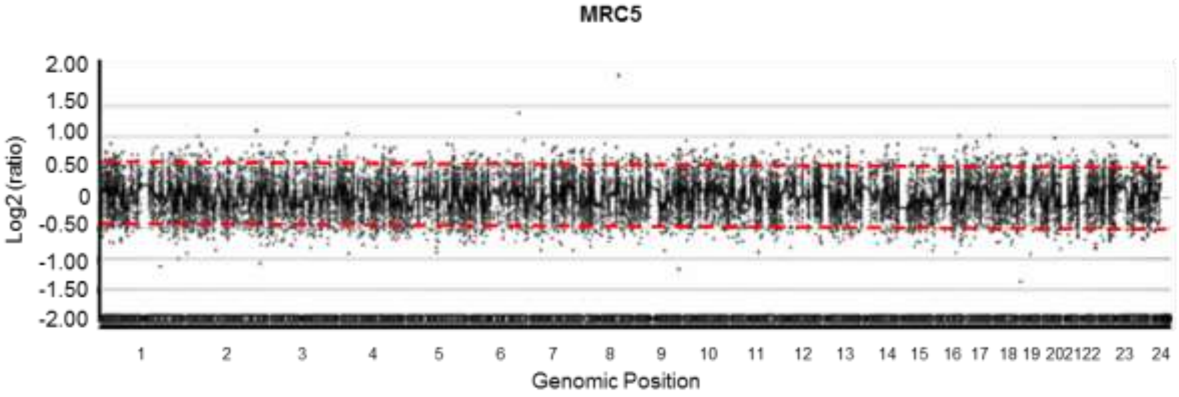

Supplementary Figure 13. Genome-wide DNA copy number variation in ID3-depleted MRC-5 cells. Using the copy number of control siRNA-transfected MRC-5 cells as a baseline, the relative changes in copy number for ID3-specific siRNA-transfected MRC-5 cells were measured. Chromosomal regions above or below the red dotted line indicate amplifications or deletions of genomic positions, respectively.

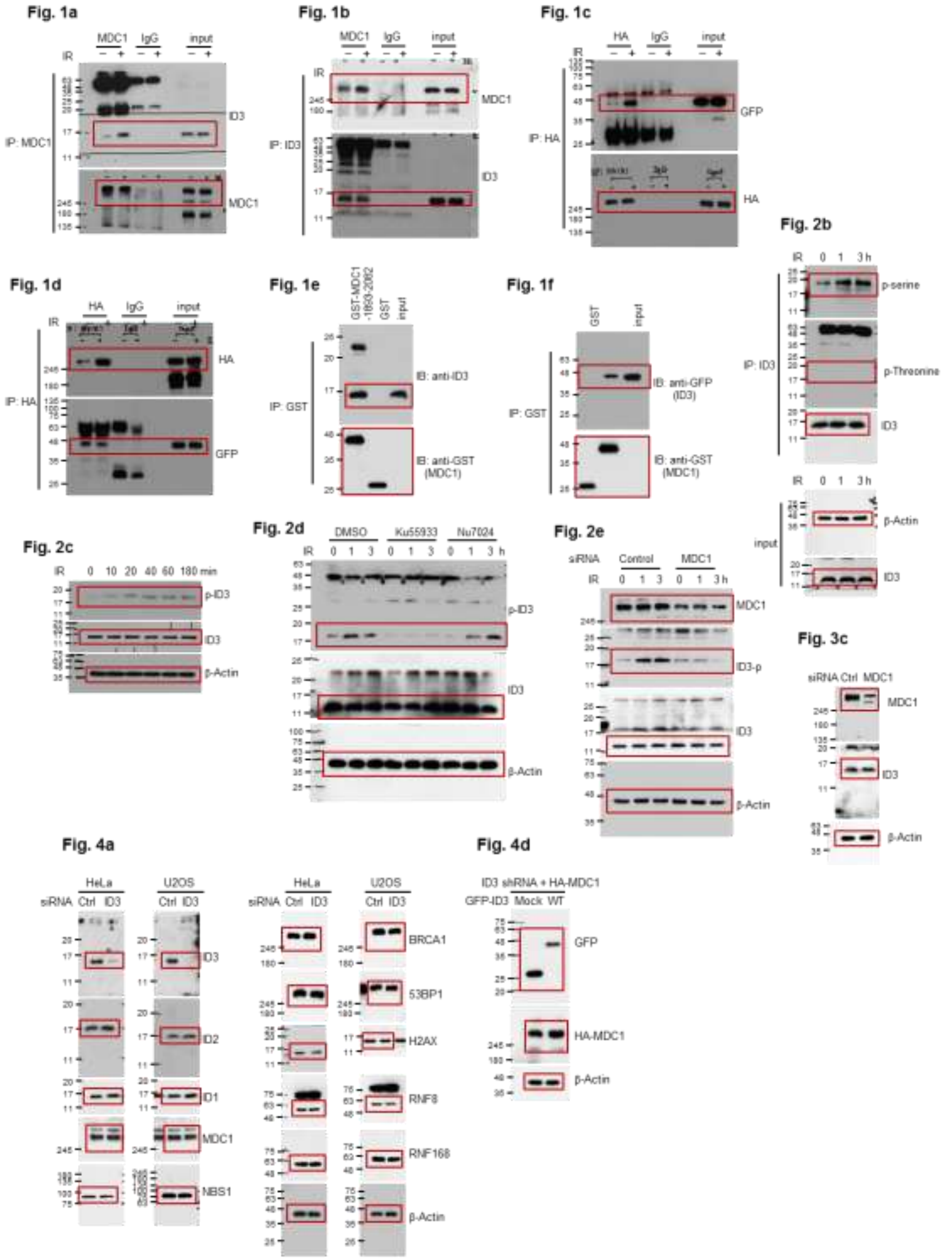

Supplementary Figure 14. Full scans of Western blots shown in main Figures. Cropped areas are marked by red color.

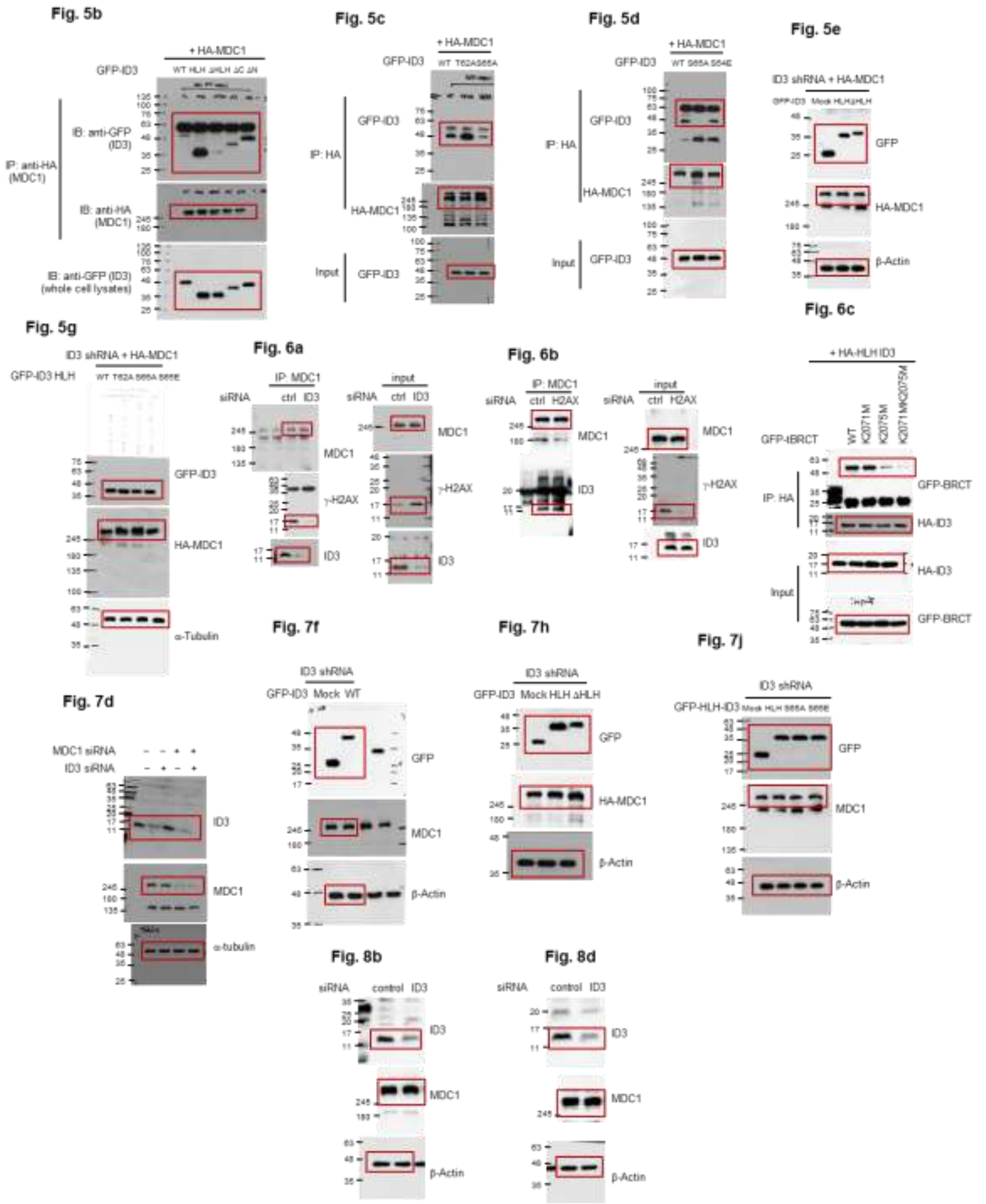

Supplementary Figure 14. Full scans of Western blots shown in main Figures. Cropped areas are marked by red color.

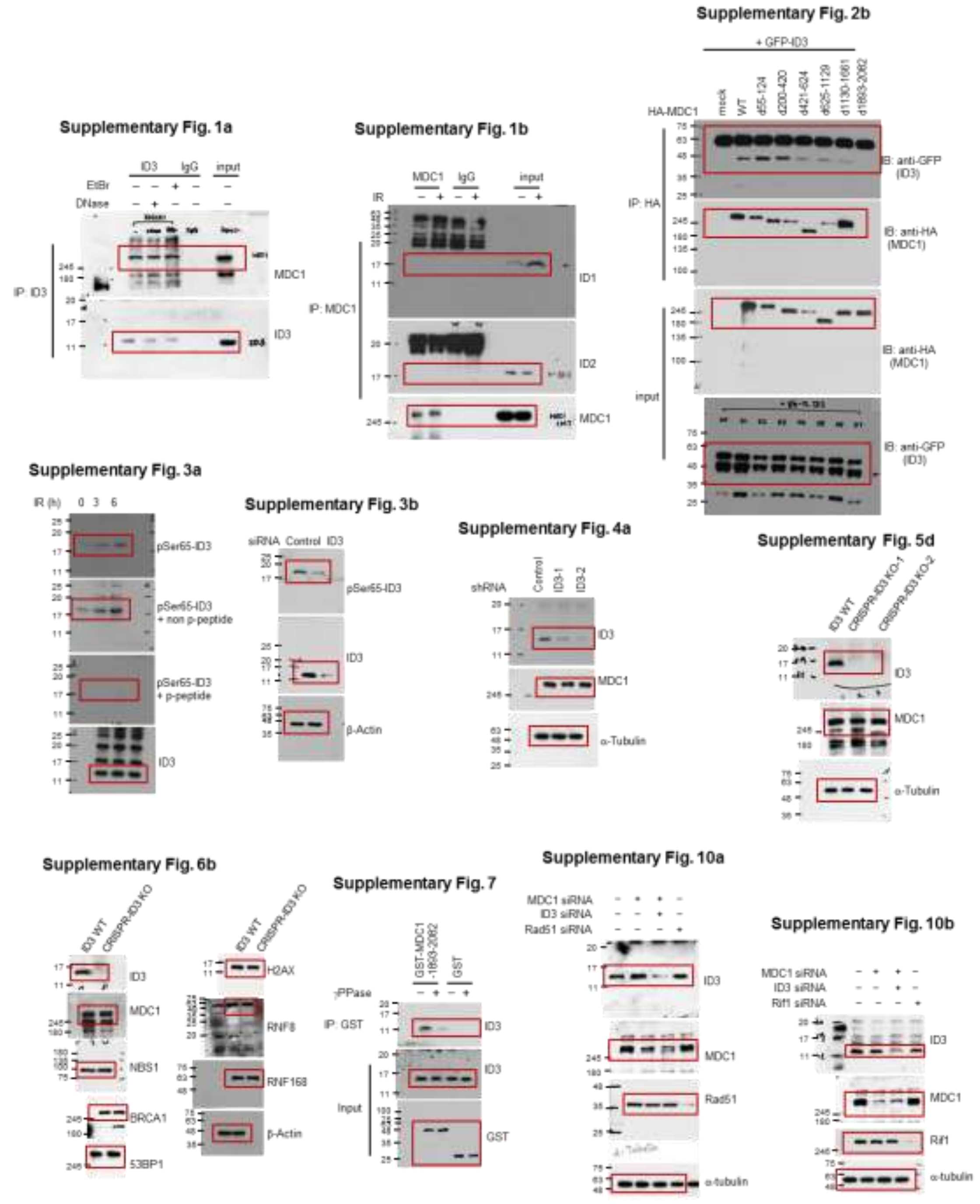

Supplementary Figure 15. Full scans of Western blots shown in Supplementary Figures. Cropped areas are marked by red color.

Supplementary Table 1. Lists the antibodies used in this study. WB: Western blot; IF: immunofluorescence; IP: immunoprecipitation

Antibody	Species	Application			Reference / Suppliers	
		WB	IF	IP	Suppliers	Cat. No
MDC1	rabbit	1:1000			Novus	NB100-397
MDC1	rabbit	1:1000	1:200	1:1000	Manufactured in my Lab.	
MDC1	rabbit	1:1000			Abcam	Ab11170
ID1	rabbit	1:500			Santa cruz	sc-427
ID2	goat	1:500			Santa cruz	sc-26328
ID3	rabbit	1:500		1:1000	Santa cruz	sc-13046
ID3	mouse	1:100			Santa cruz	sc-56712
HA	mouse	1:500			Santa cruz	sc-7392
HA	rabbit	1:500	1:50	1:1000	Santa cruz	sc-805
GFP	mouse	1:1000			Santa cruz	sc-9996
GFP	rabbit	1:1000	1:200	1:1000	Santa cruz	sc-8334
GST	mouse	1:1000			Santa cruz	sc-138
phospho-serine	mouse	1:1000			Sigma	P5747
phospho-Threonine	mouse	1:1000			Sigma	P3555
α-tubulin	mouse	1:2000			Neomarkers	MS-581-PO
phospho-ID3 (Ser65)	rabbit	1:1000	1:100		Manufactured in my Lab.	
γ-H2AX (Ser139)	mouse	1:1000	1:200		Millipore	05-636-1
NBS1	mouse	1:1000	1:100		BD Biosciences	611870
BRCA1	rabbit	1:500	1:50		Santa cruz	sc-642
53BP1	mouse	1:1000	1:200		BD Biosciences	612523
H2AX	rabbit	1:500	1:200		Calbiochem	DR1016
RNF8	goat	1:1000	1:200		Abcam	ab15850
RNF168	mouse	1:1000	1:100		Abcam	ab58063
Rad51	rabbit	1:500			Santa cruz	sc-8349
RIF1	rabbit	1:1000			Bethyl	A300-567A
b-actin	mouse	1:2000			Santa cruz	sc-47778
Donky Anti-Rabbit IgG	donky	1:4000			Jackson immunoresearch	711-035-152
Goat Anti-Rabbit IgG	goat	1:4000			Jackson immunoresearch	111-035-008
Goat Anti-Mouse IgG	goat	1:4000			Jackson immunoresearch	115-035-008
Sheep Anti-Mouse IgG	sheep	1:4000			Jackson immunoresearch	515-035-071
Donky Anti-Goat IgG	donky	1:4000			Jackson immunoresearch	705-035-003
Rabbit Anti-Goat IgG	rabbit	1:4000			Jackson immunoresearch	305-035-046
Alexa Fluor 488 Chicken Anti-Rabbit IgG(H+L)	chicken		1:200		Invitrogen	A-21441
Alexa Fluor 488 Chicken Anti-Mouse IgG(H+L)	chicken		1:200		Invitrogen	A-21200
Alexa Fluor 594 Chicken Anti-Goat IgG(H+L)	chicken		1:200		Invitrogen	A21468
Alexa Fluor 594 Chicken Anti-Mouse IgG(H+L)	chicken		1:200		Invitrogen	A-21201
Alexa Fluor 594 Chicken Anti-Rabbit IgG(H+L)	chicken		1:200		Invitrogen	A-21442
