# Supplementary material for: Author Correction: ID3 regulates the MDC1-mediated DNA damage response in order to maintain genome stability
Source: Nat Commun. 2018 Jun 6;9:2284. doi: 10.1038/s41467-018-04599-6 (PMC5989224; doi:10.1038/s41467-018-04599-6)
Supplement: Supplementary file 1 — Supplementary Data 1 [file 41467_2018_4599_MOESM1_ESM.zip › Fig3b/Fig 3b.pptm]

## Slide 1
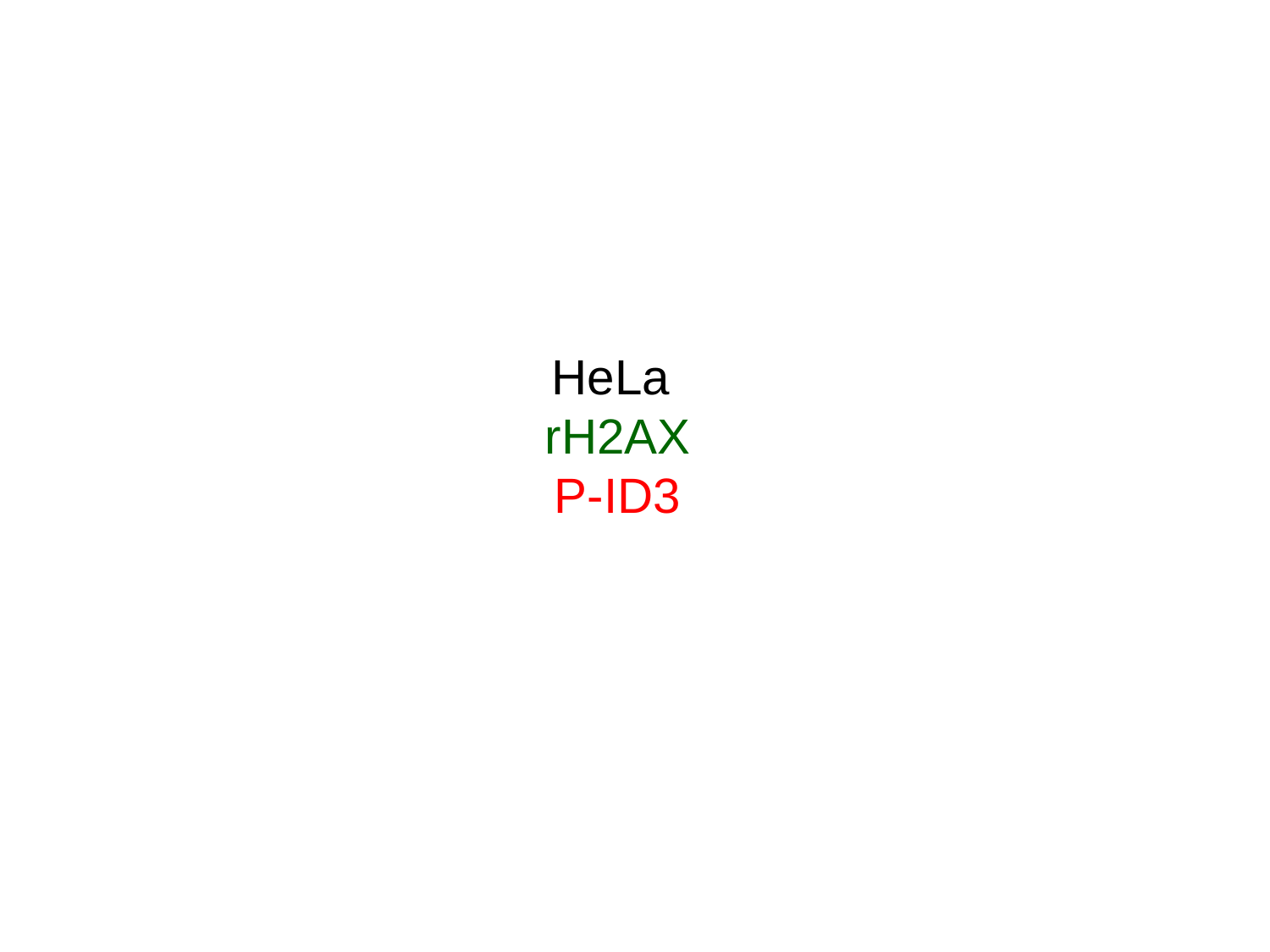

HeLa
rH2AX
P-ID3

## Slide 2
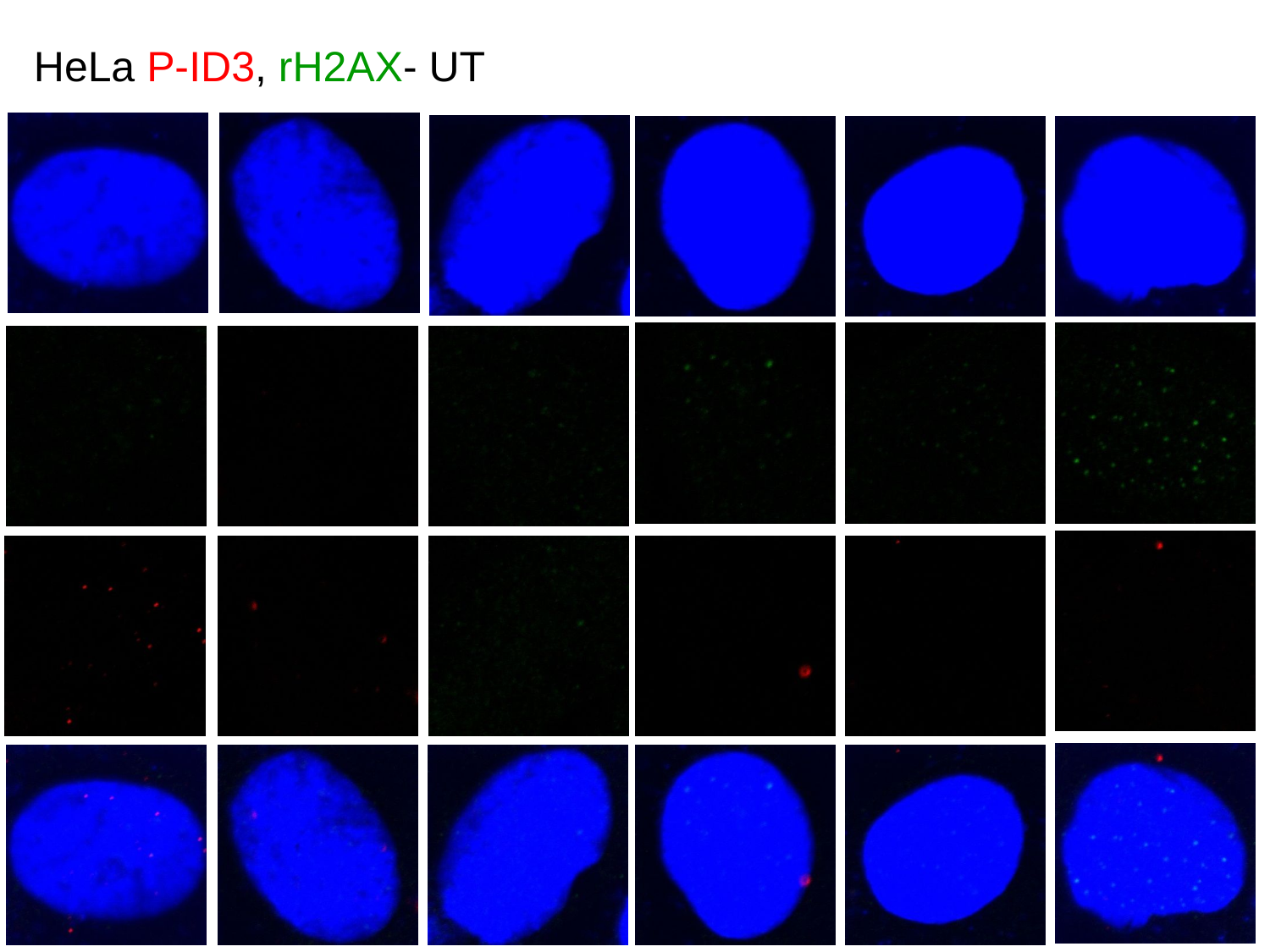

HeLa P-ID3, rH2AX- UT

## Slide 3
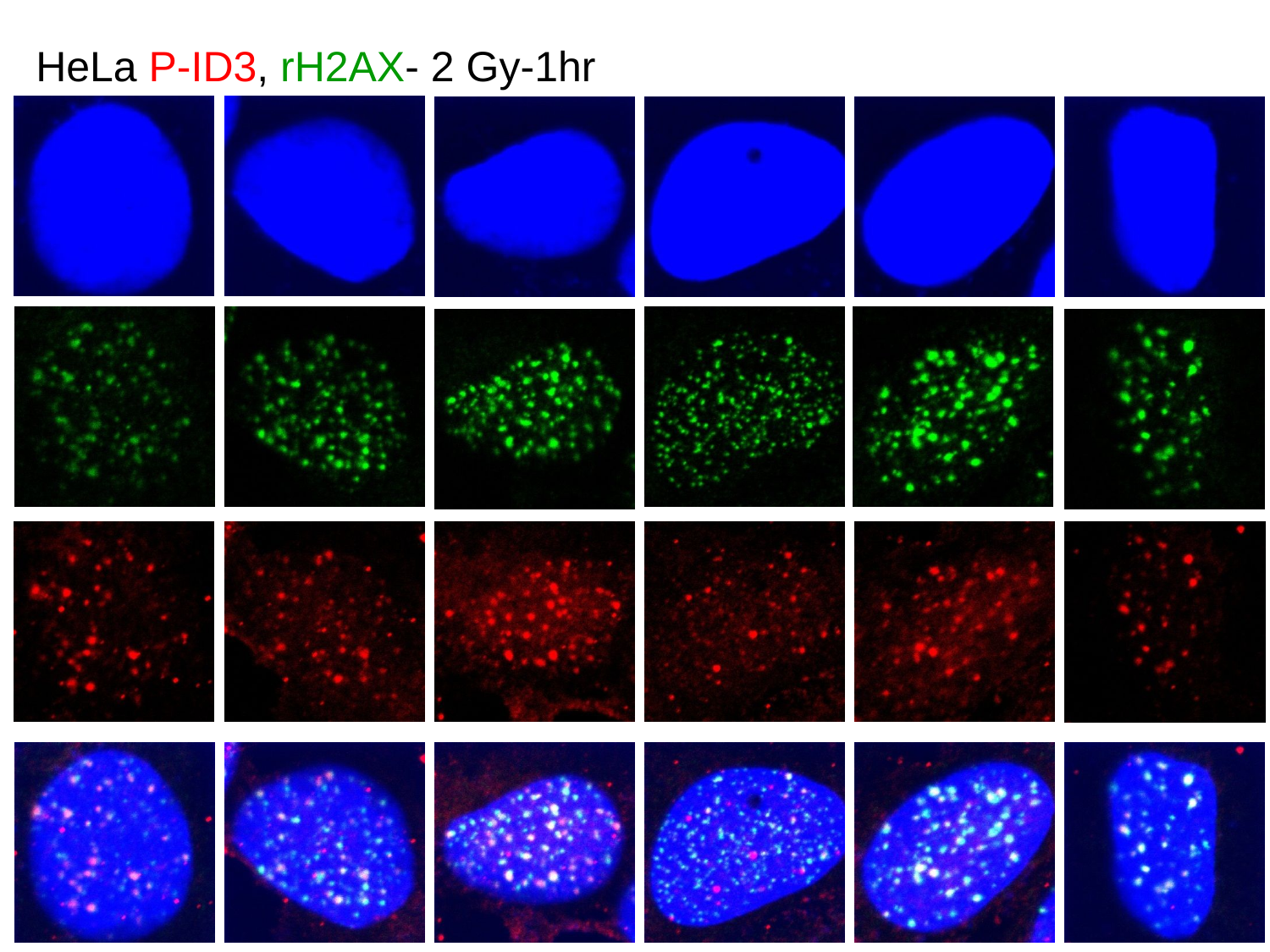

HeLa P-ID3, rH2AX- 2 Gy-1hr

## Slide 4
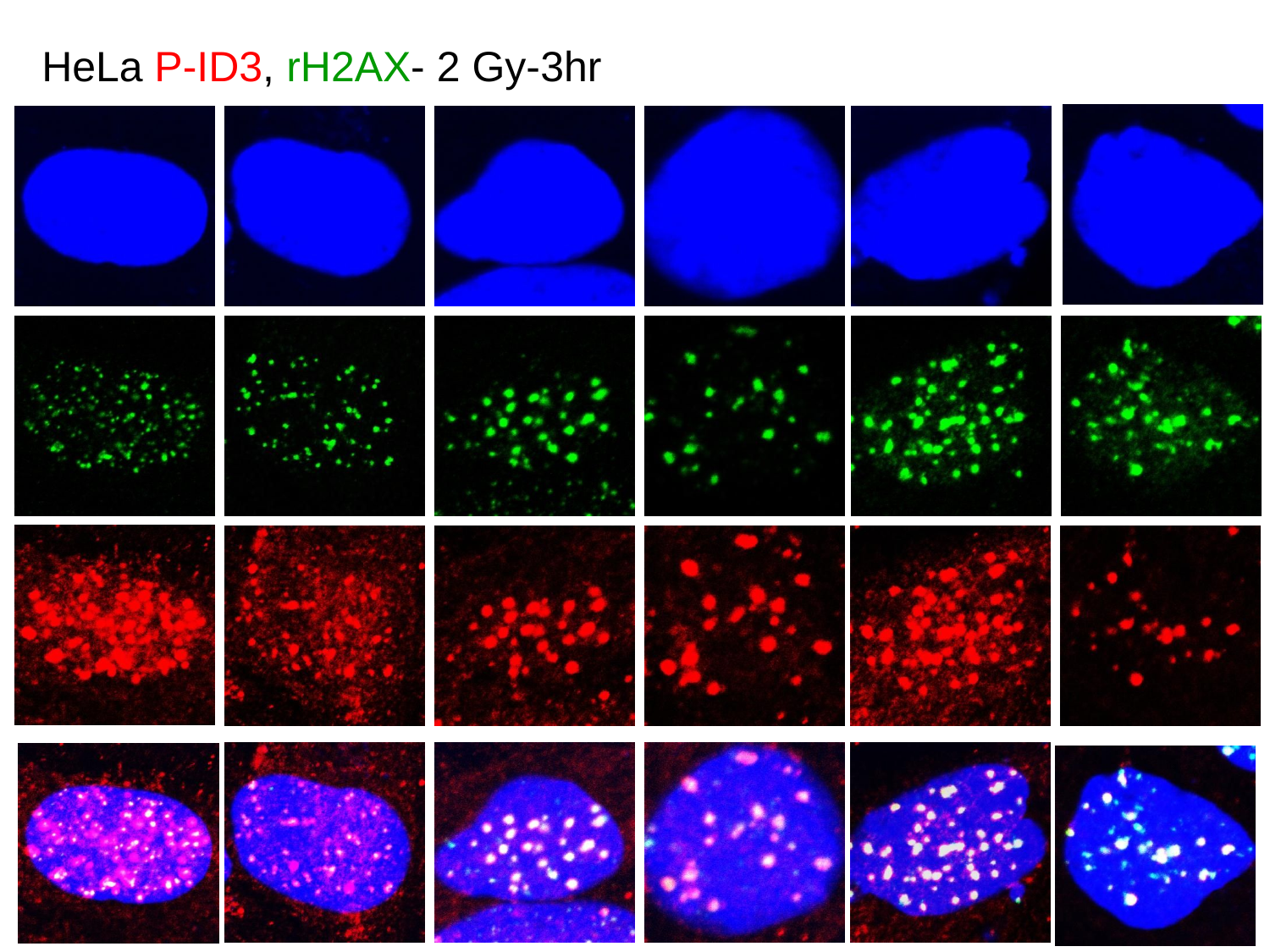

HeLa P-ID3, rH2AX- 2 Gy-3hr

## Slide 5
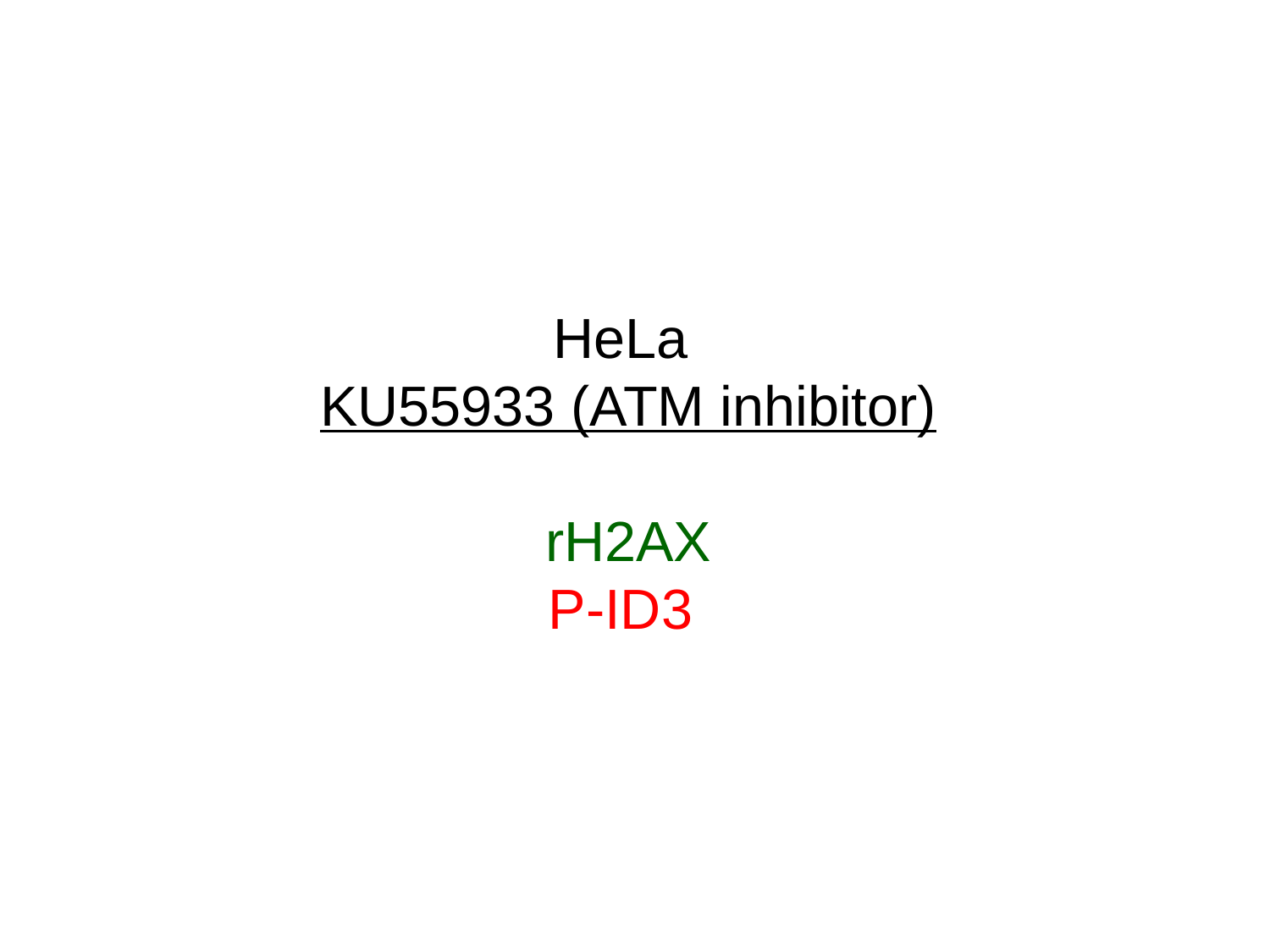

HeLa
KU55933 (ATM inhibitor)
rH2AX
P-ID3

## Slide 6
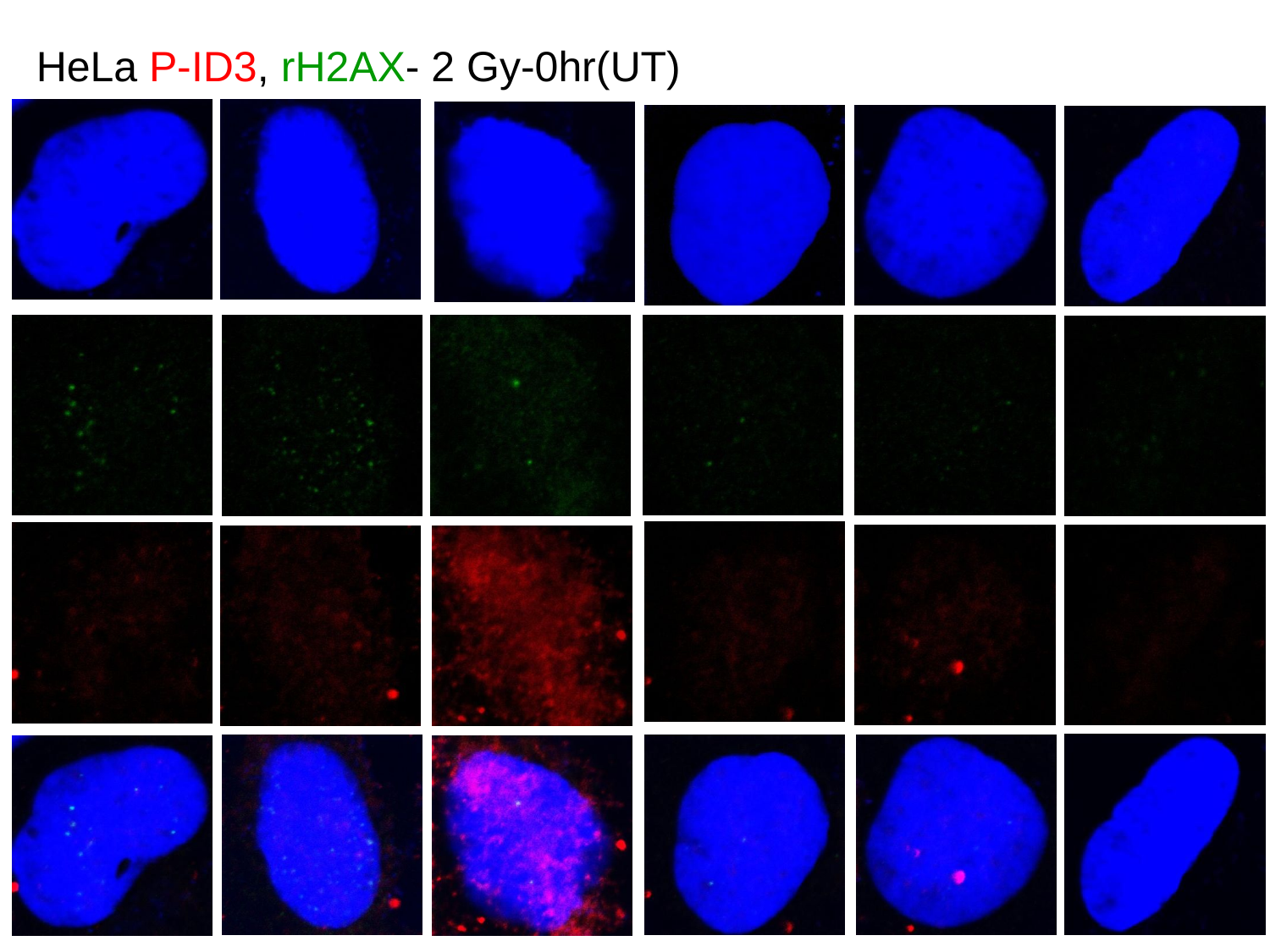

HeLa P-ID3, rH2AX- 2 Gy-0hr(UT)

## Slide 7
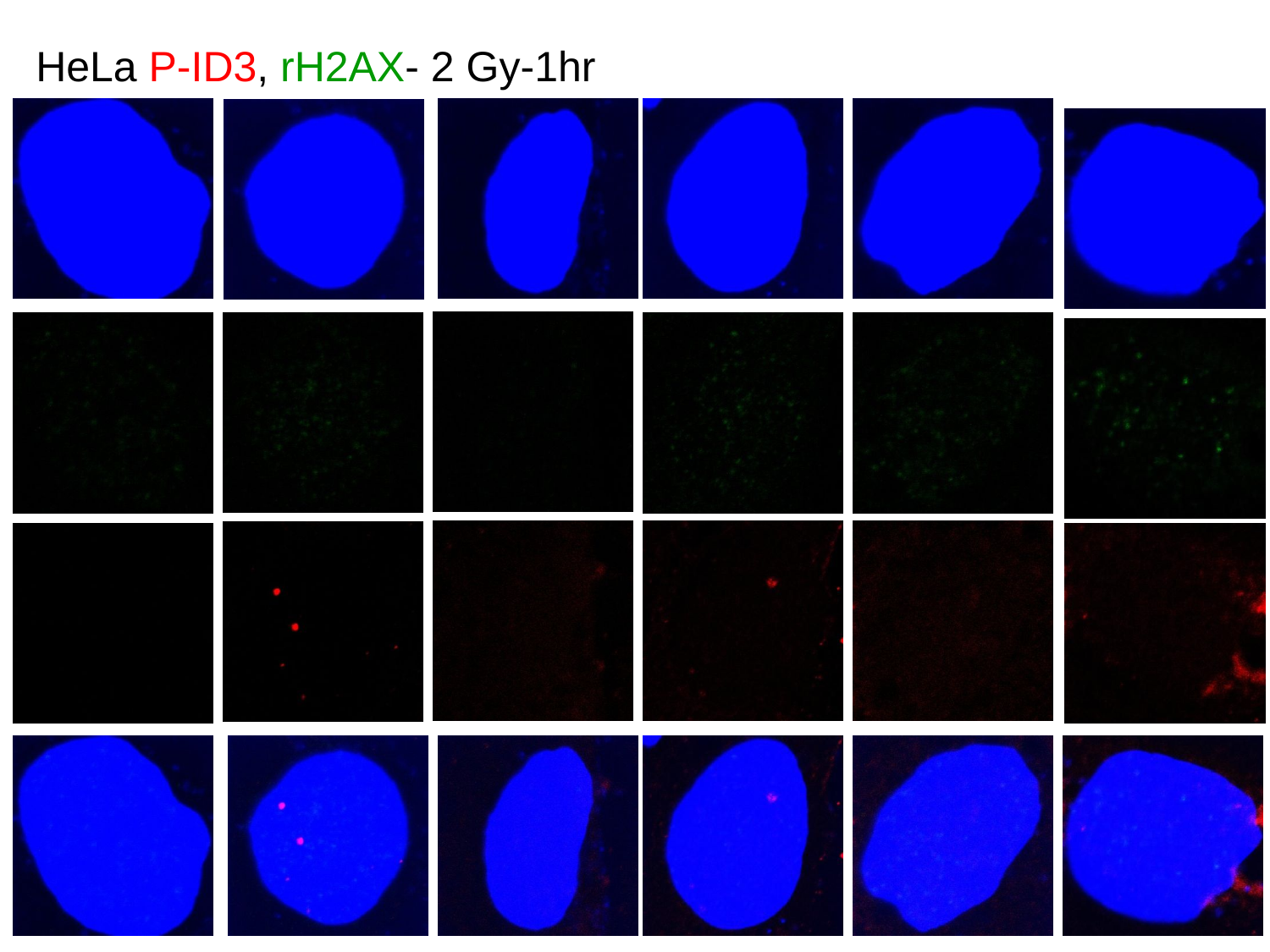

HeLa P-ID3, rH2AX- 2 Gy-1hr

## Slide 8
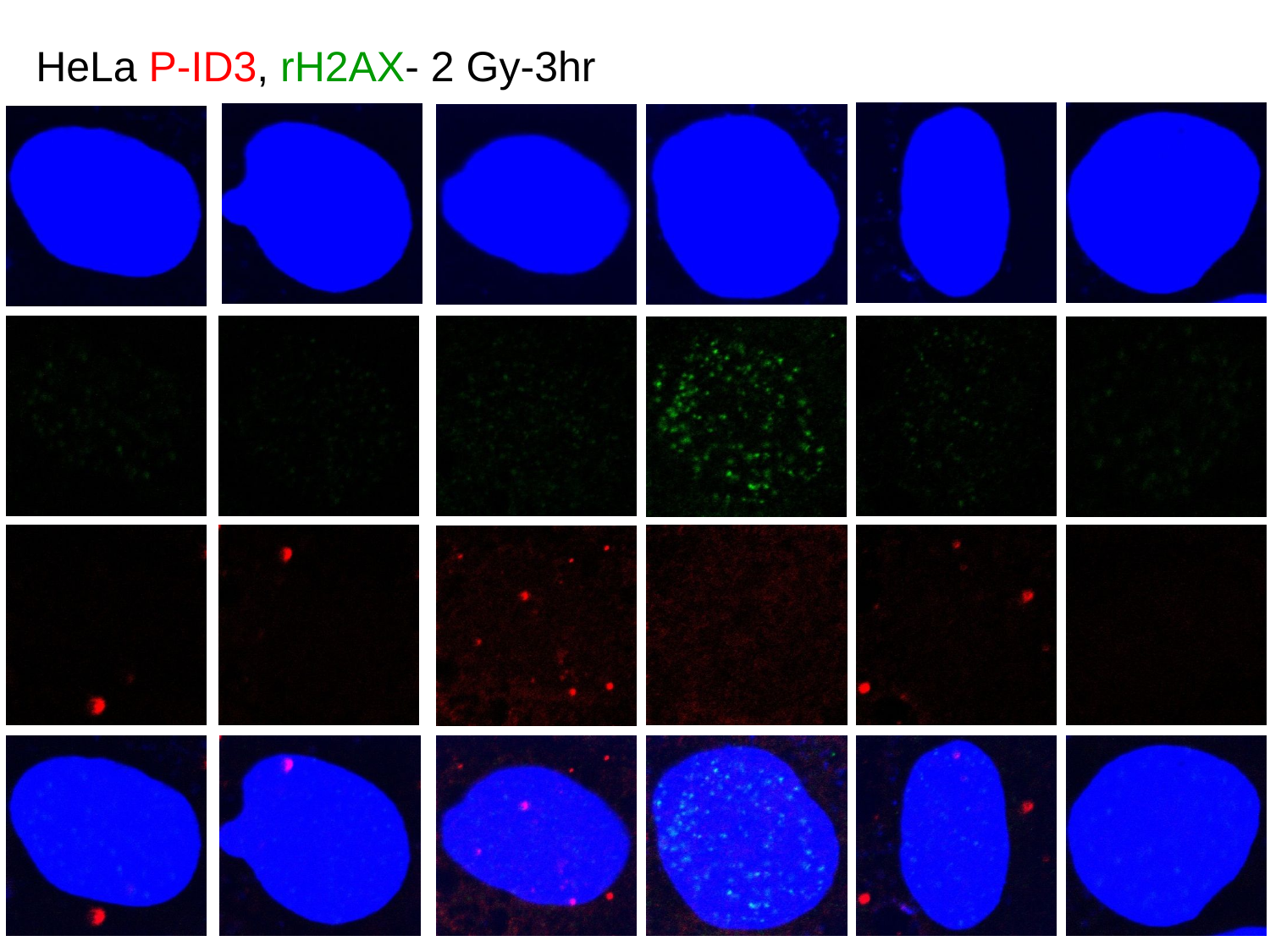

HeLa P-ID3, rH2AX- 2 Gy-3hr

## Slide 9
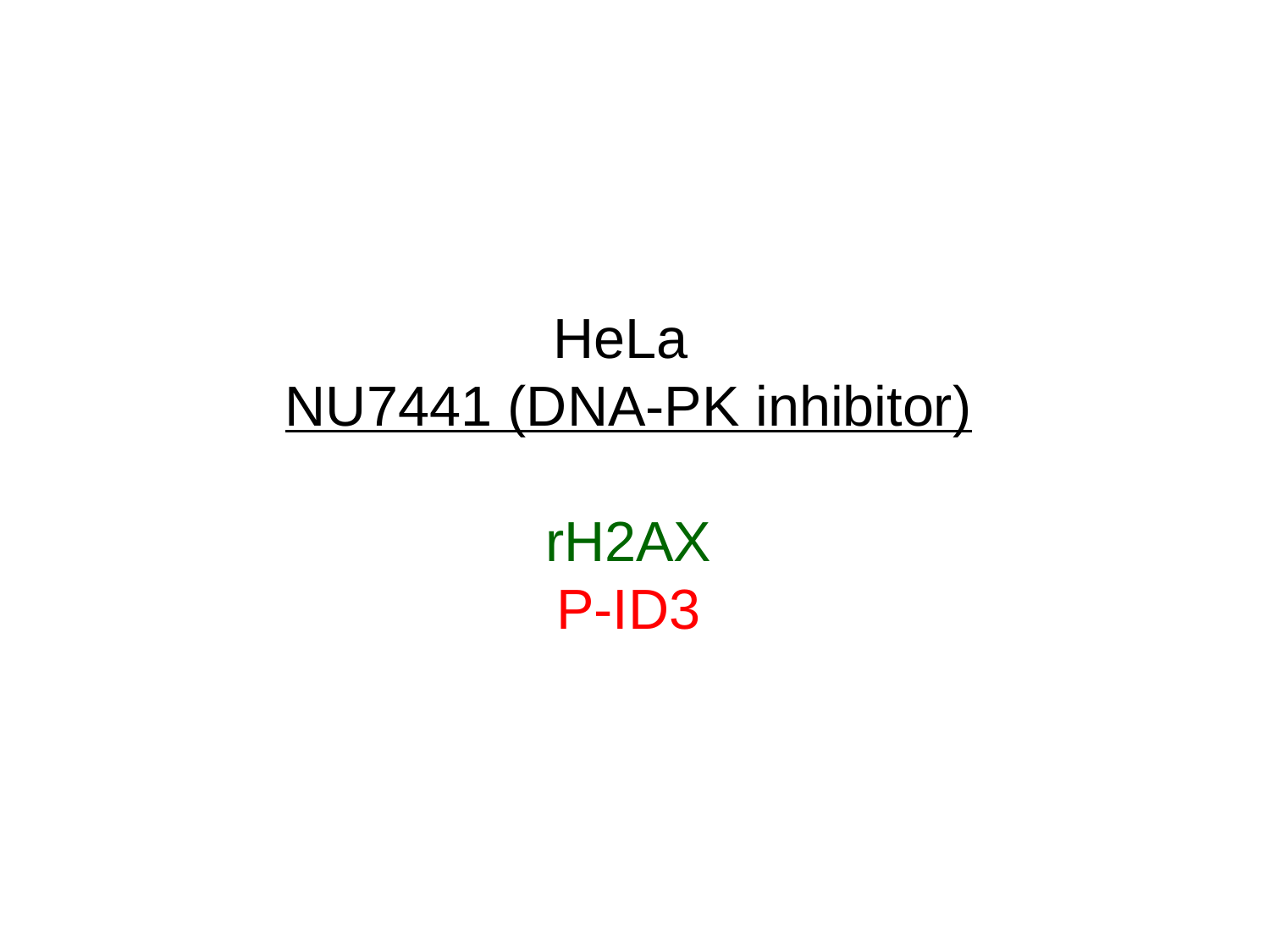

HeLa
NU7441 (DNA-PK inhibitor)
rH2AX
P-ID3

## Slide 10
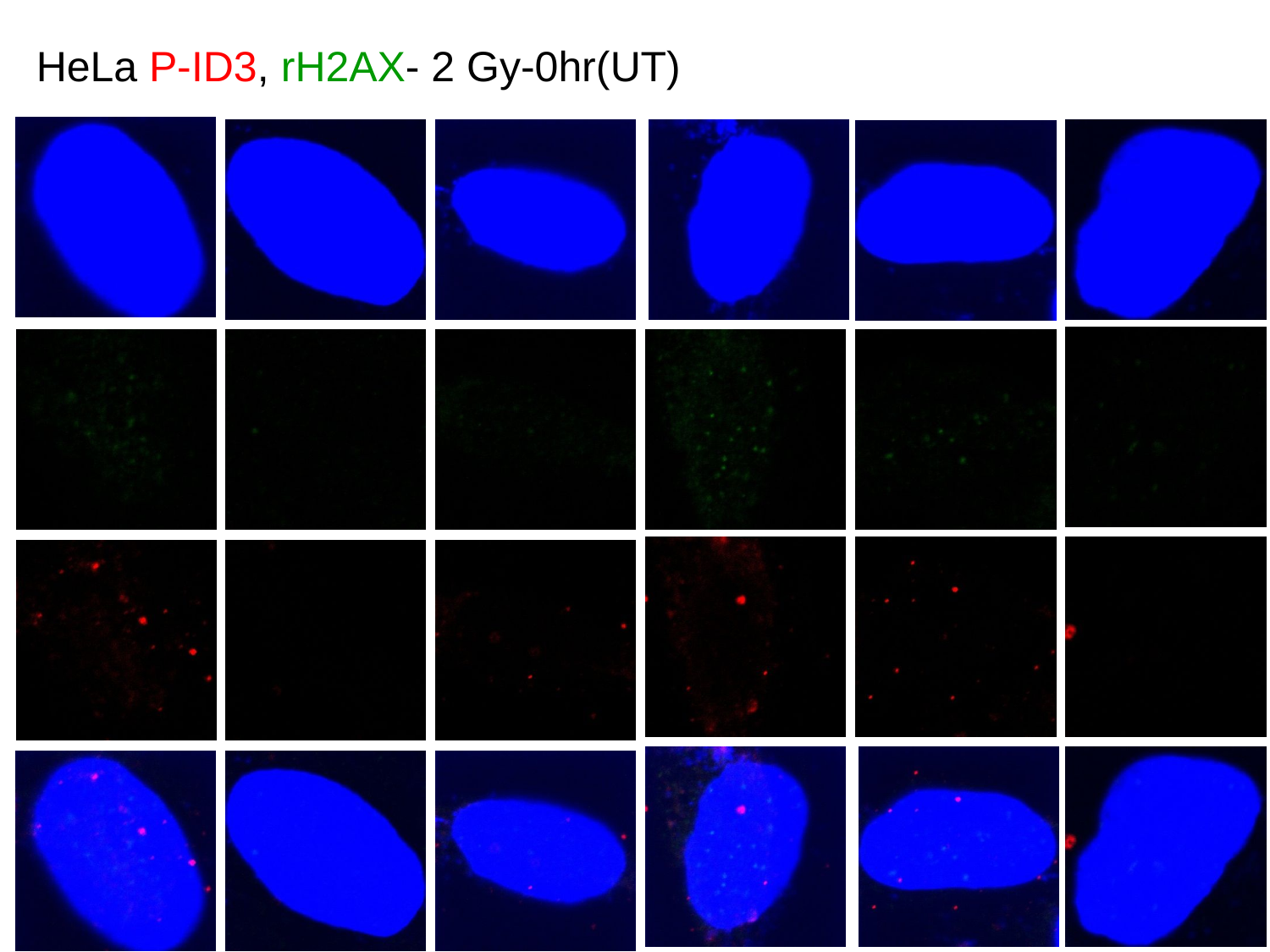

HeLa P-ID3, rH2AX- 2 Gy-0hr(UT)

## Slide 11
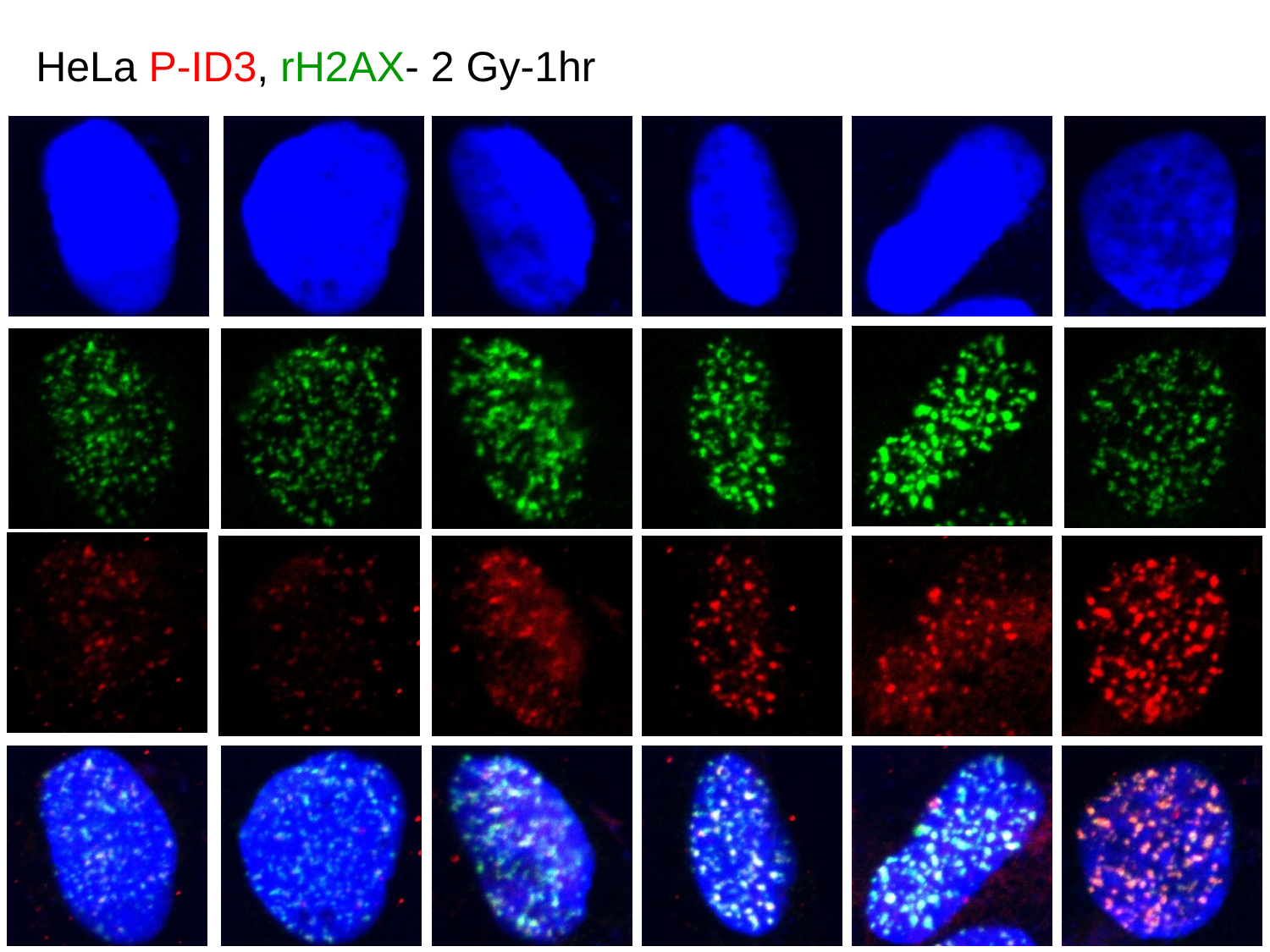

HeLa P-ID3, rH2AX- 2 Gy-1hr

## Slide 12
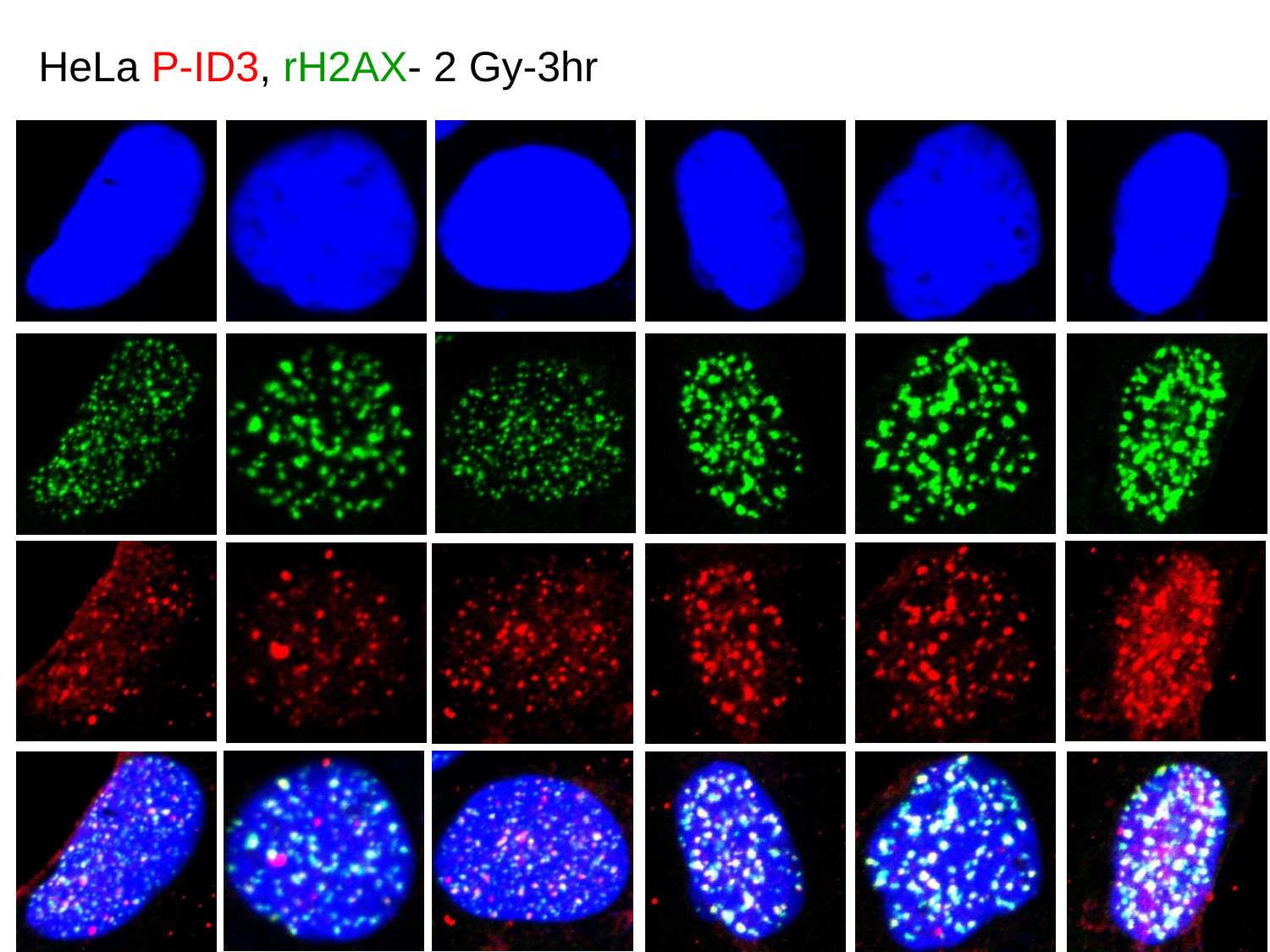

HeLa P-ID3, rH2AX- 2 Gy-3hr

## Slide 13
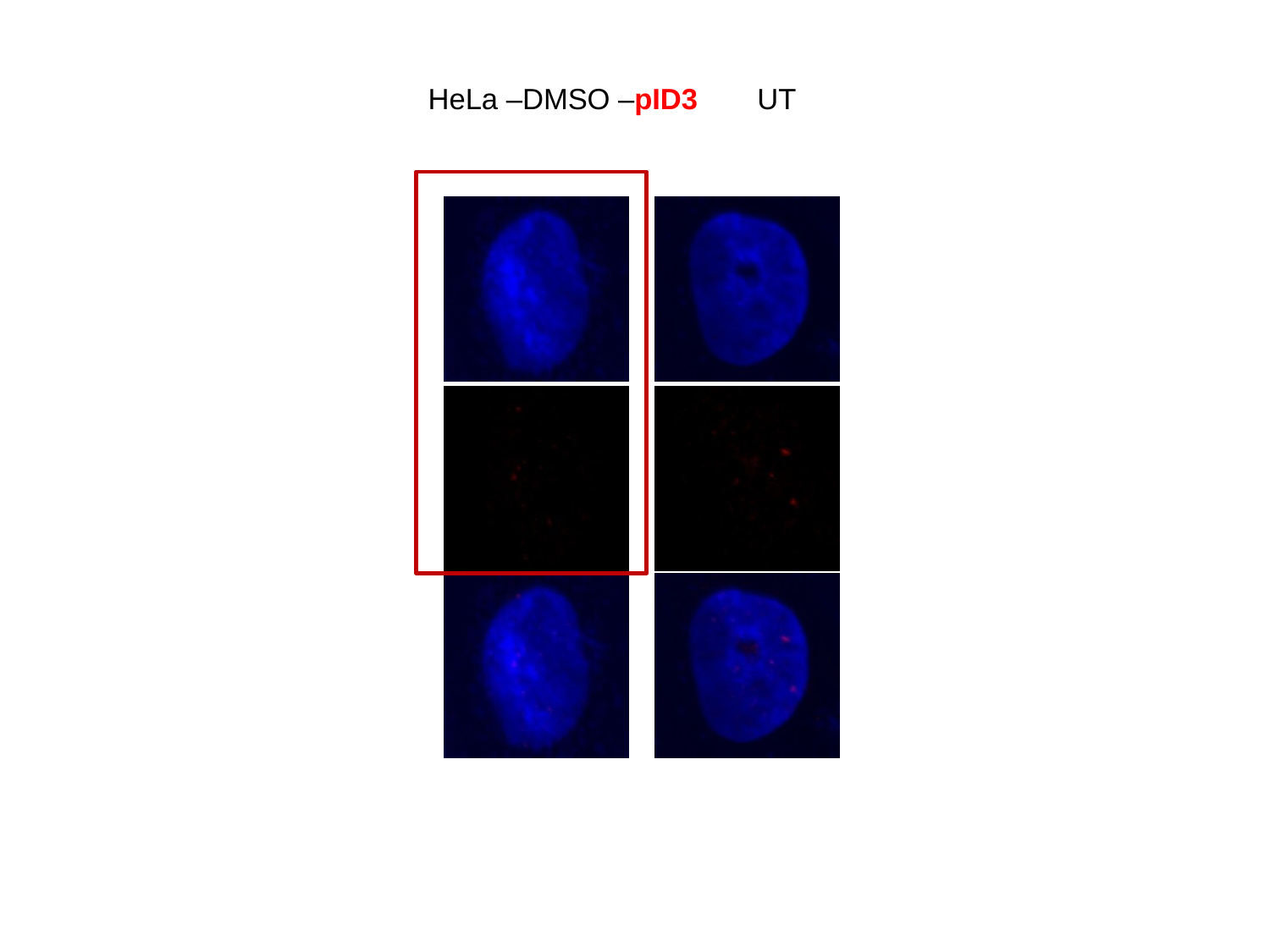

UT
HeLa –DMSO –pID3

## Slide 14
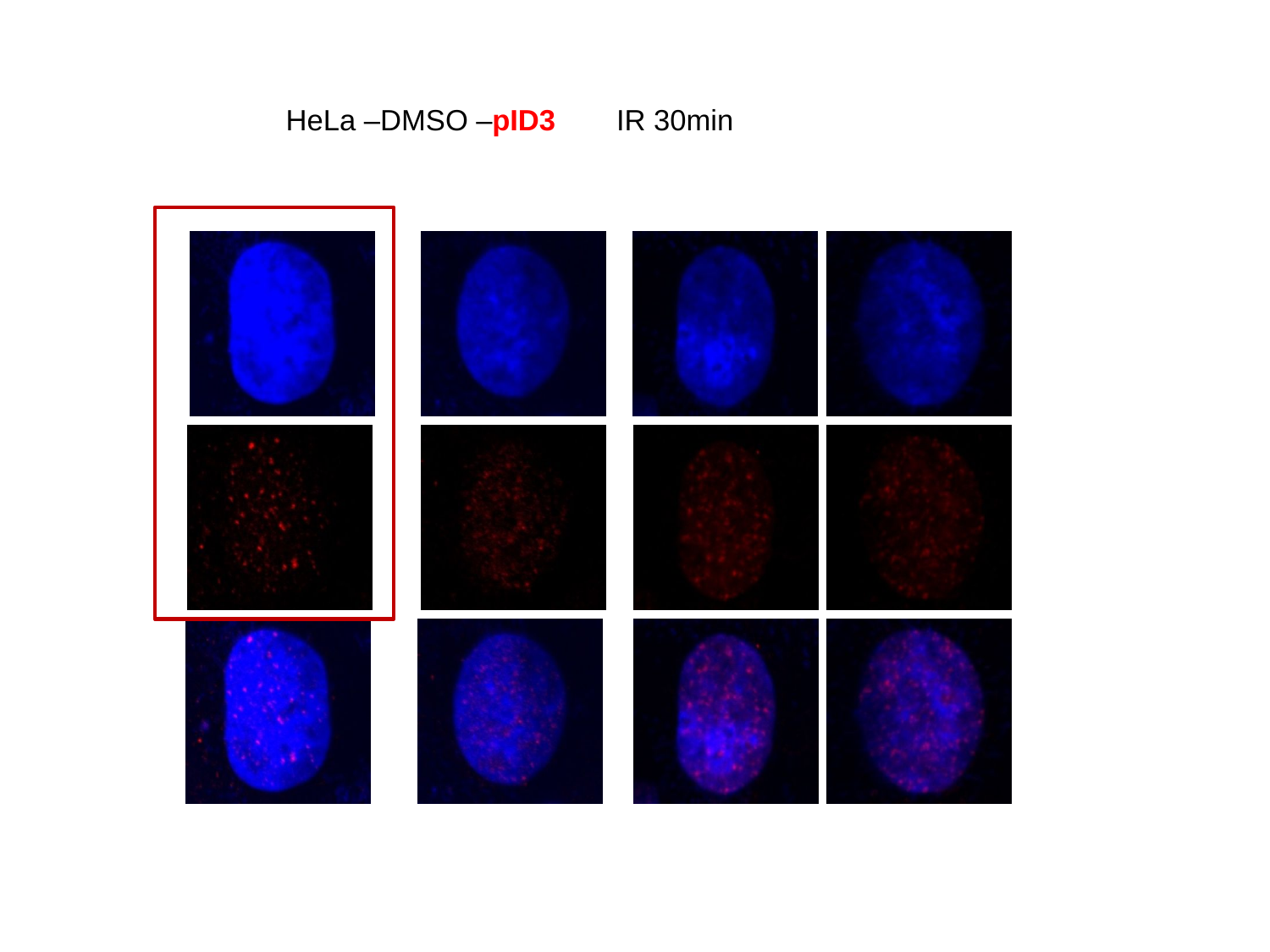

HeLa –DMSO –pID3
IR 30min

## Slide 15
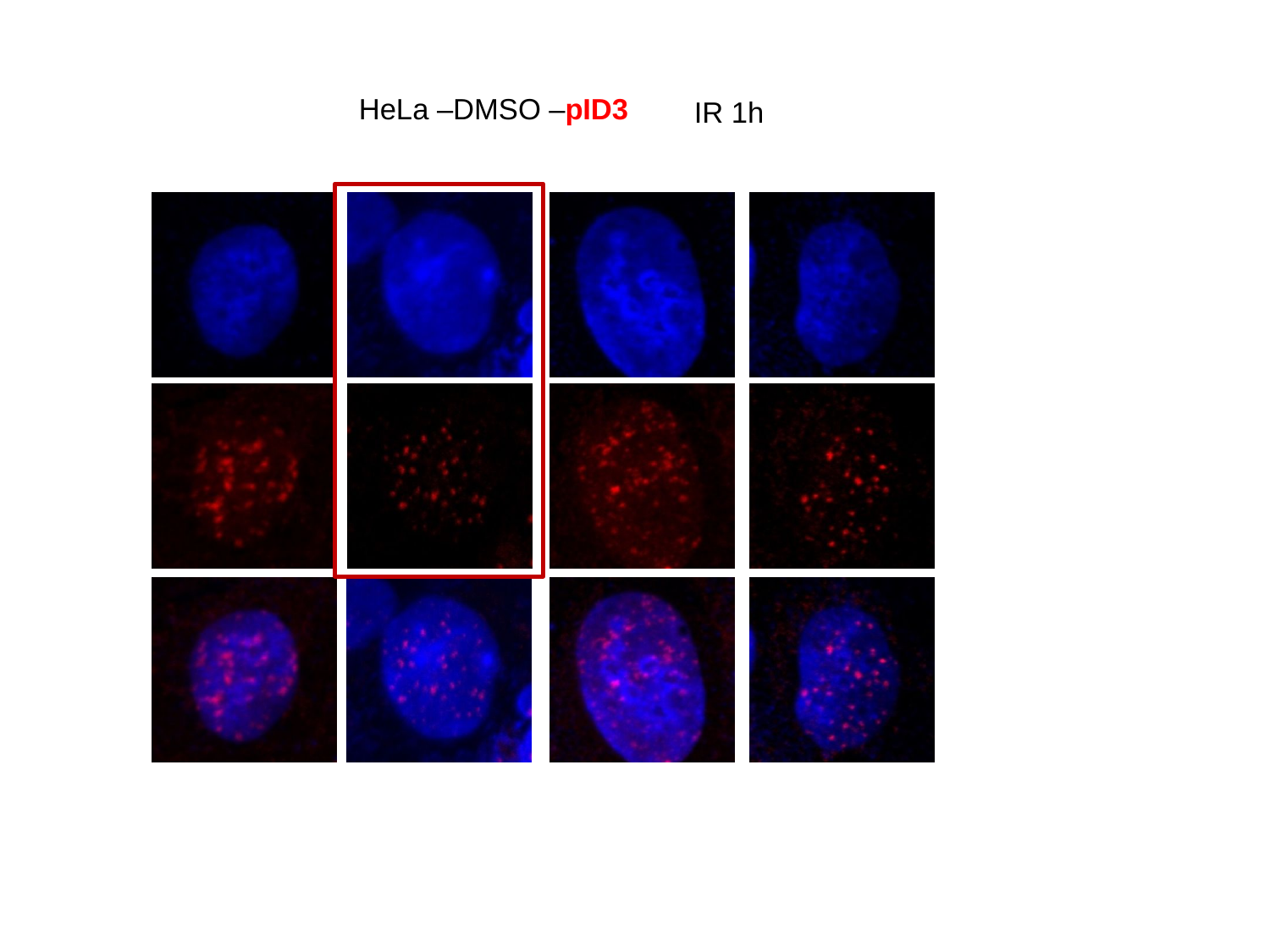

HeLa –DMSO –pID3
IR 1h

## Slide 16
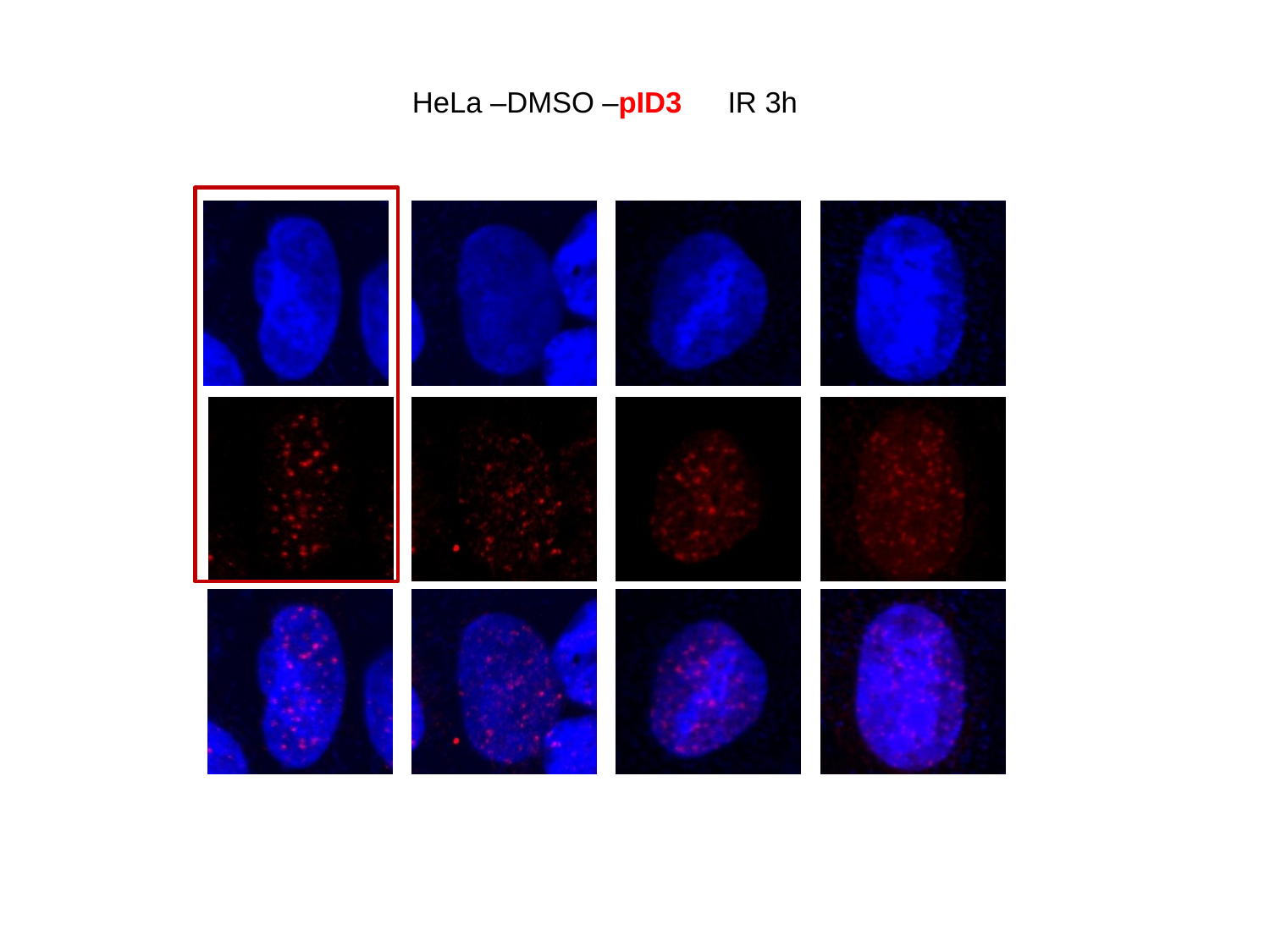

HeLa –DMSO –pID3
IR 3h

## Slide 17
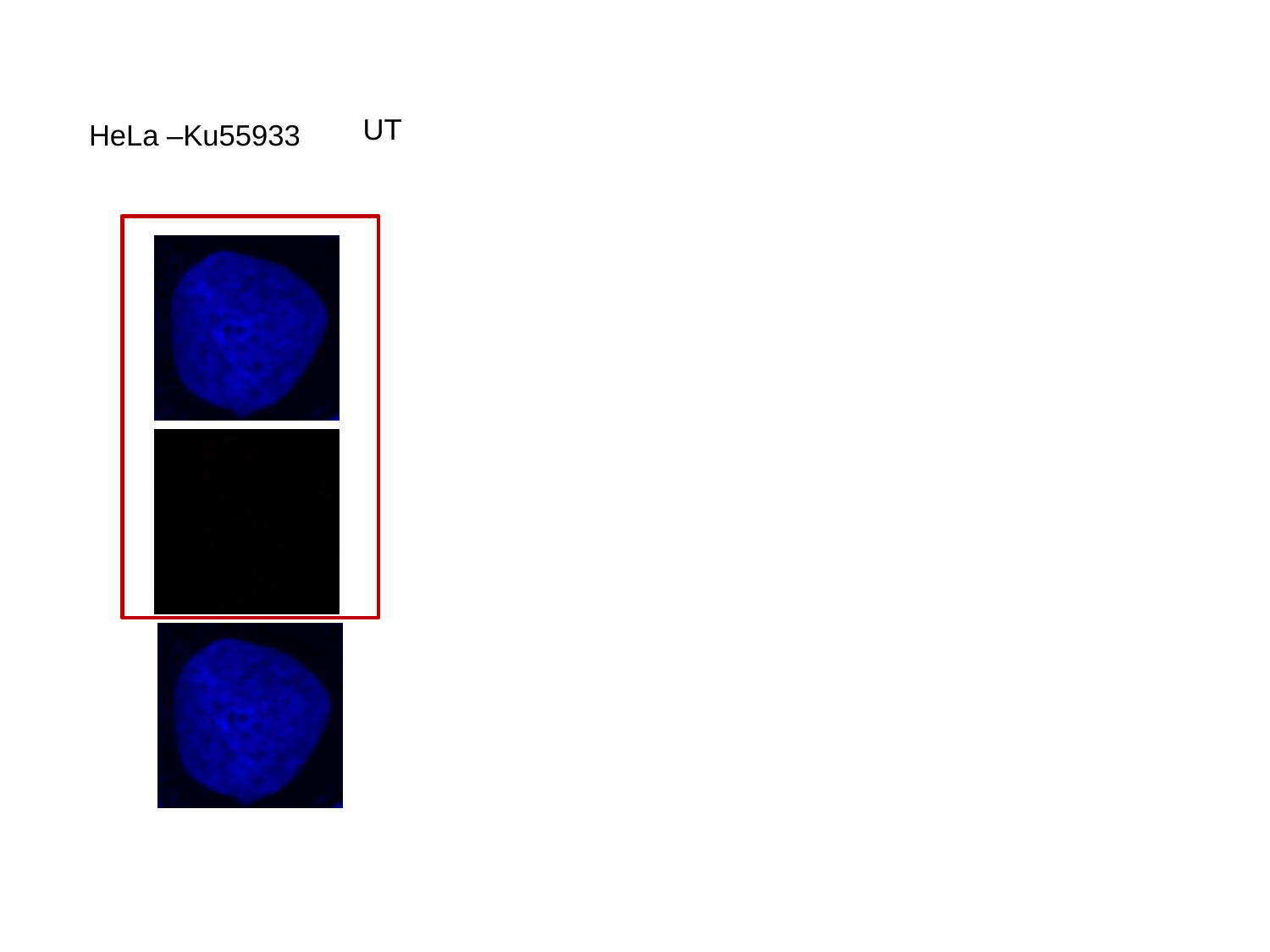

UT
HeLa –Ku55933

## Slide 18
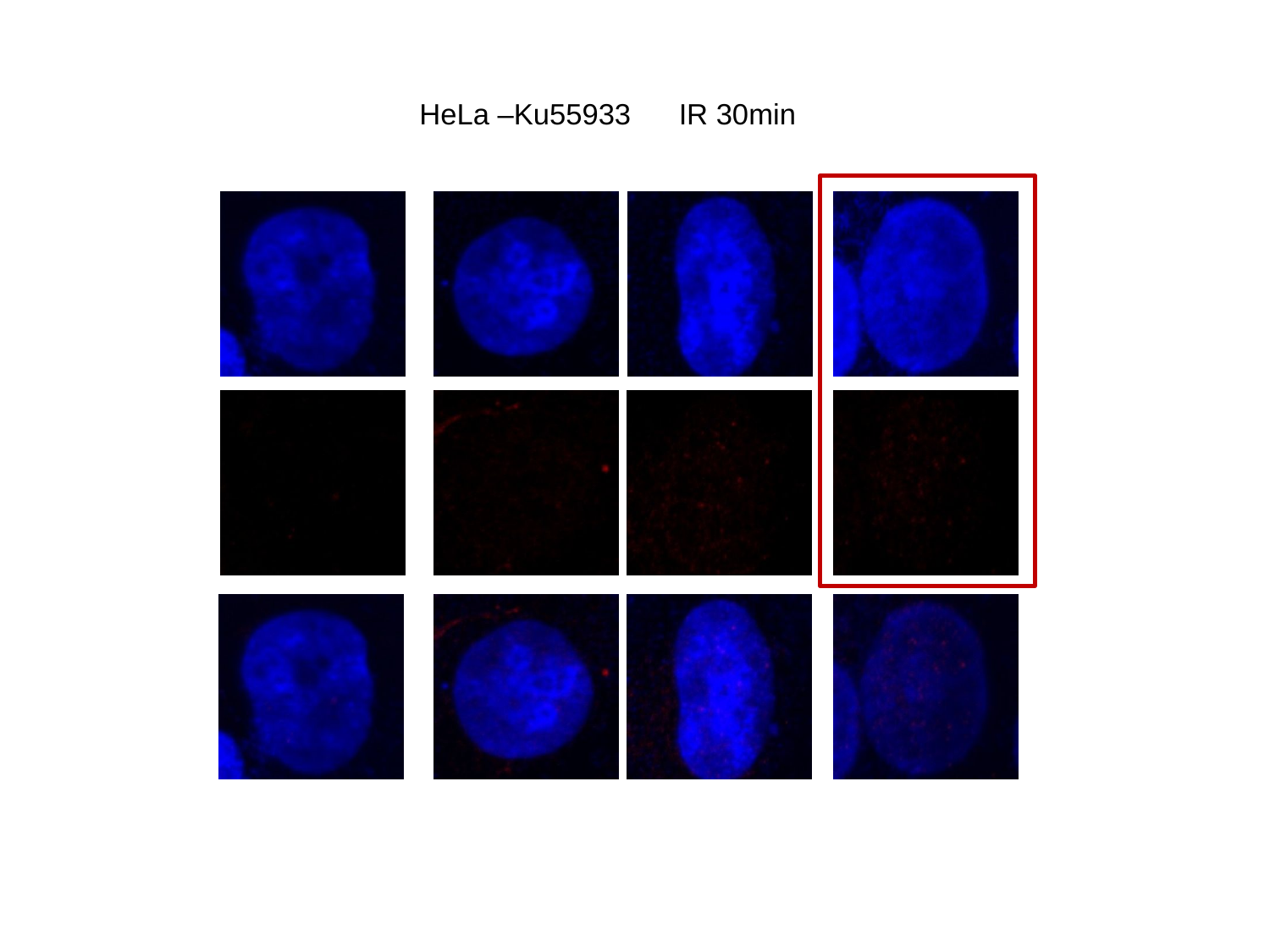

HeLa –Ku55933
IR 30min

## Slide 19
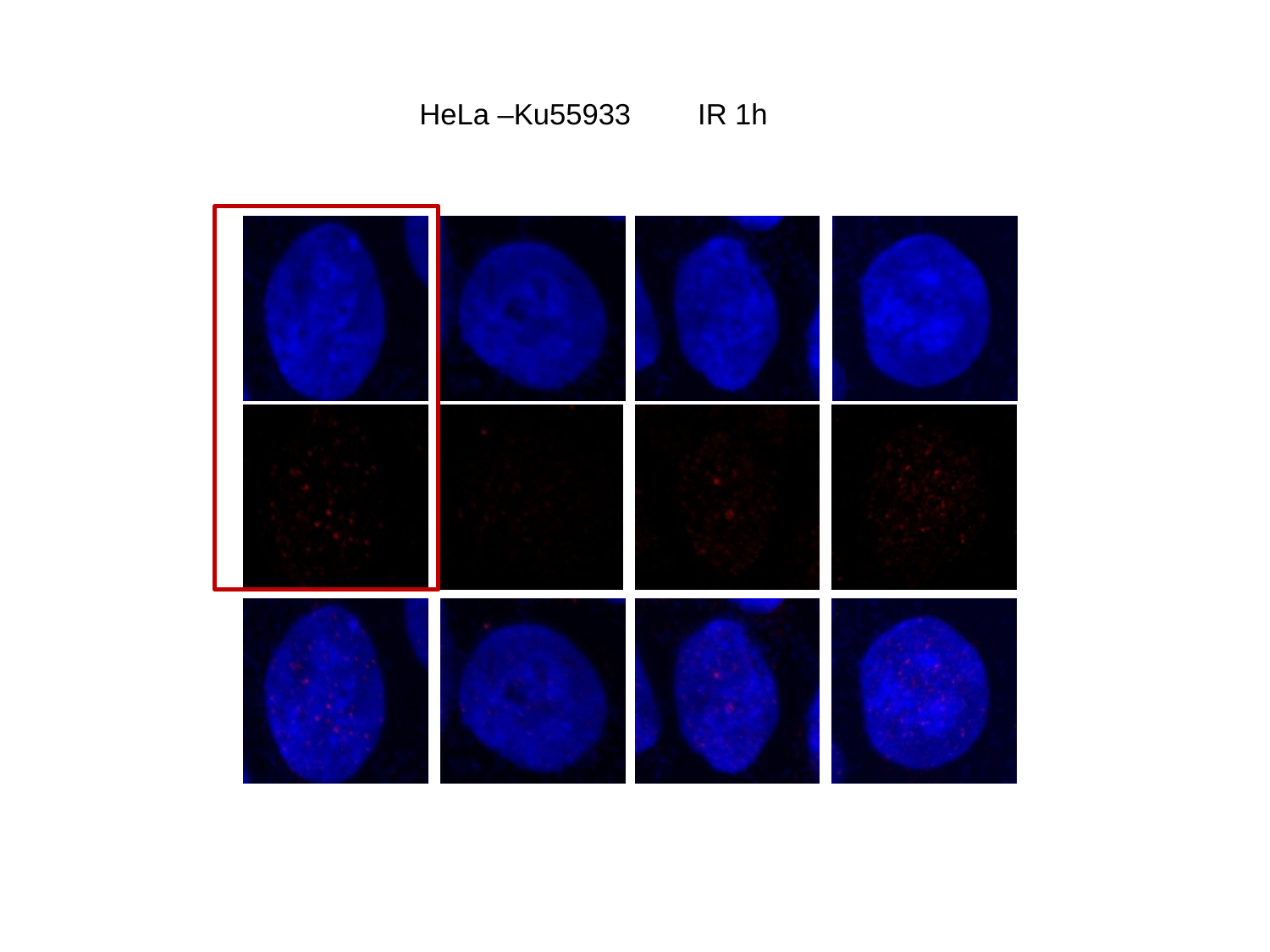

IR 1h
HeLa –Ku55933

## Slide 20
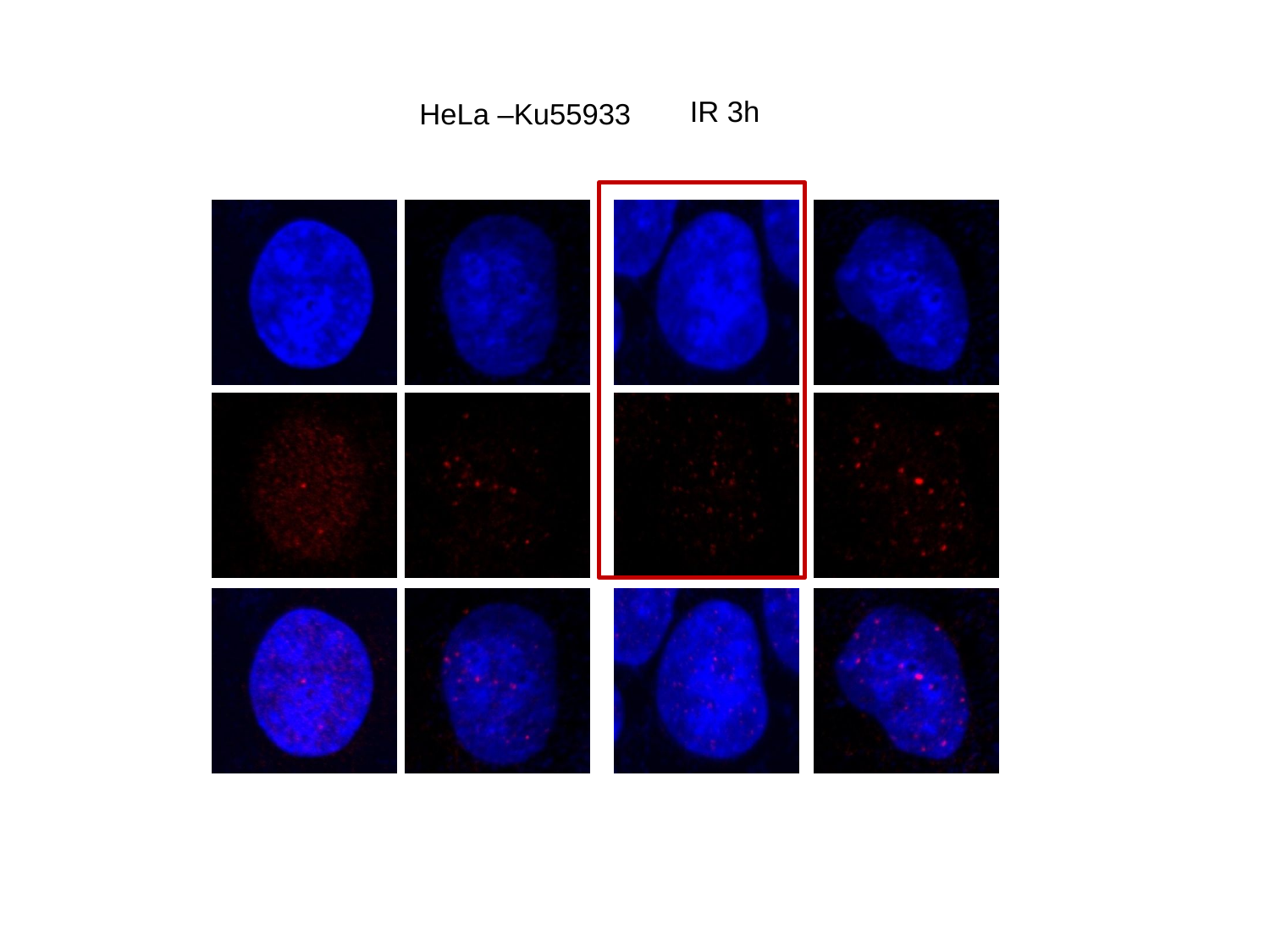

IR 3h
HeLa –Ku55933

## Slide 21
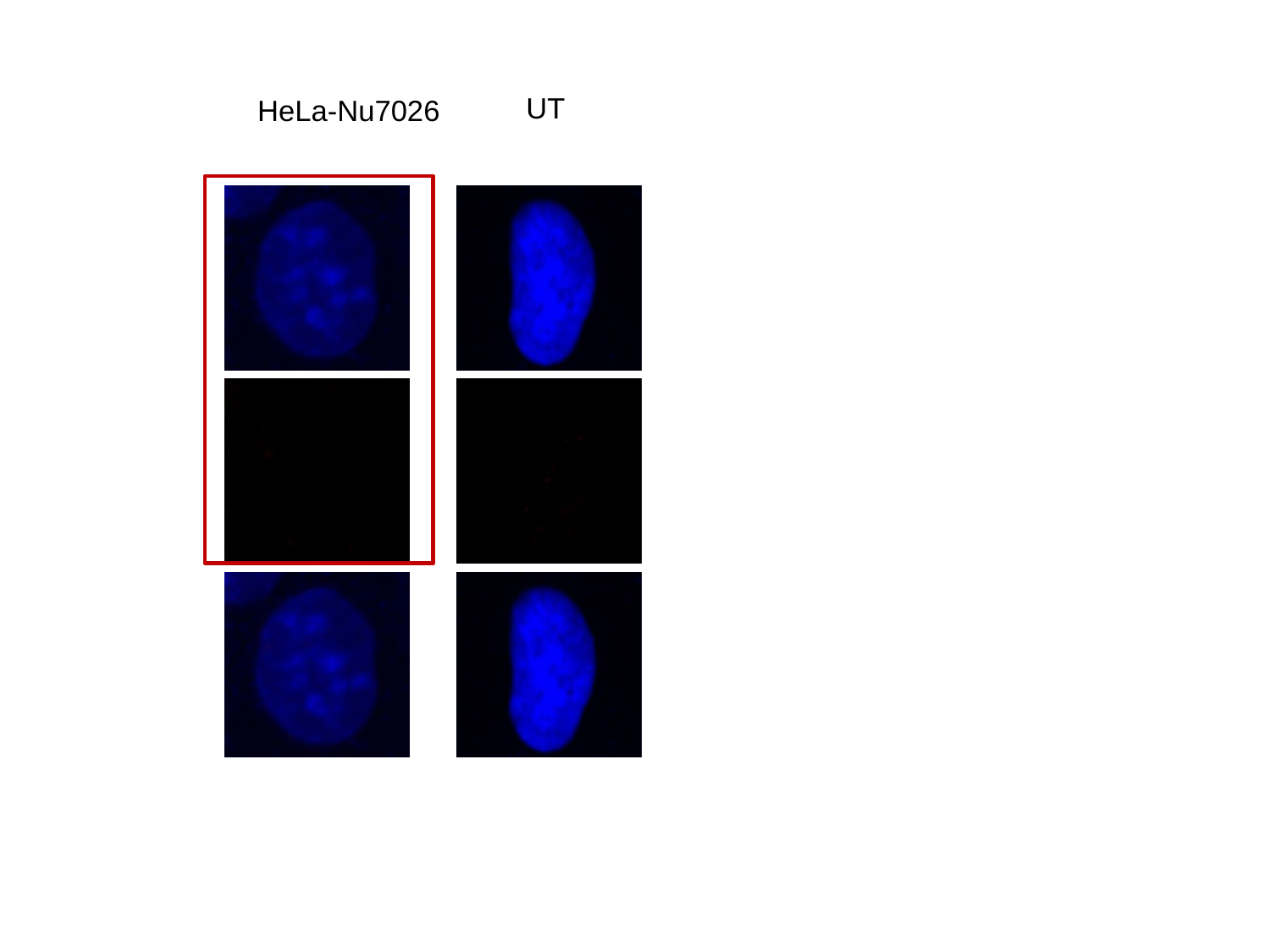

UT
HeLa-Nu7026

## Slide 22
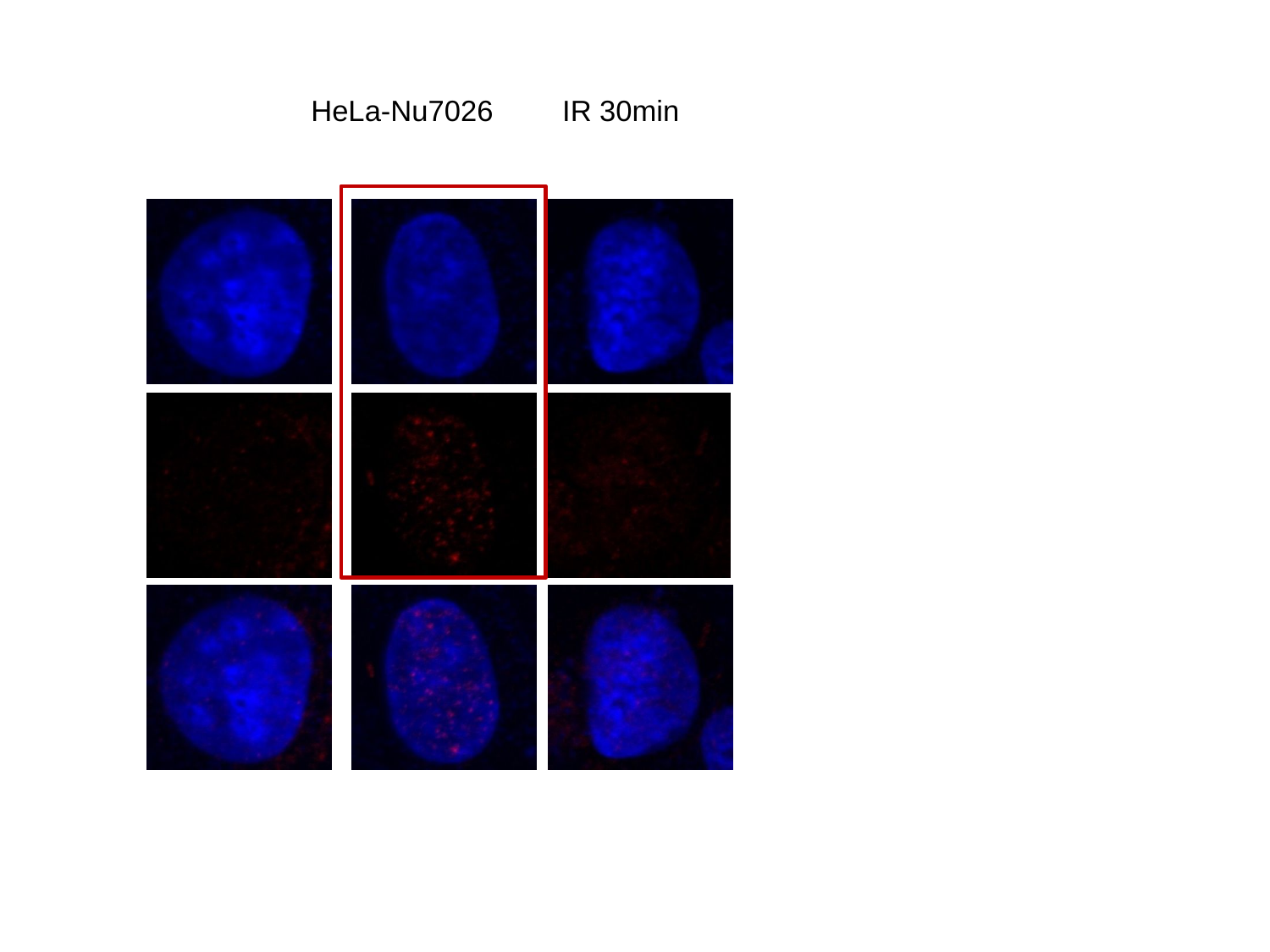

HeLa-Nu7026
IR 30min

## Slide 23
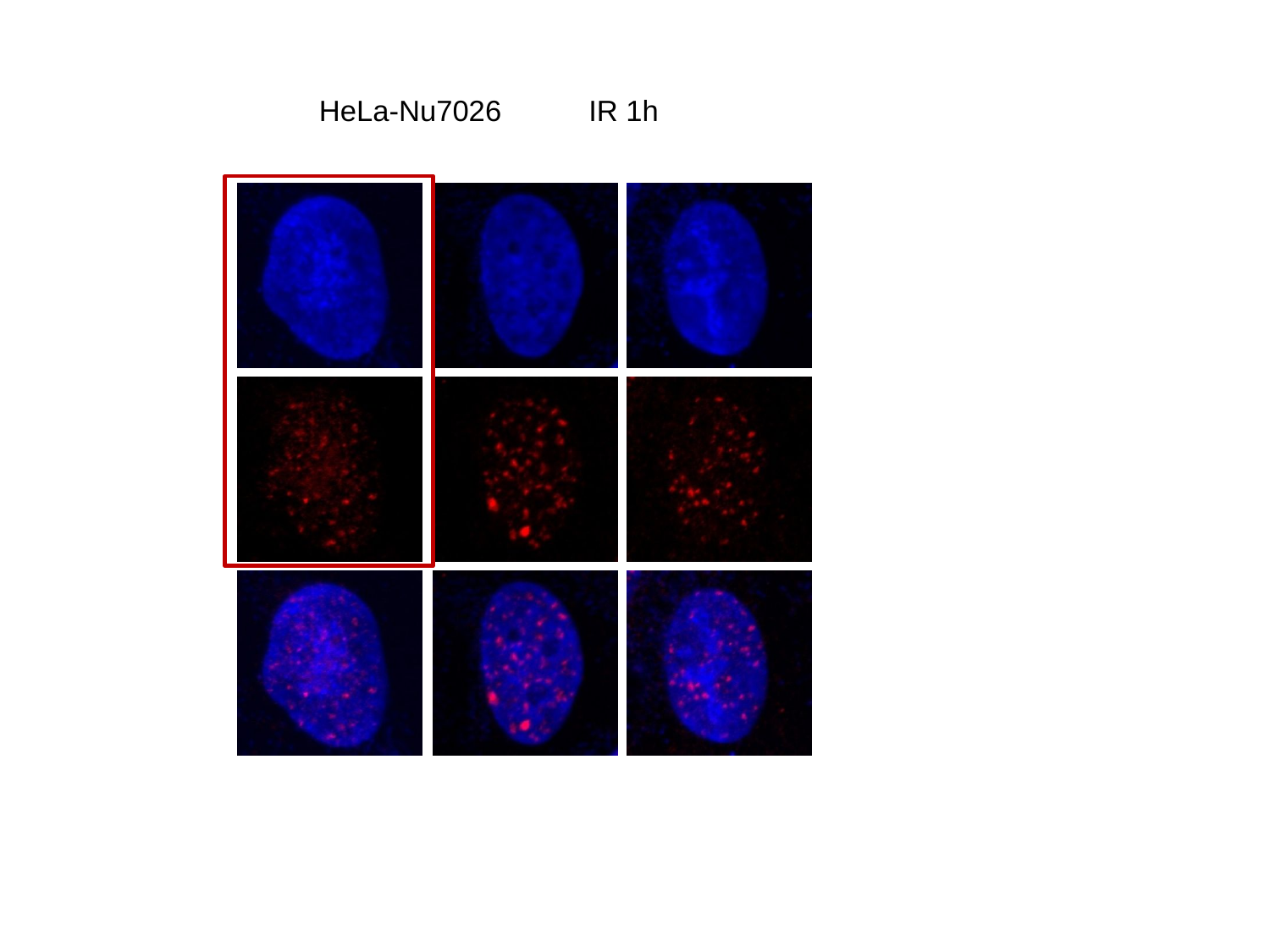

IR 1h
HeLa-Nu7026

## Slide 24
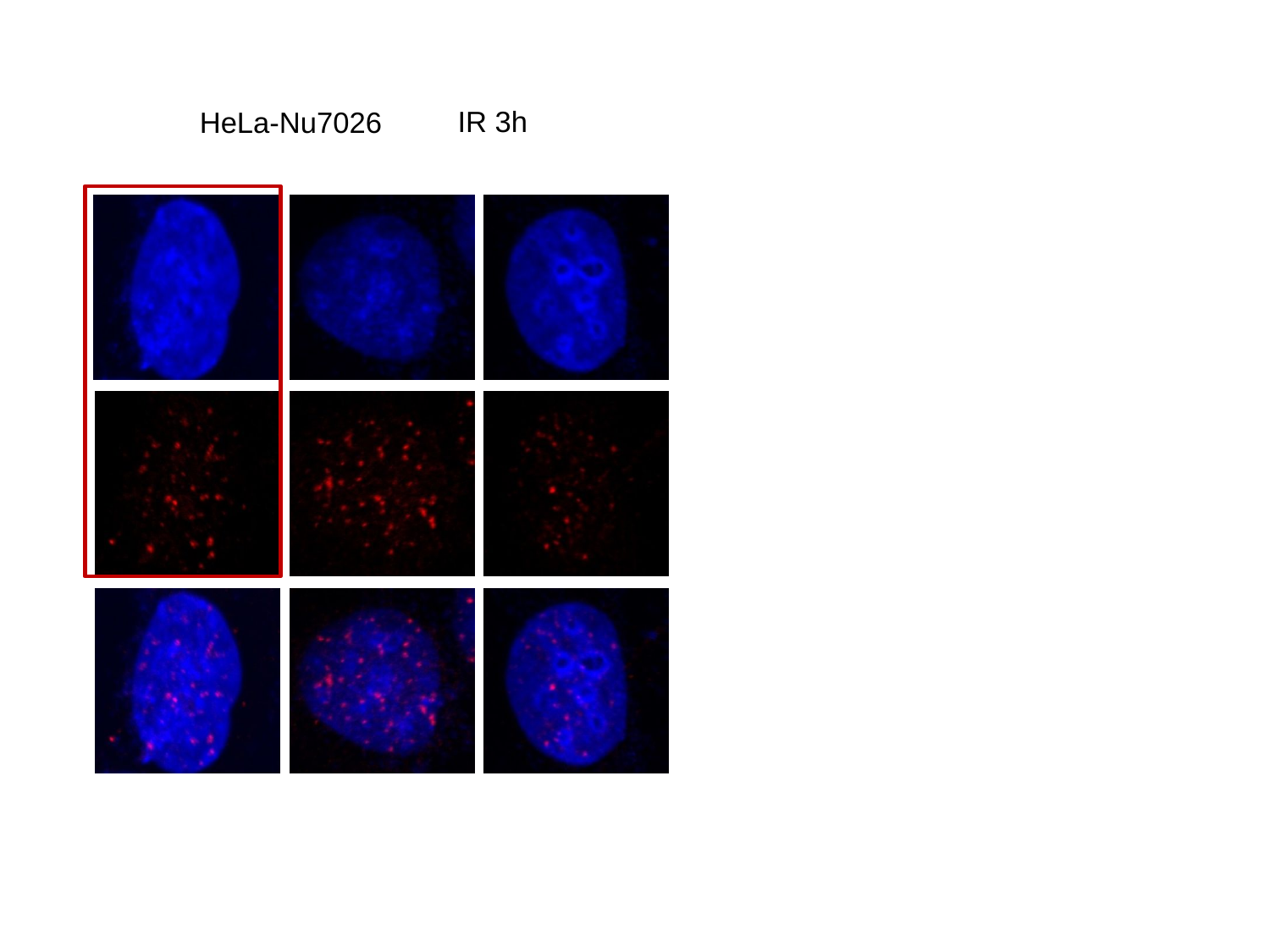

IR 3h
HeLa-Nu7026
