# Supplementary material for: Author Correction: ID3 regulates the MDC1-mediated DNA damage response in order to maintain genome stability
Source: Nat Commun. 2018 Jun 6;9:2284. doi: 10.1038/s41467-018-04599-6 (PMC5989224; doi:10.1038/s41467-018-04599-6)
Supplement: Supplementary file 1 — Supplementary Data 1 [file 41467_2018_4599_MOESM1_ESM.zip › Fig3d/Fig 3d.pptx]

## Slide 1
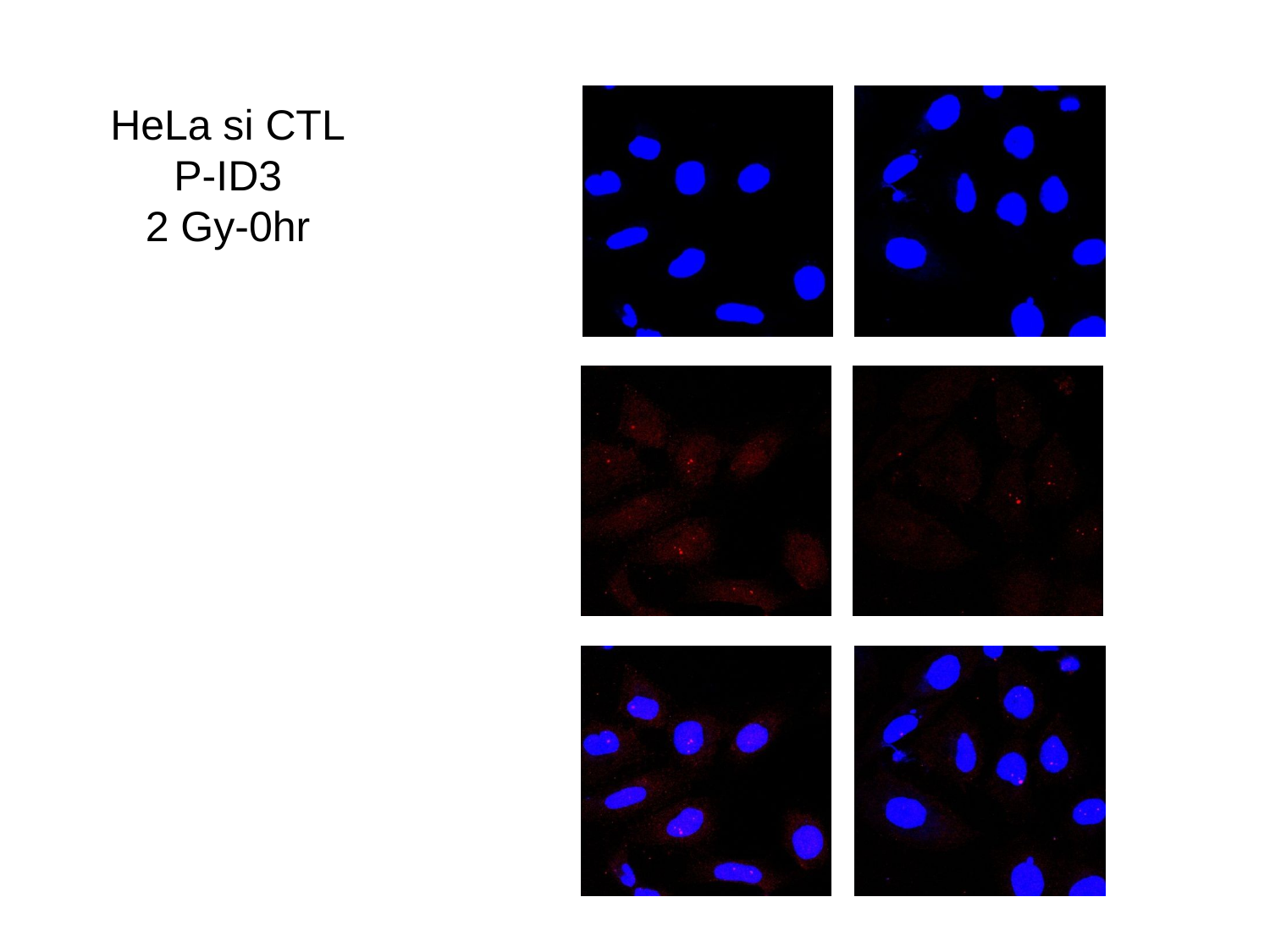

HeLa si CTL
P-ID3
2 Gy-0hr

## Slide 2
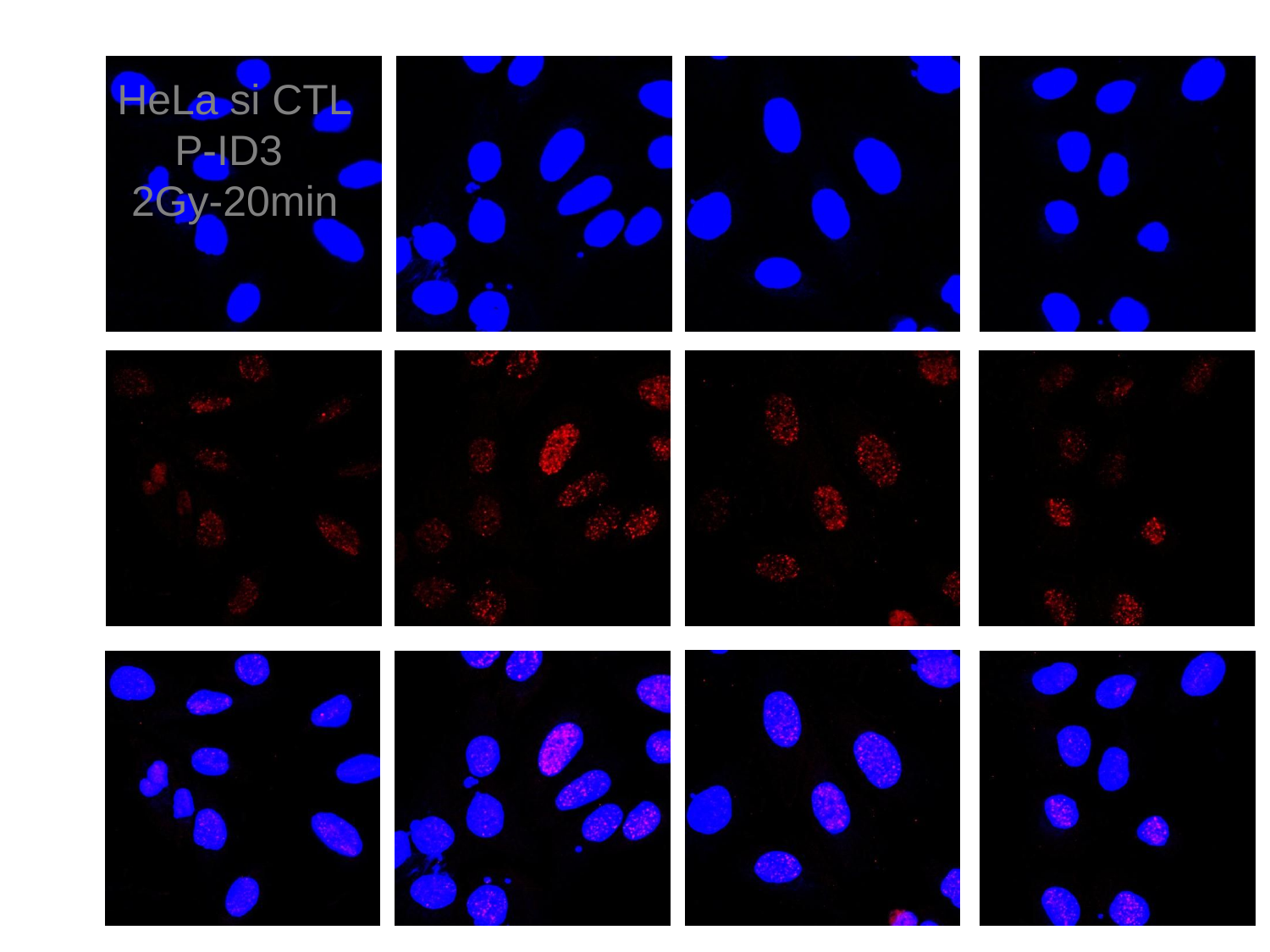

HeLa si CTL
P-ID3
2Gy-20min

## Slide 3
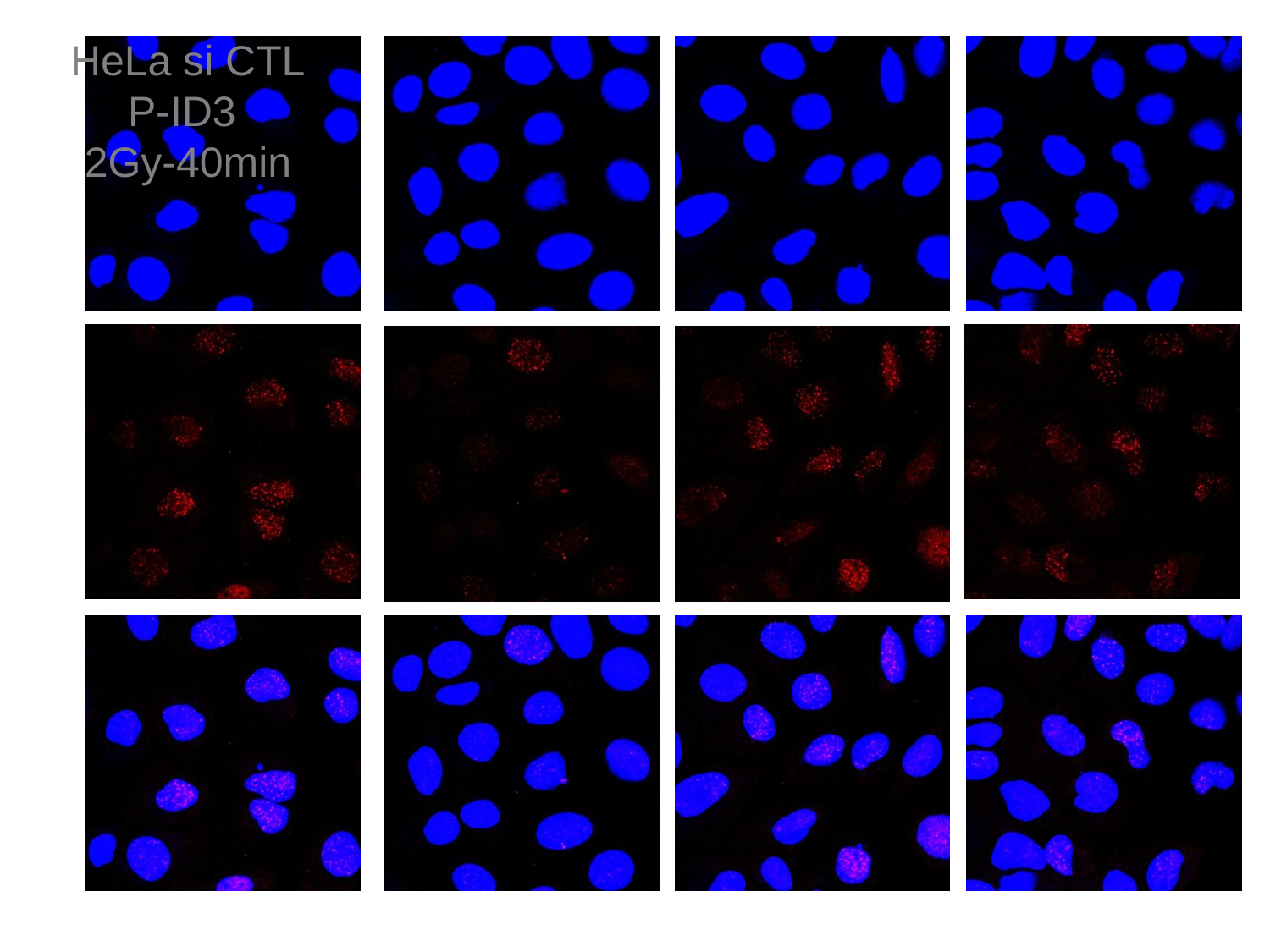

HeLa si CTL
P-ID3
2Gy-40min

## Slide 4
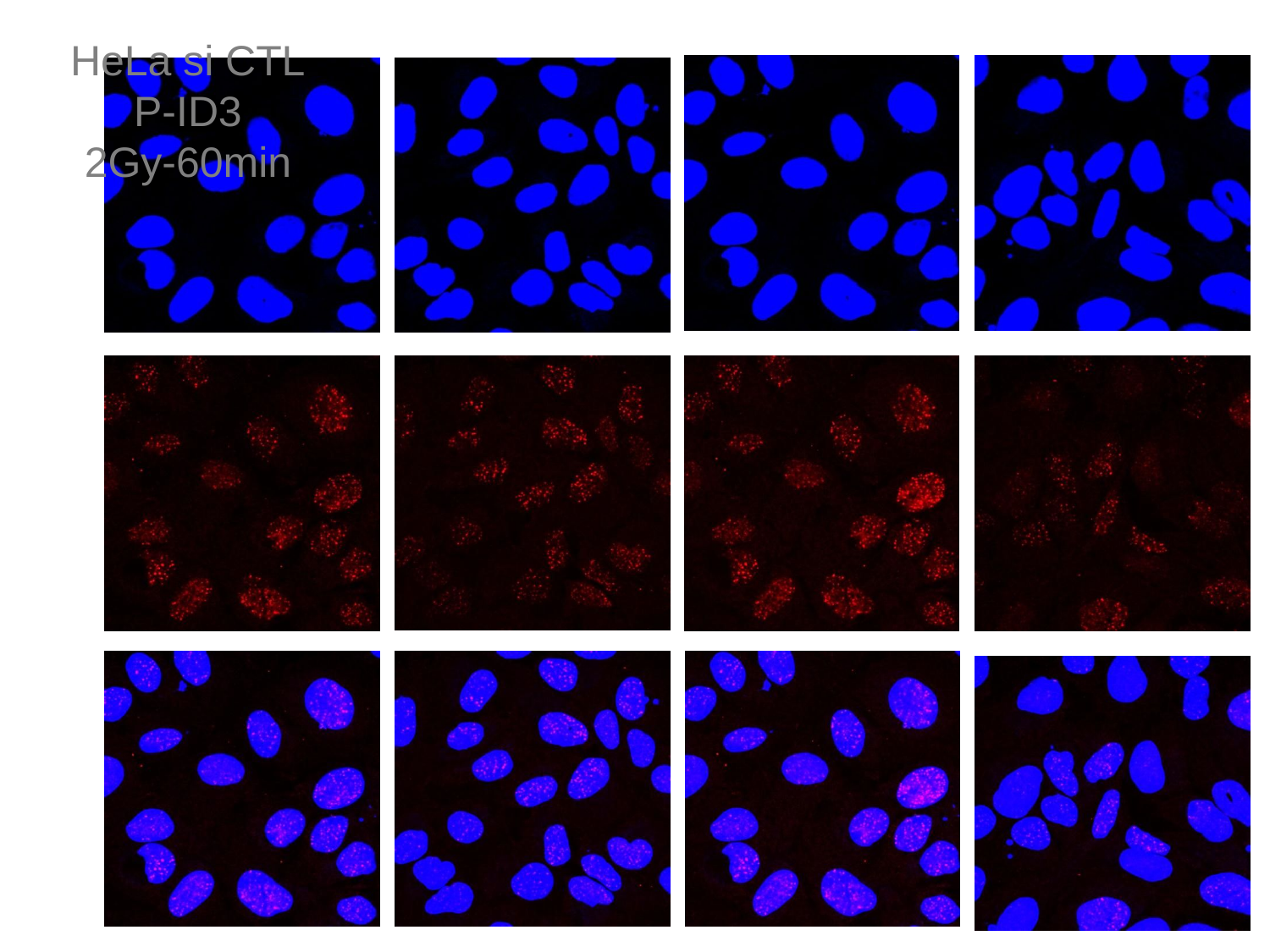

HeLa si CTL
P-ID3
2Gy-60min

## Slide 5
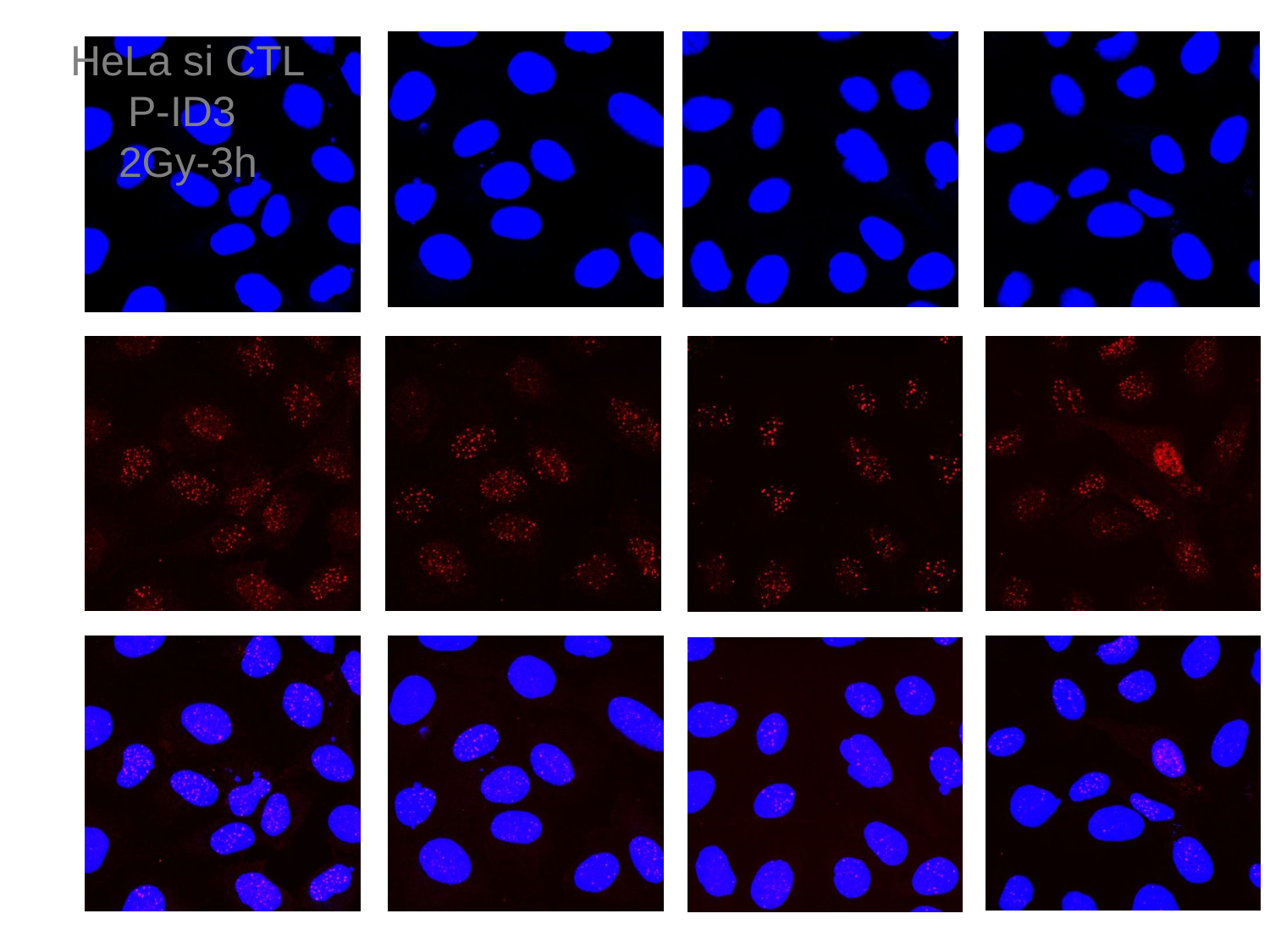

HeLa si CTL
P-ID3
2Gy-3h

## Slide 6
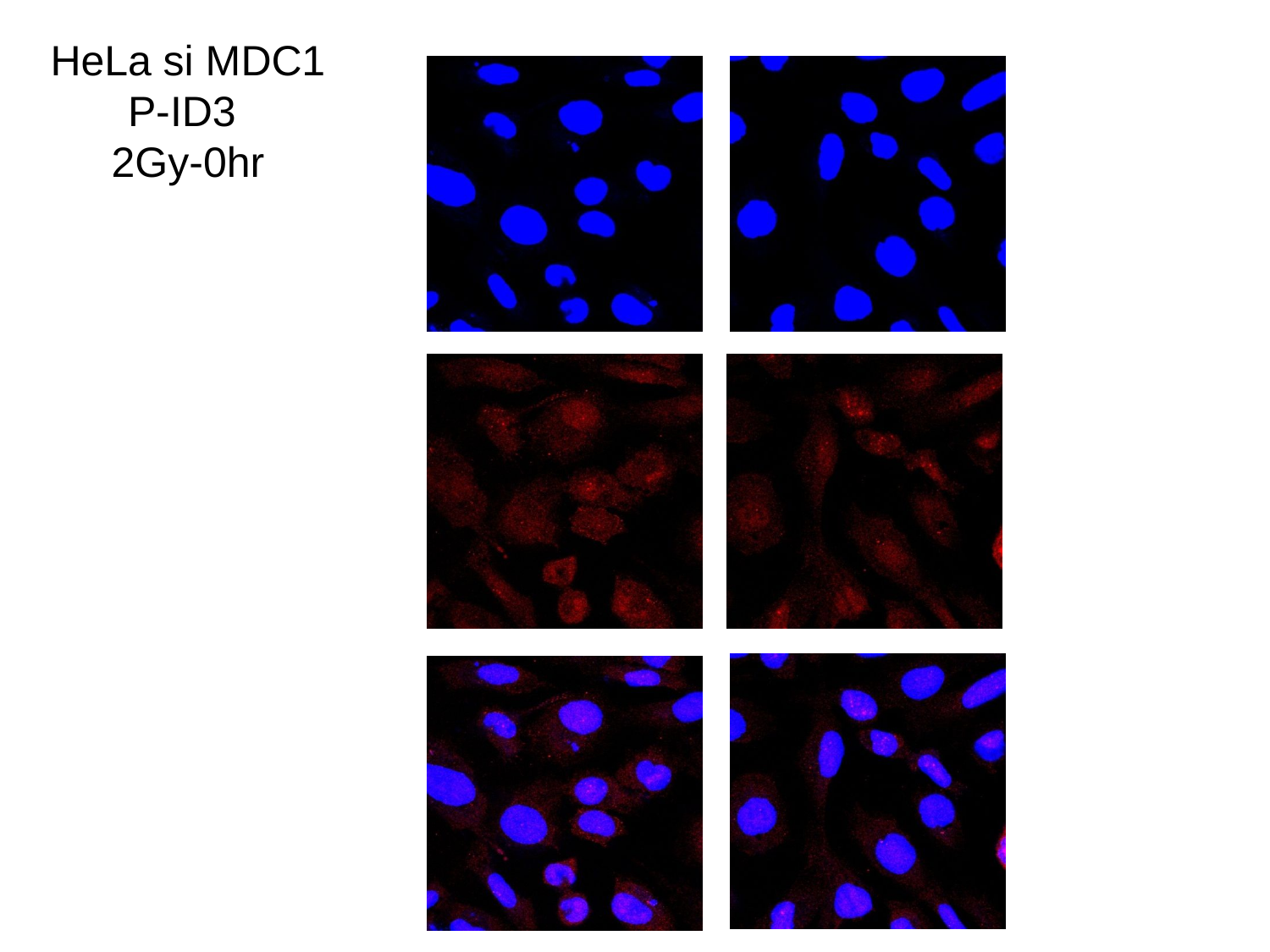

HeLa si MDC1
P-ID3
2Gy-0hr

## Slide 7
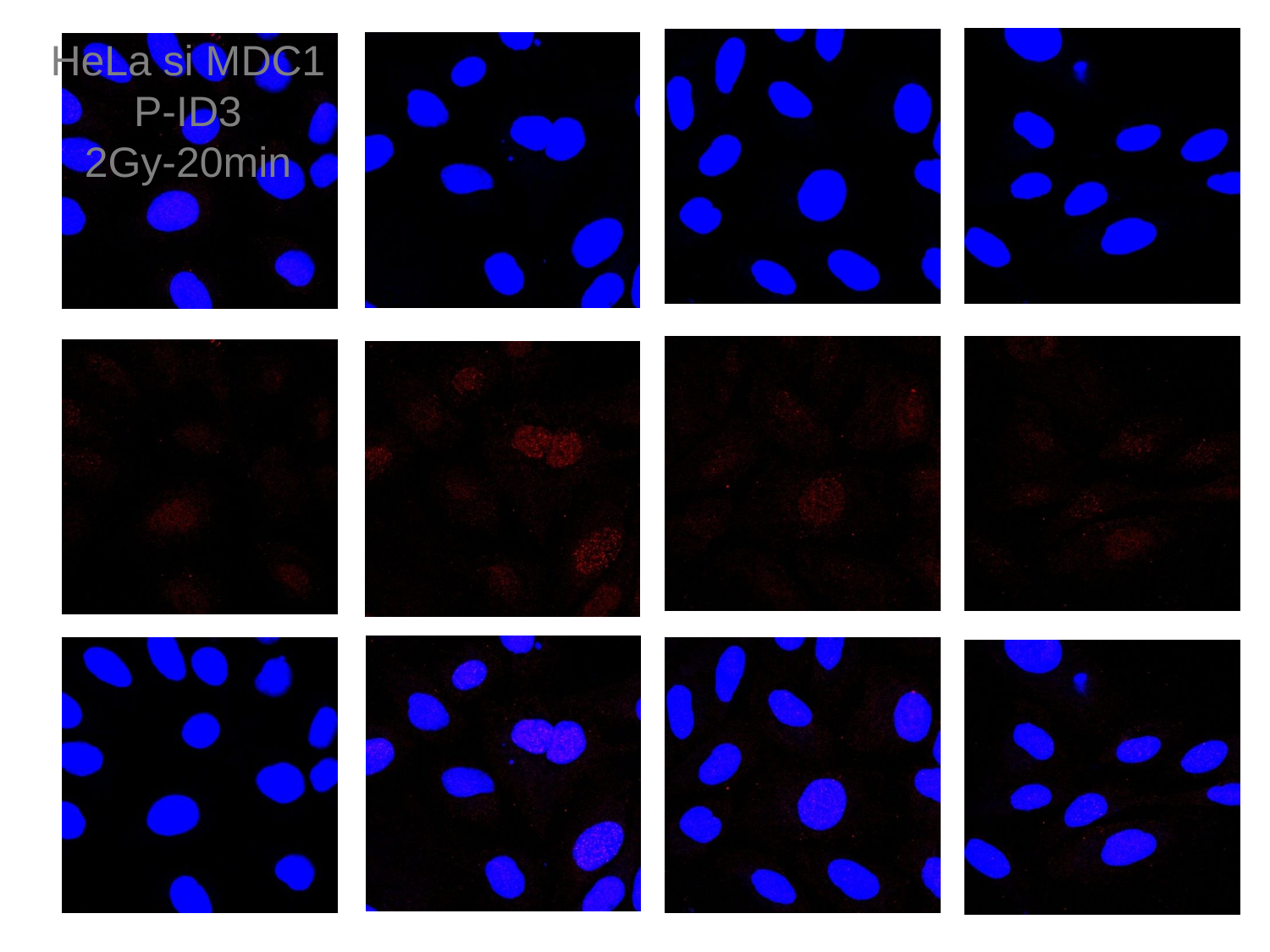

HeLa si MDC1
P-ID3
2Gy-20min

## Slide 8
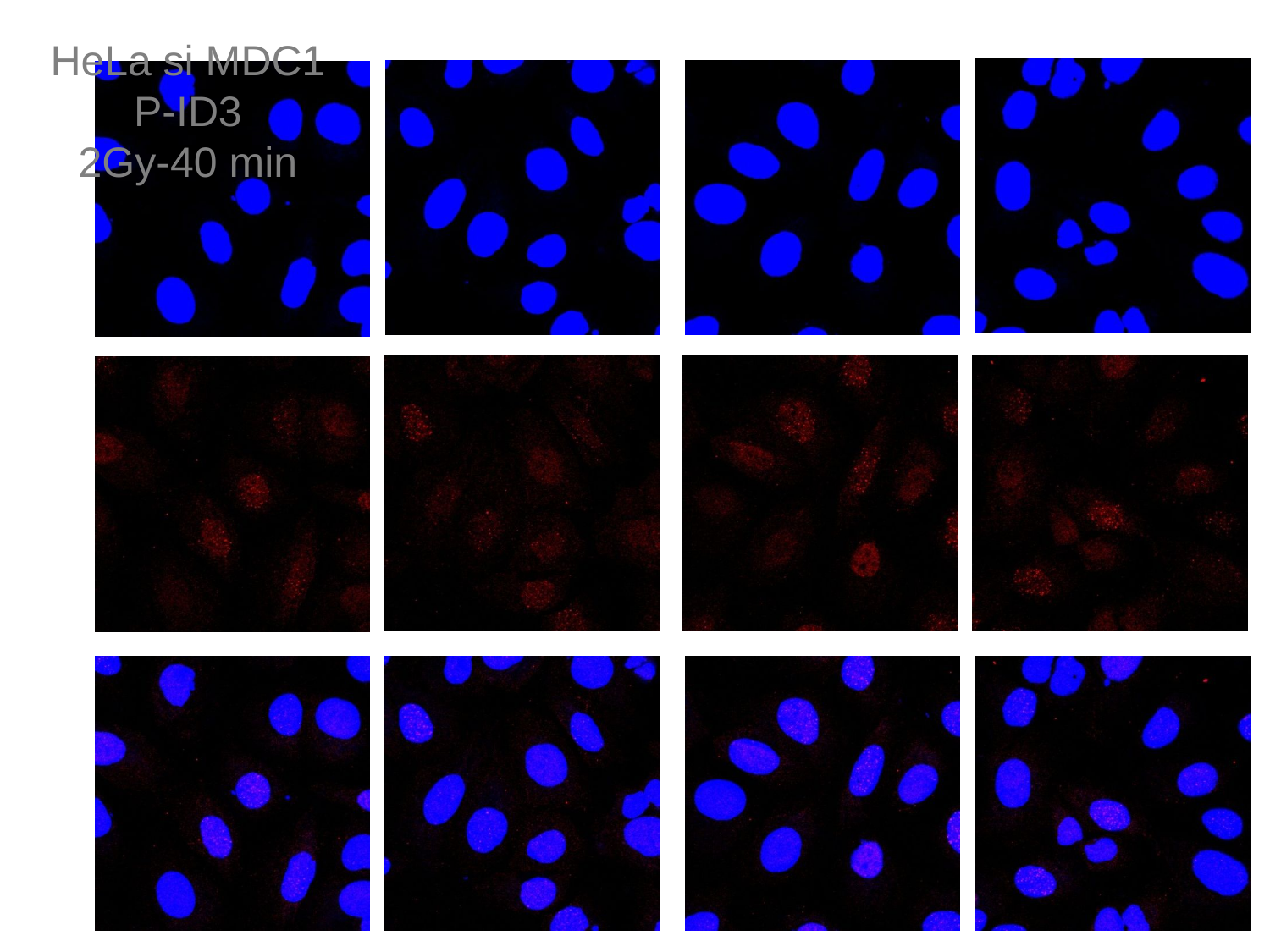

HeLa si MDC1
P-ID3
2Gy-40 min

## Slide 9
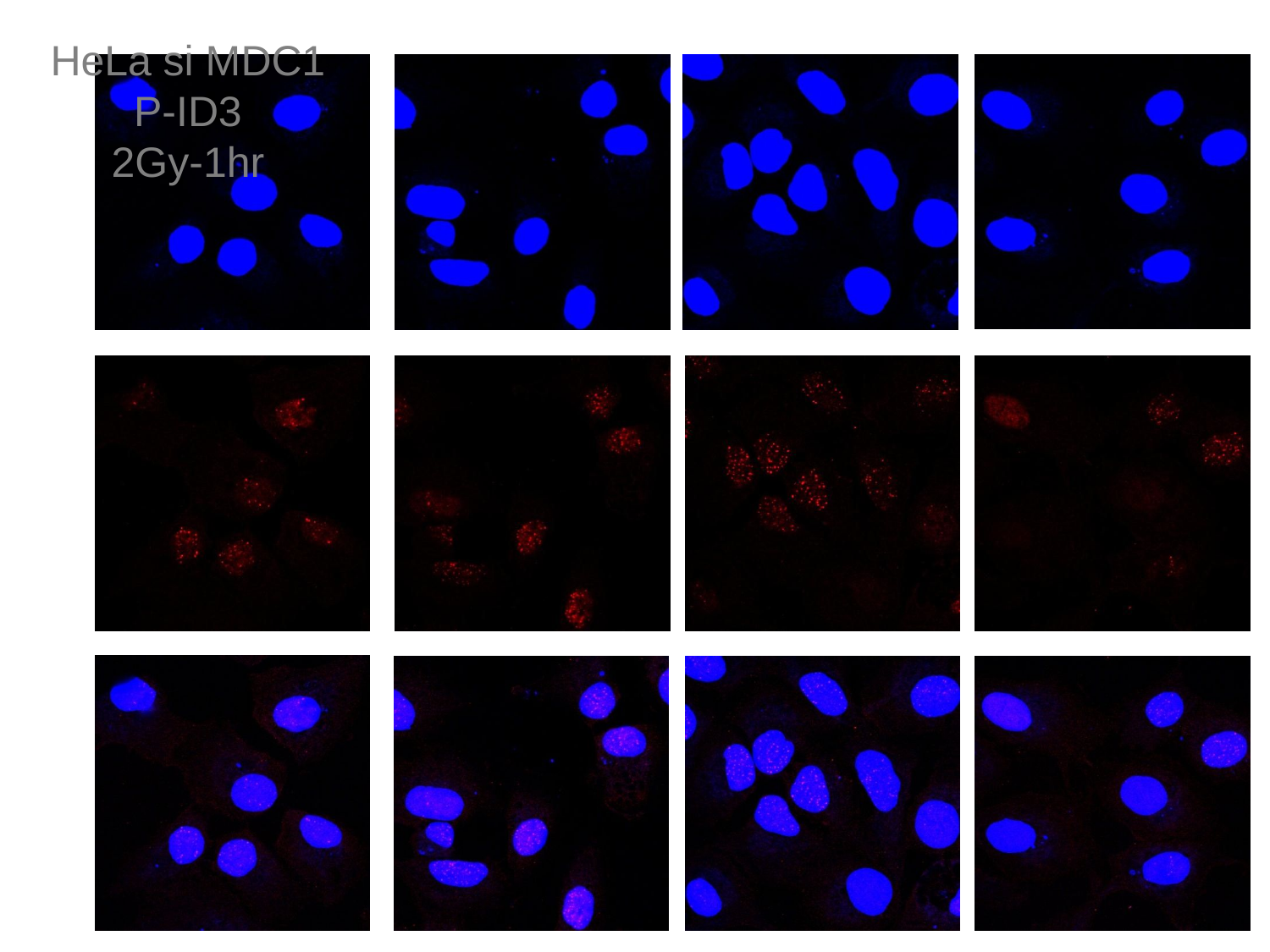

HeLa si MDC1
P-ID3
2Gy-1hr

## Slide 10
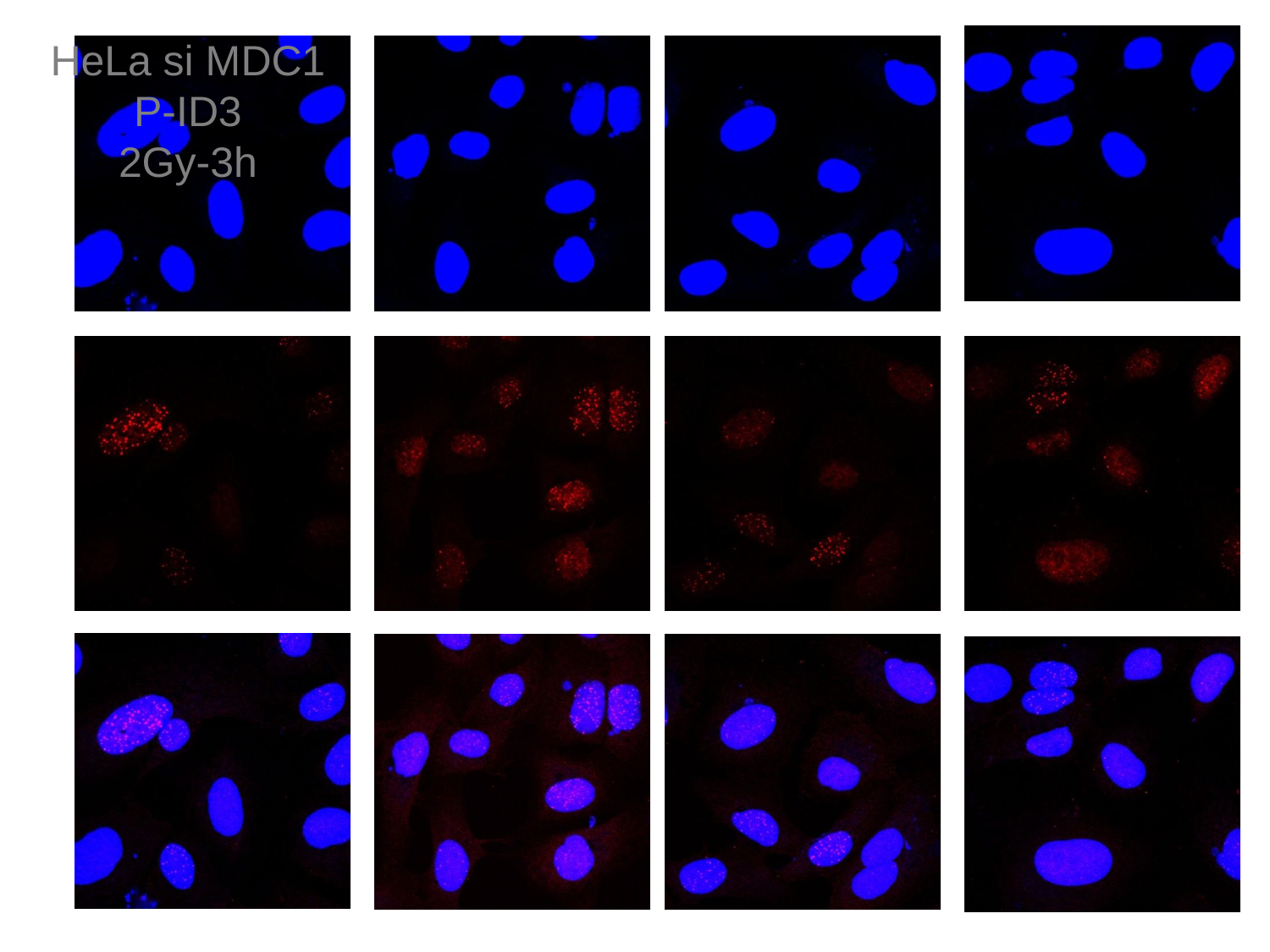

HeLa si MDC1
P-ID3
2Gy-3h

## Slide 11
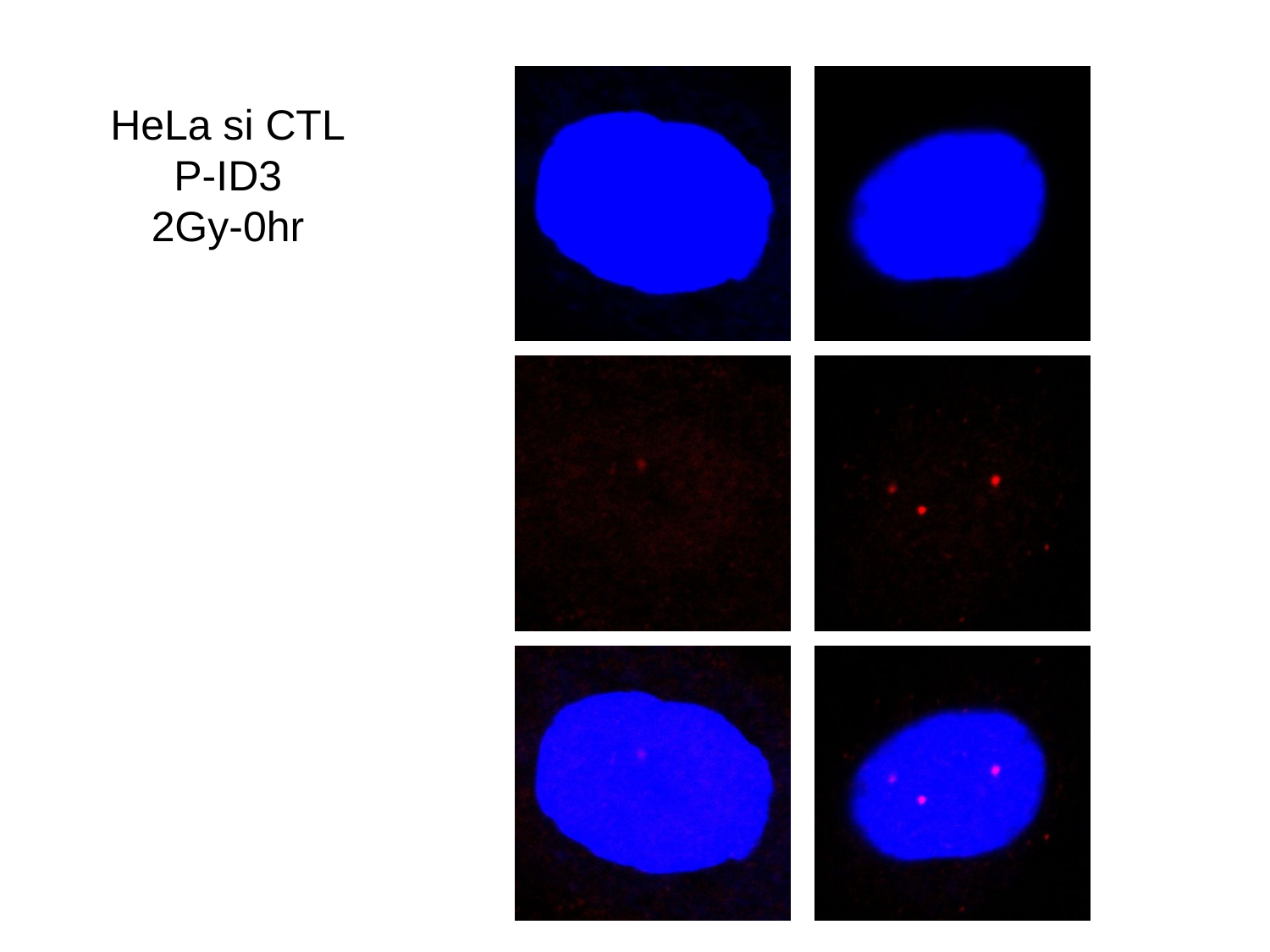

HeLa si CTL
P-ID3
2Gy-0hr

## Slide 12
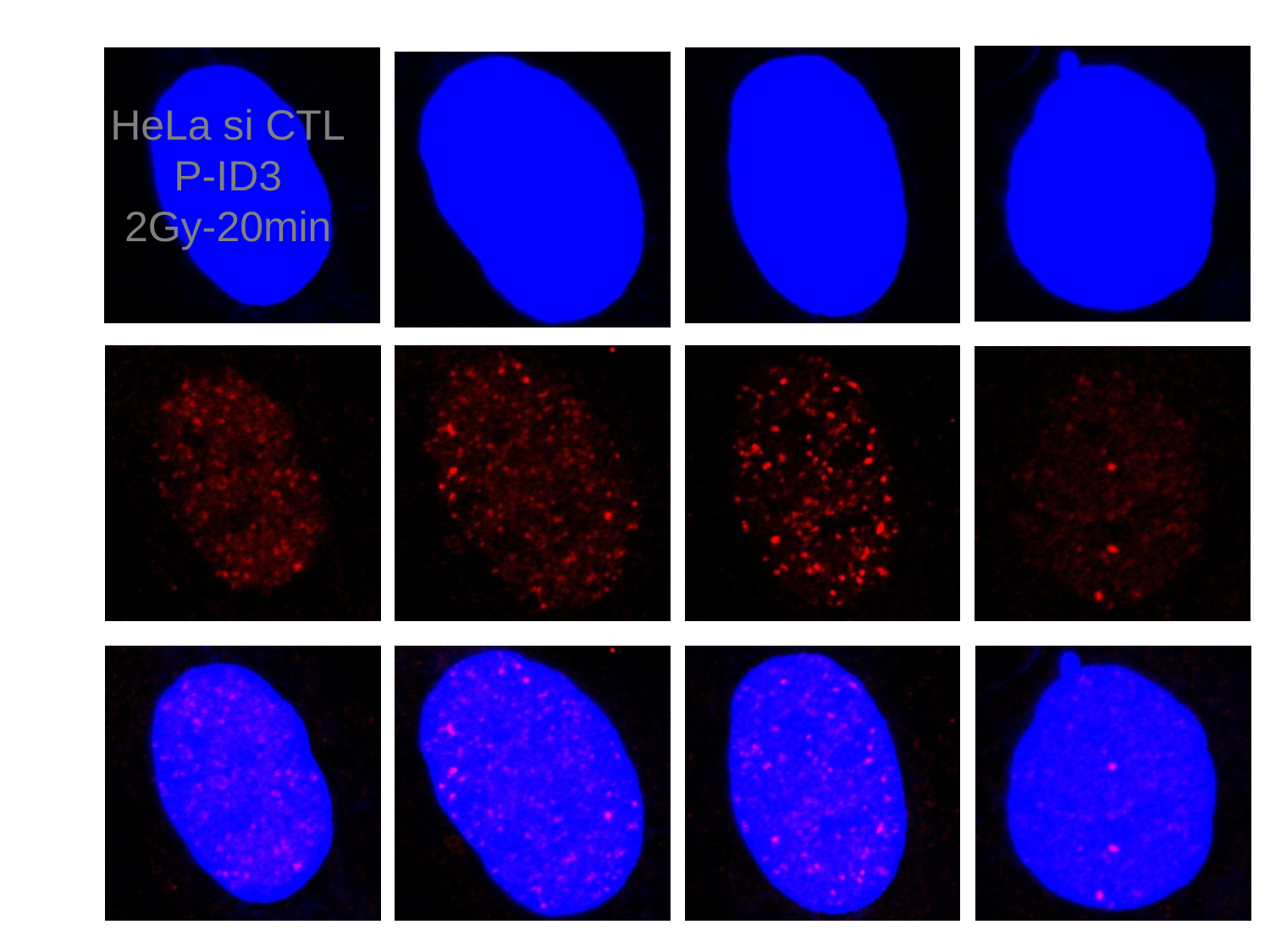

HeLa si CTL
P-ID3
2Gy-20min

## Slide 13
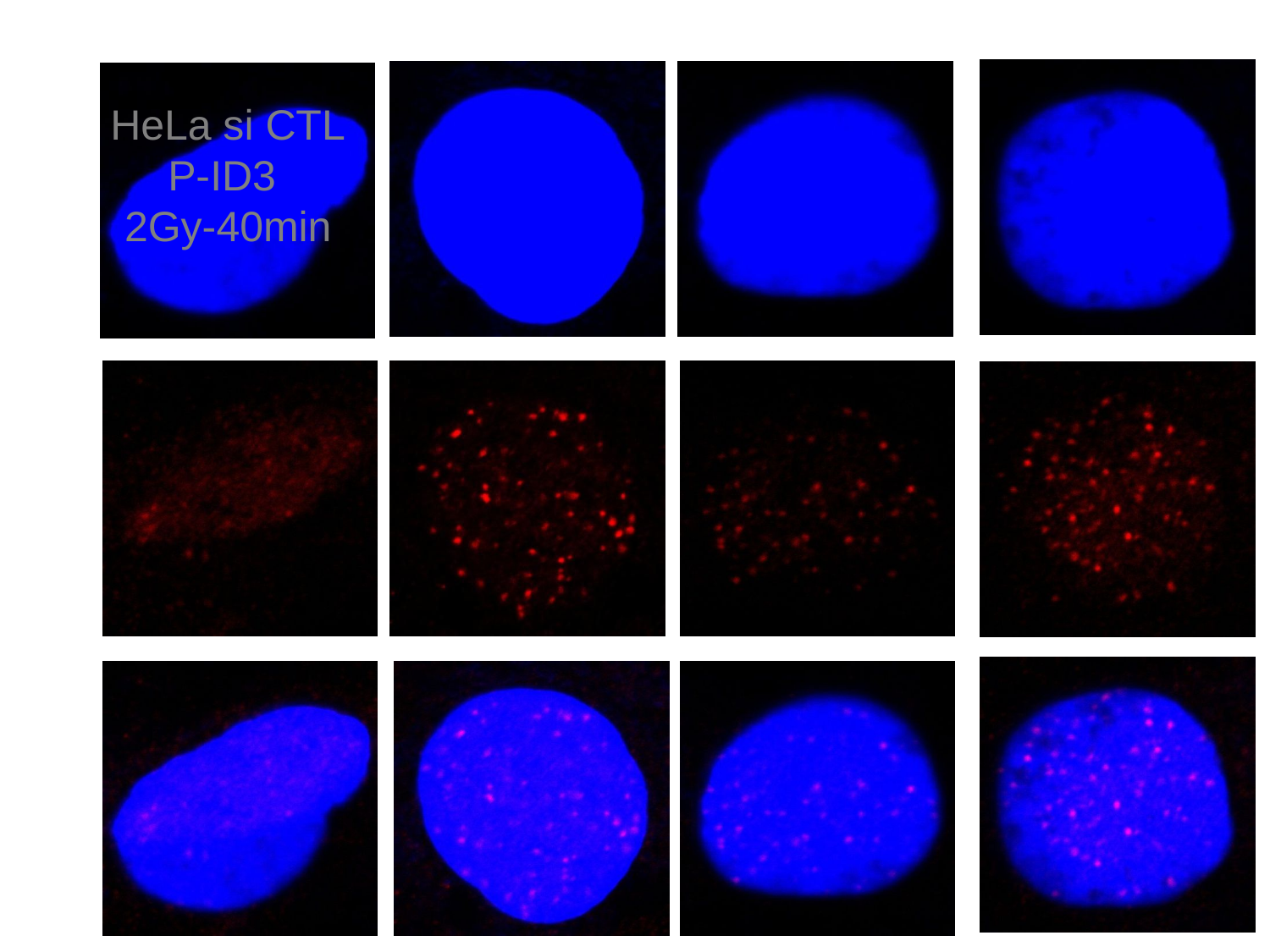

HeLa si CTL
P-ID3
2Gy-40min

## Slide 14
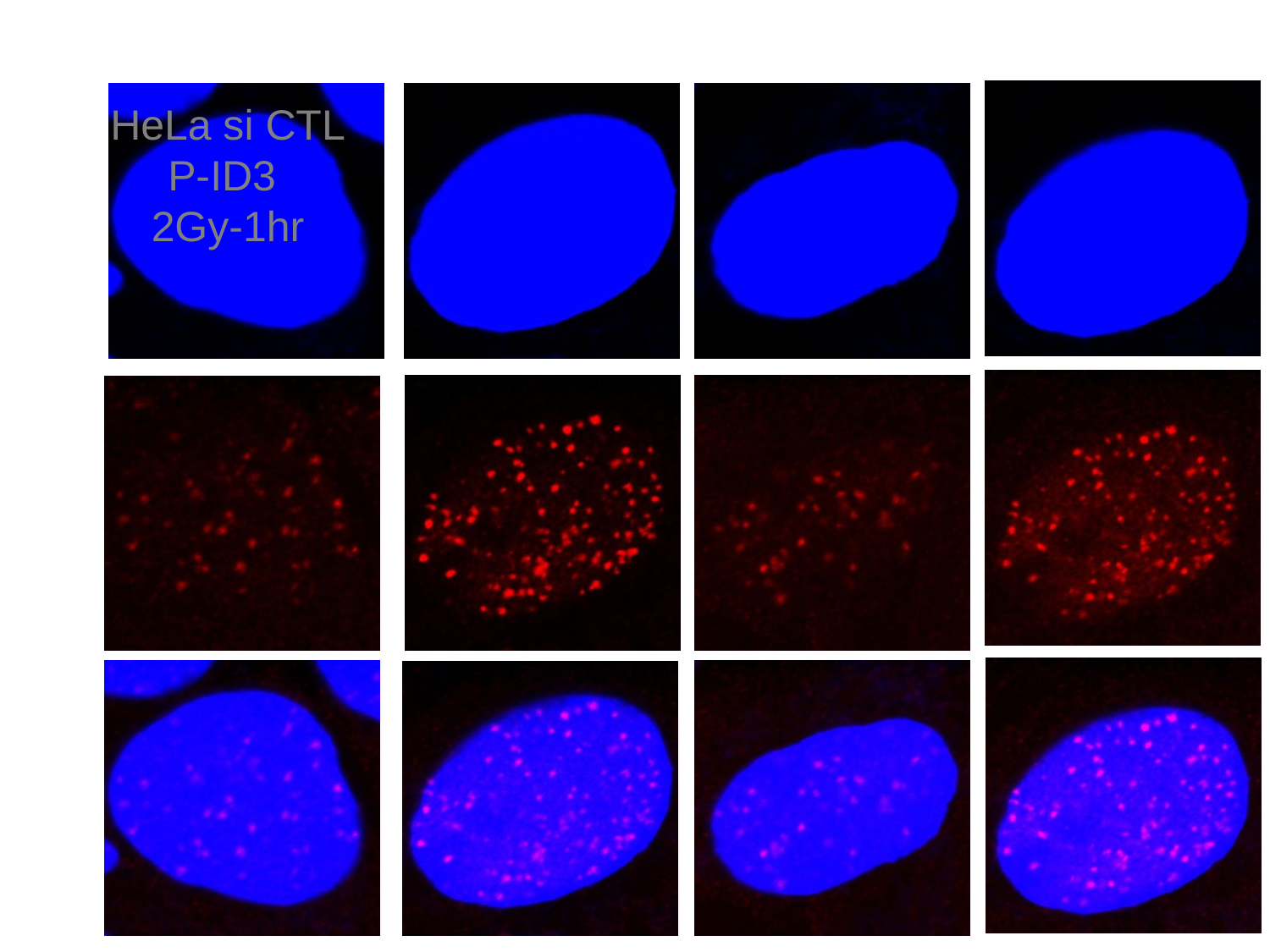

HeLa si CTL
P-ID3
2Gy-1hr

## Slide 15
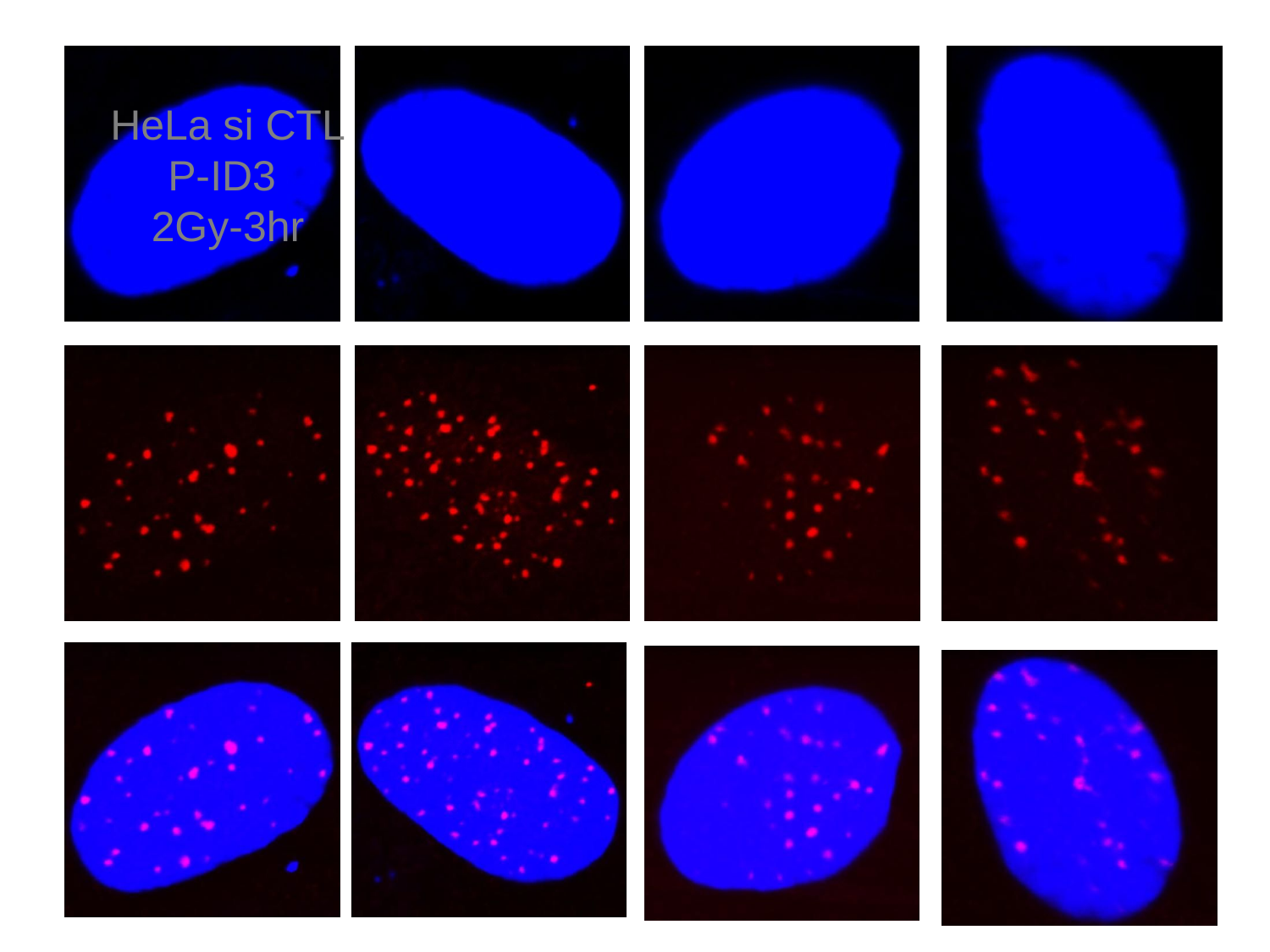

HeLa si CTL
P-ID3
2Gy-3hr

## Slide 16
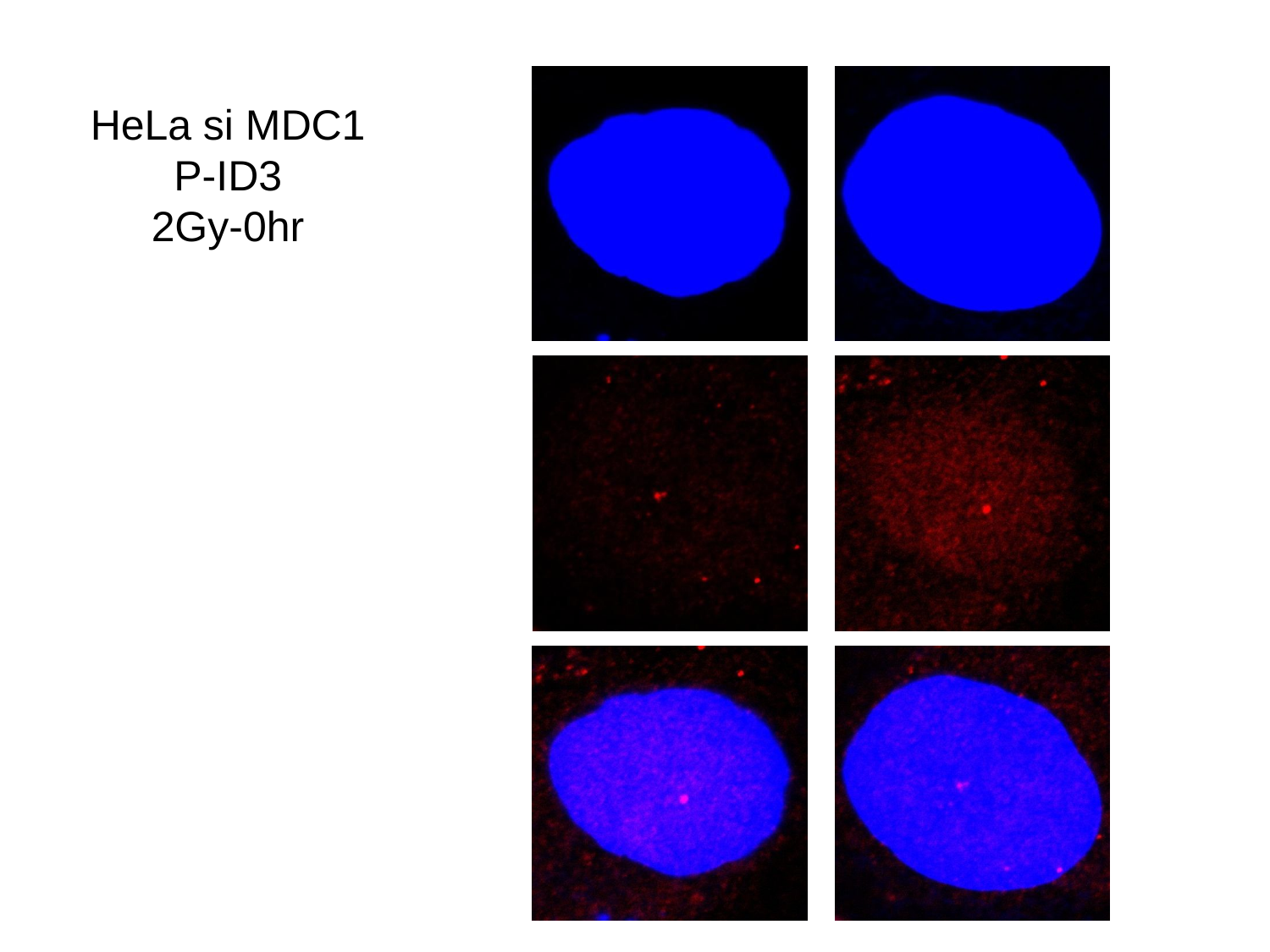

HeLa si MDC1
P-ID3
2Gy-0hr

## Slide 17
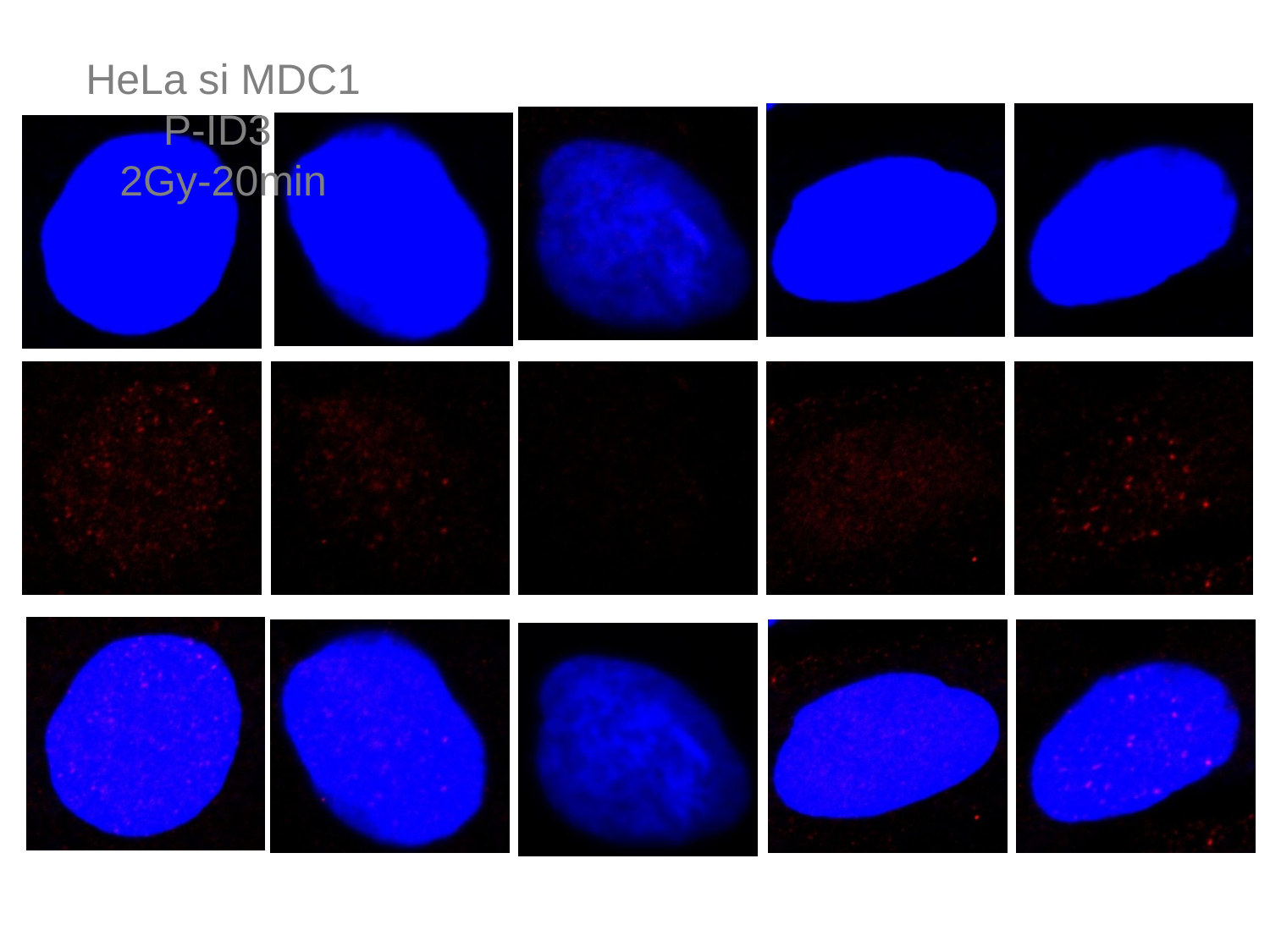

HeLa si MDC1
P-ID3
2Gy-20min

## Slide 18
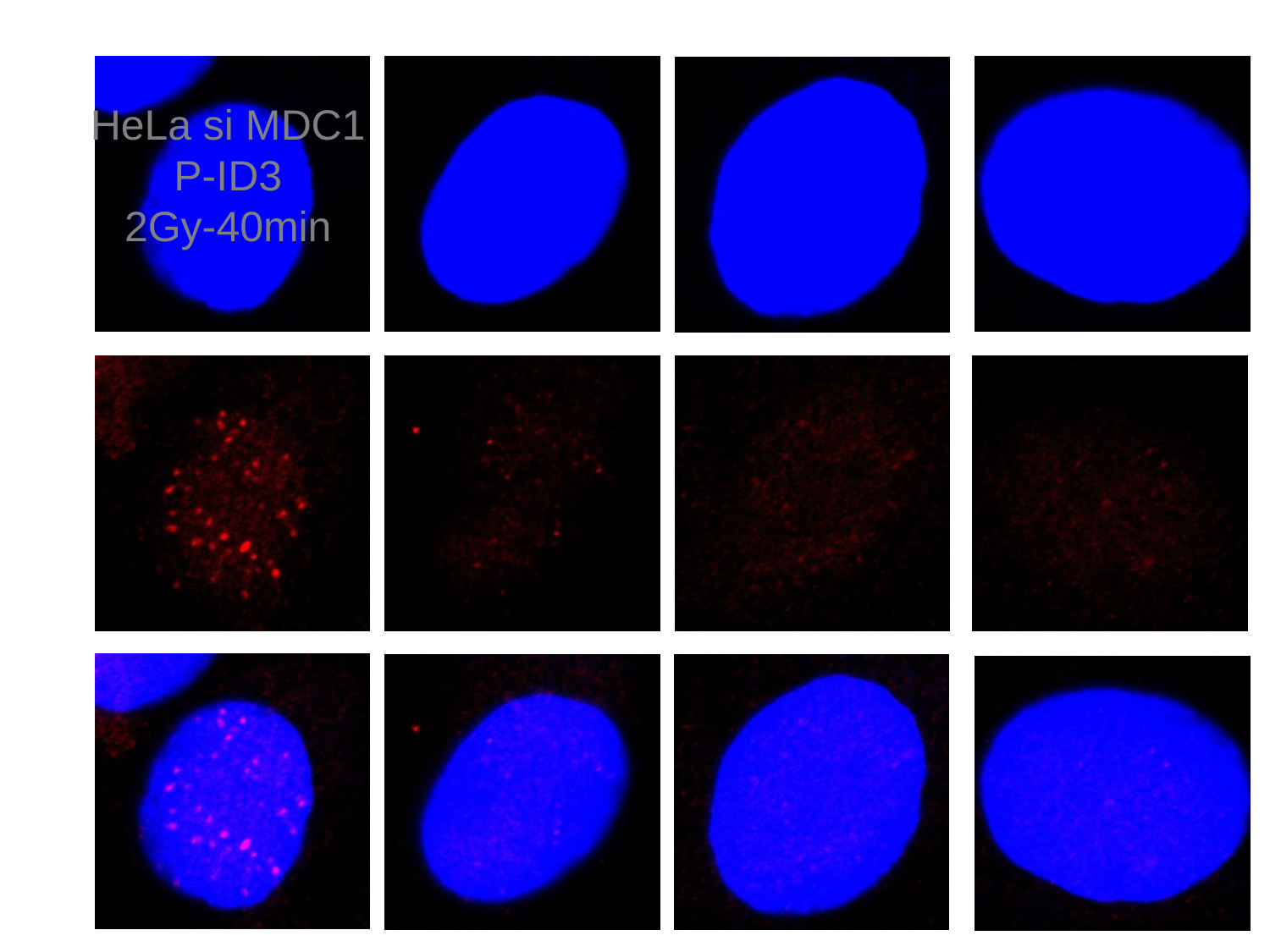

HeLa si MDC1
P-ID3
2Gy-40min

## Slide 19
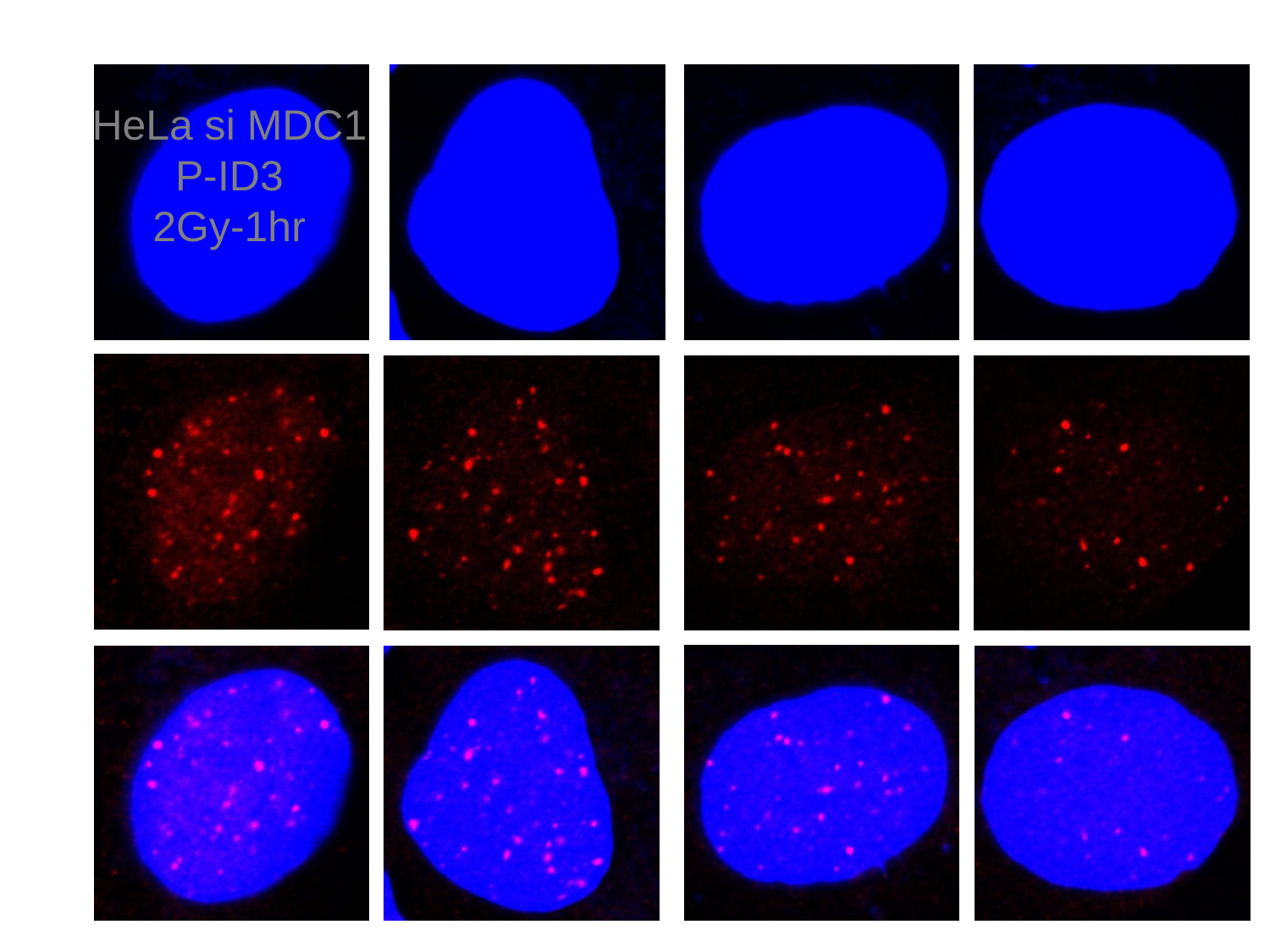

HeLa si MDC1
P-ID3
2Gy-1hr

## Slide 20
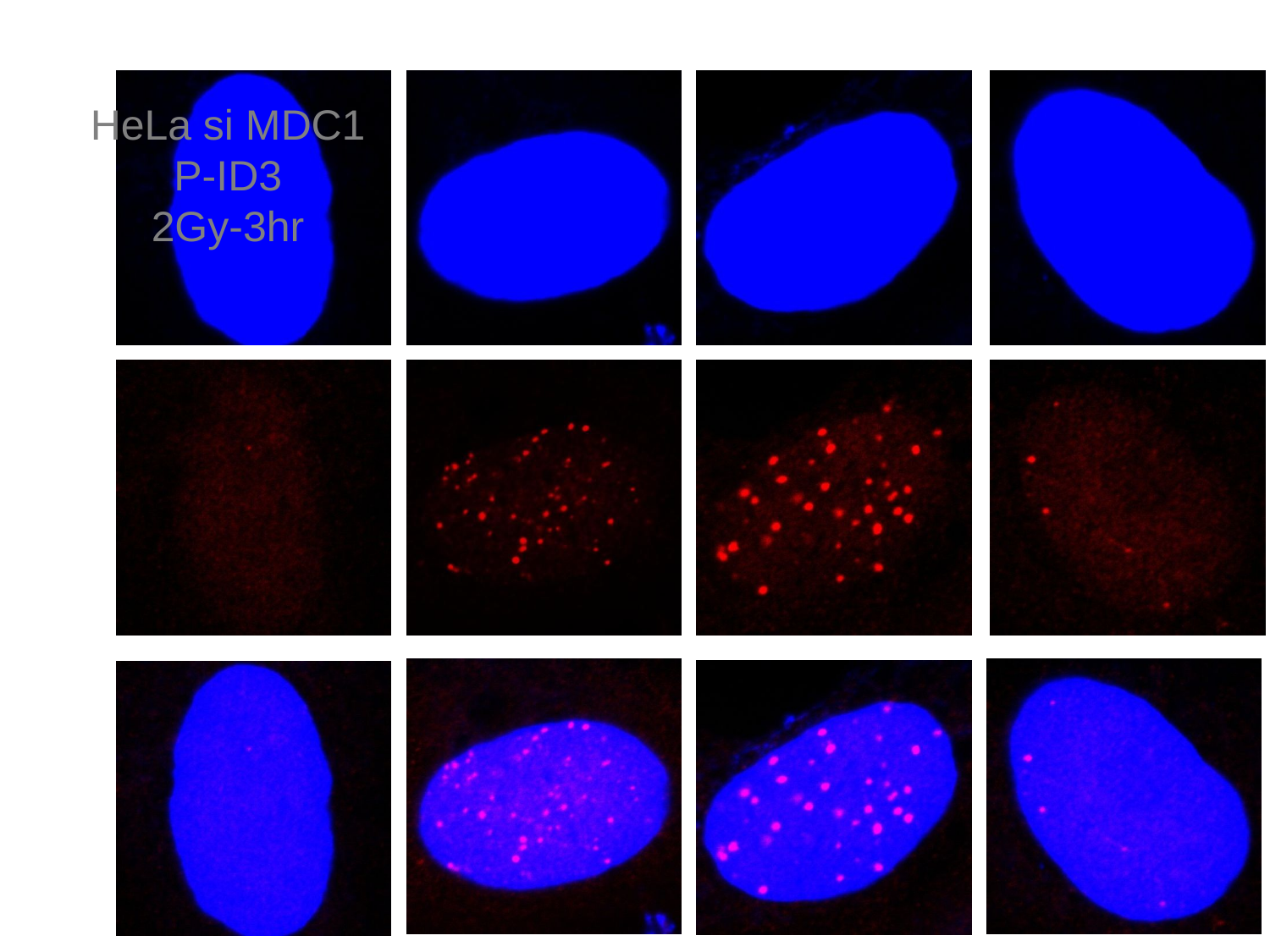

HeLa si MDC1
P-ID3
2Gy-3hr

## Slide 21
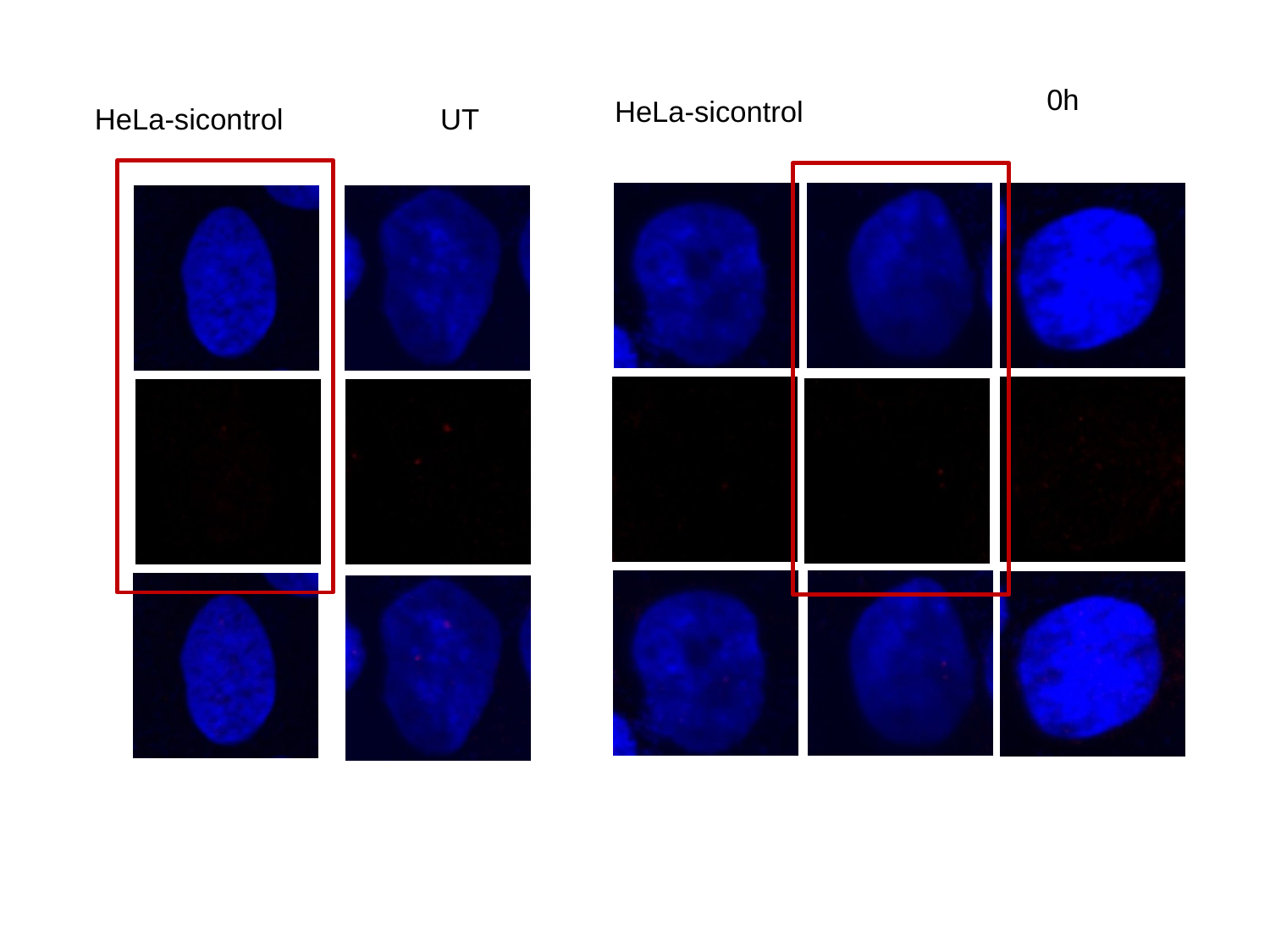

0h
HeLa-sicontrol
HeLa-sicontrol
UT

## Slide 22
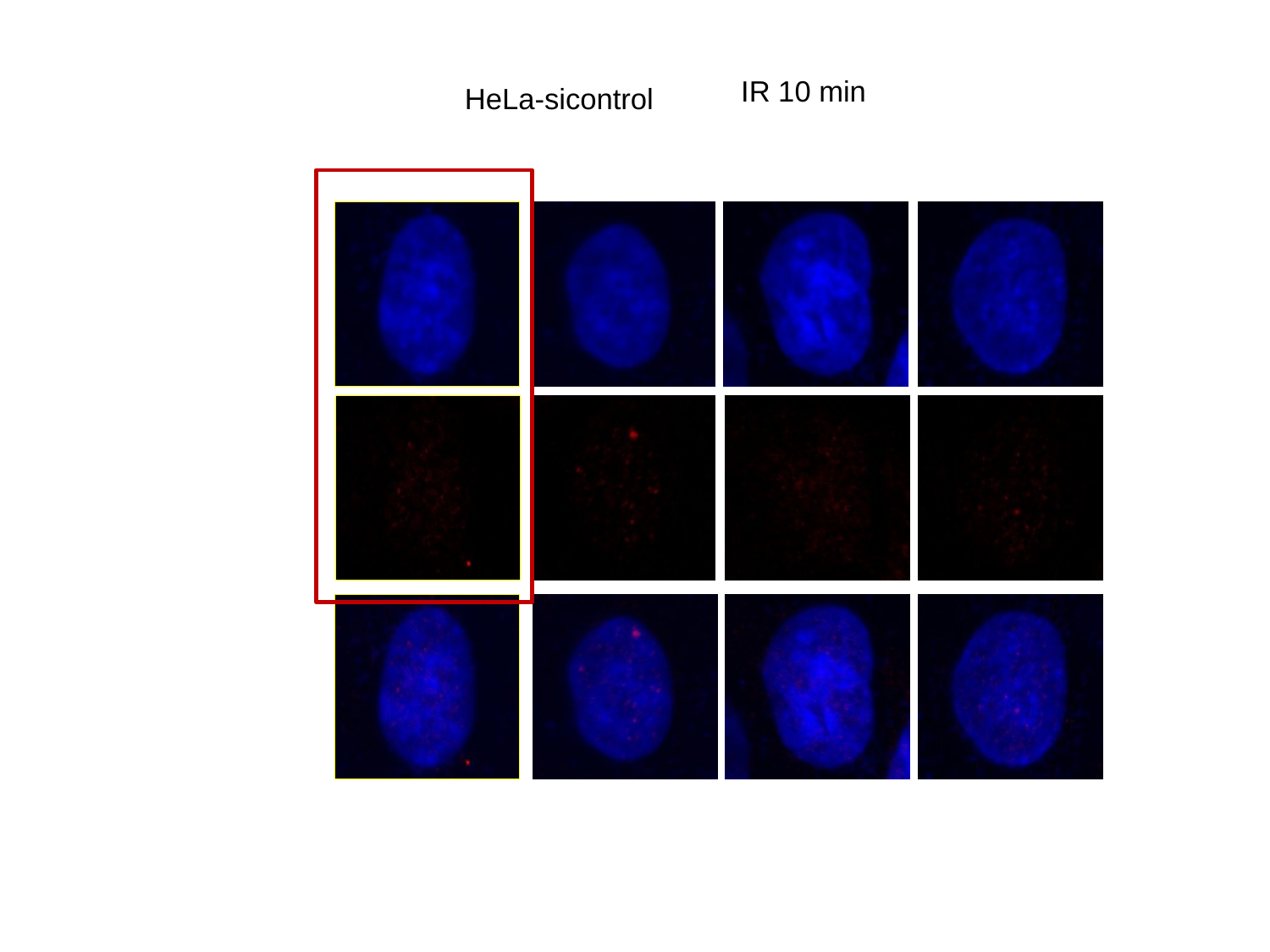

IR 10 min
HeLa-sicontrol

## Slide 23
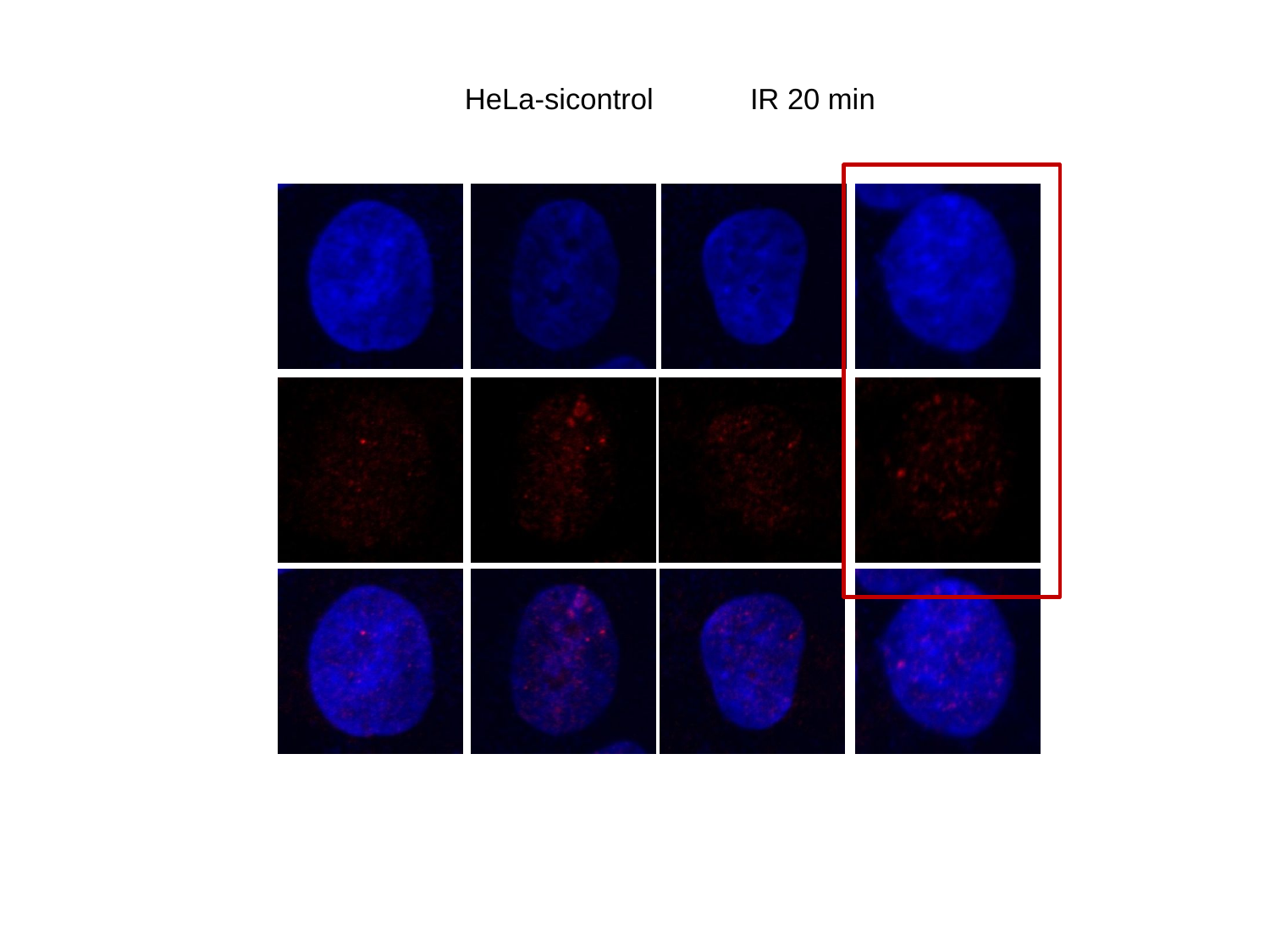

IR 20 min
HeLa-sicontrol

## Slide 24
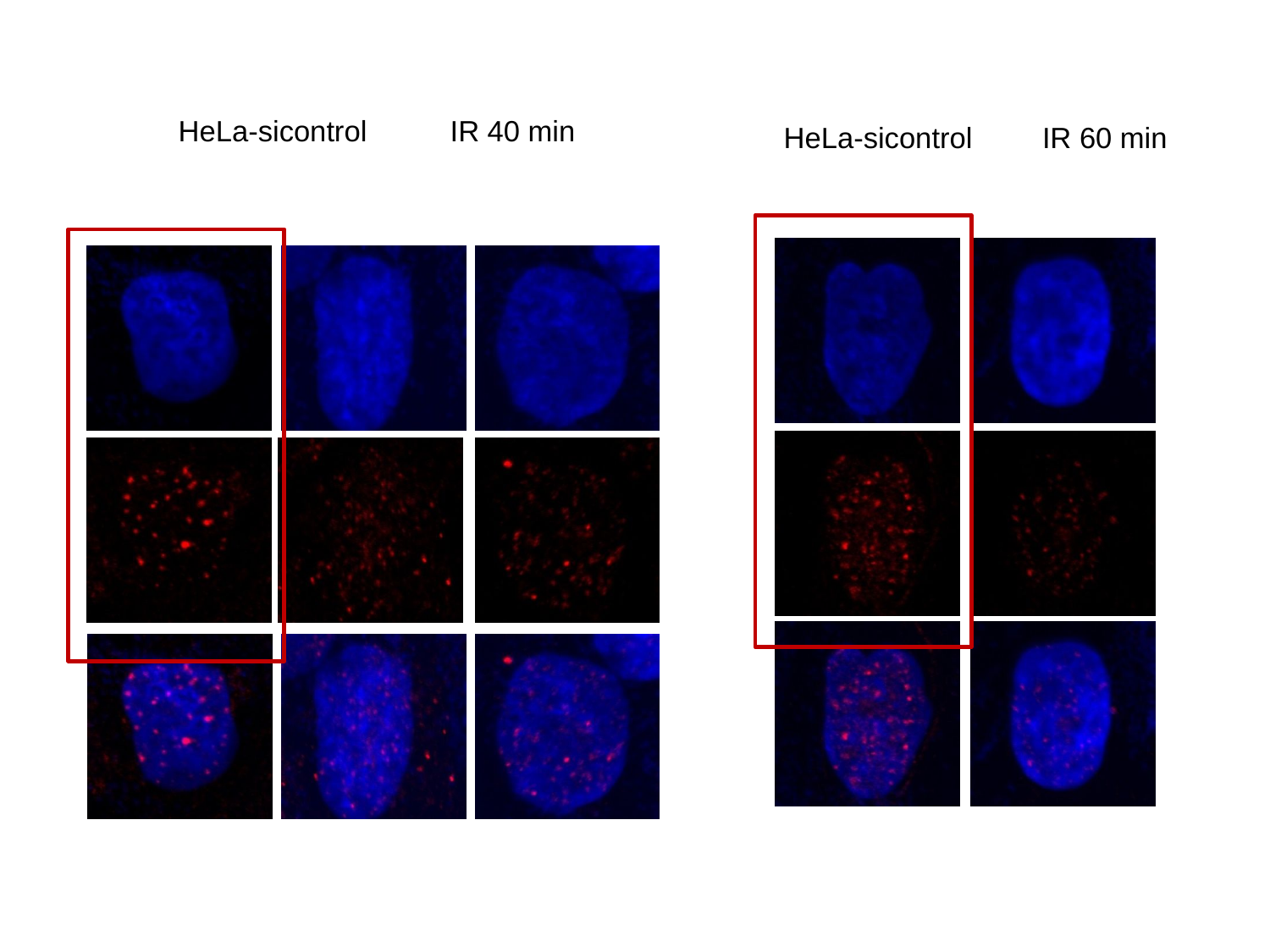

HeLa-sicontrol
IR 40 min
HeLa-sicontrol
IR 60 min

## Slide 25
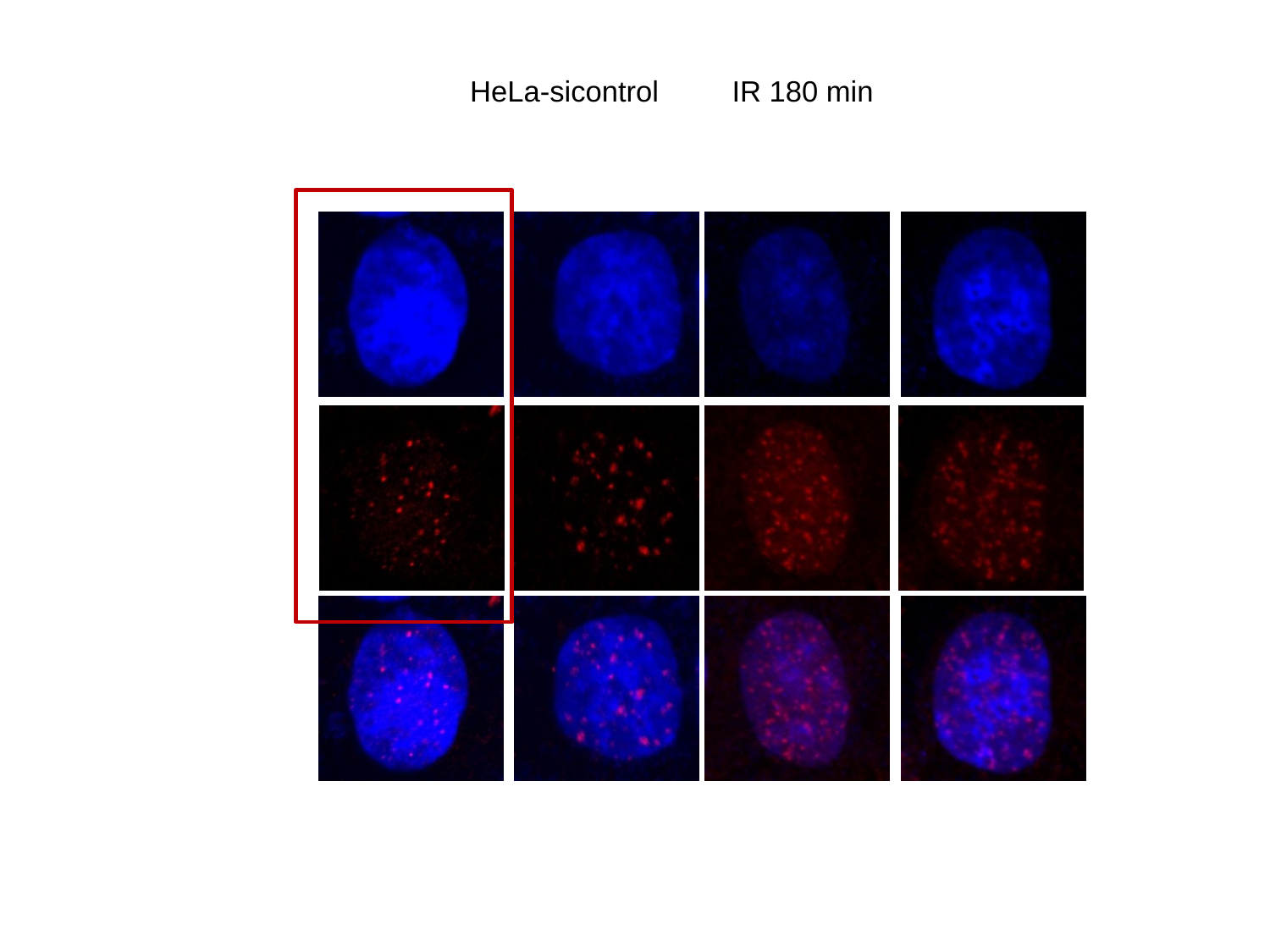

HeLa-sicontrol
IR 180 min

## Slide 26
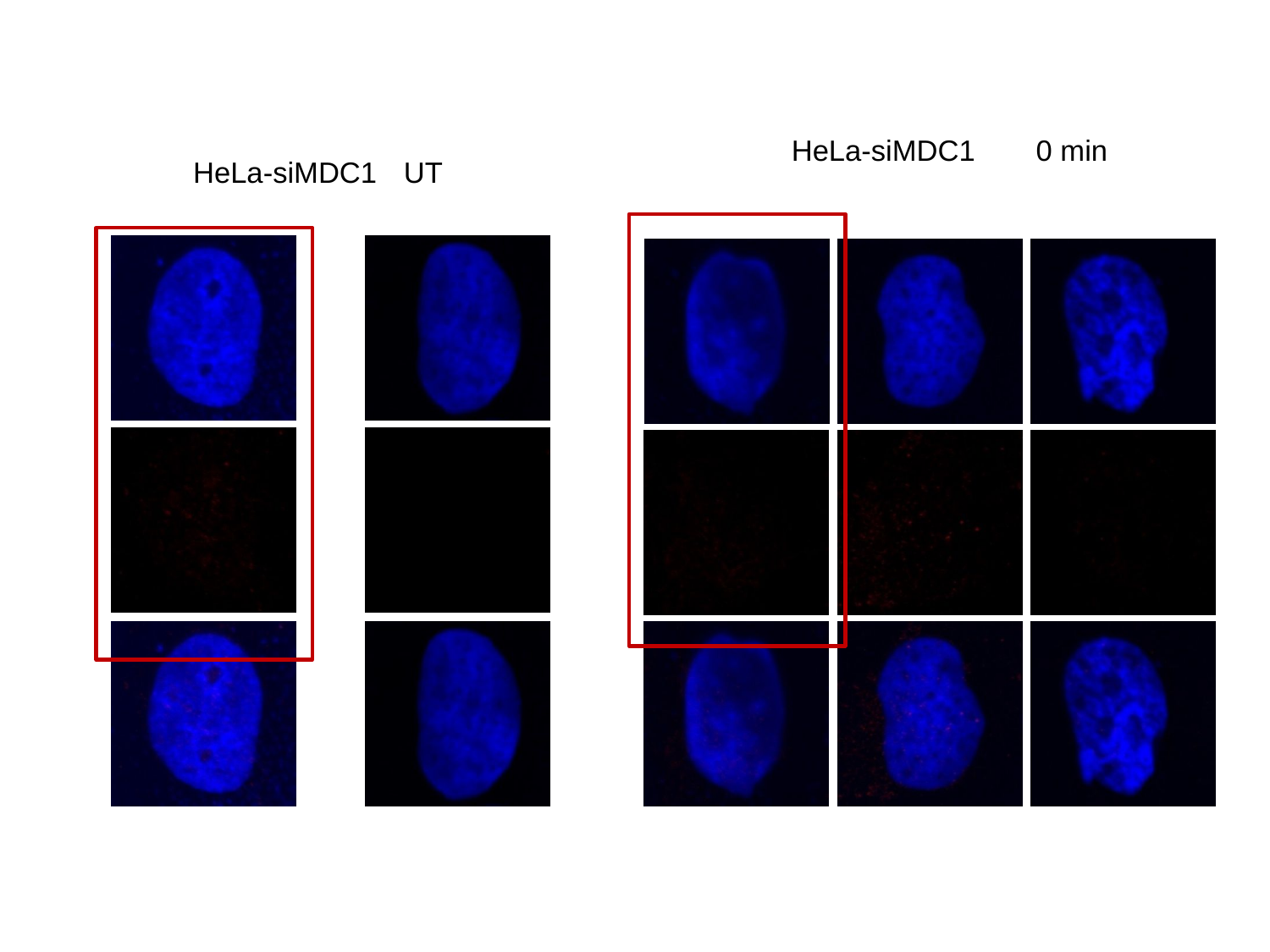

HeLa-siMDC1
0 min
HeLa-siMDC1
UT

## Slide 27
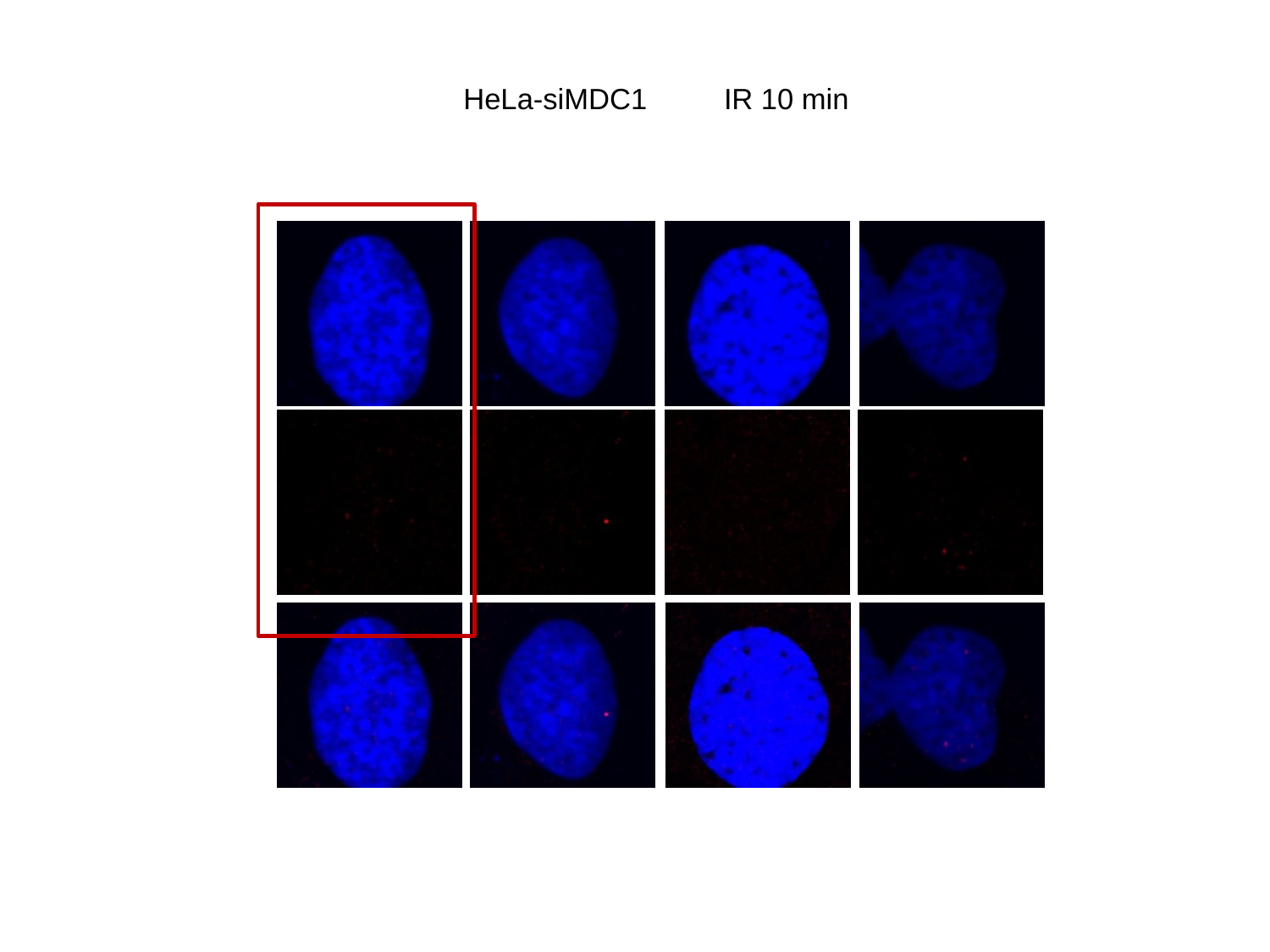

HeLa-siMDC1
IR 10 min

## Slide 28
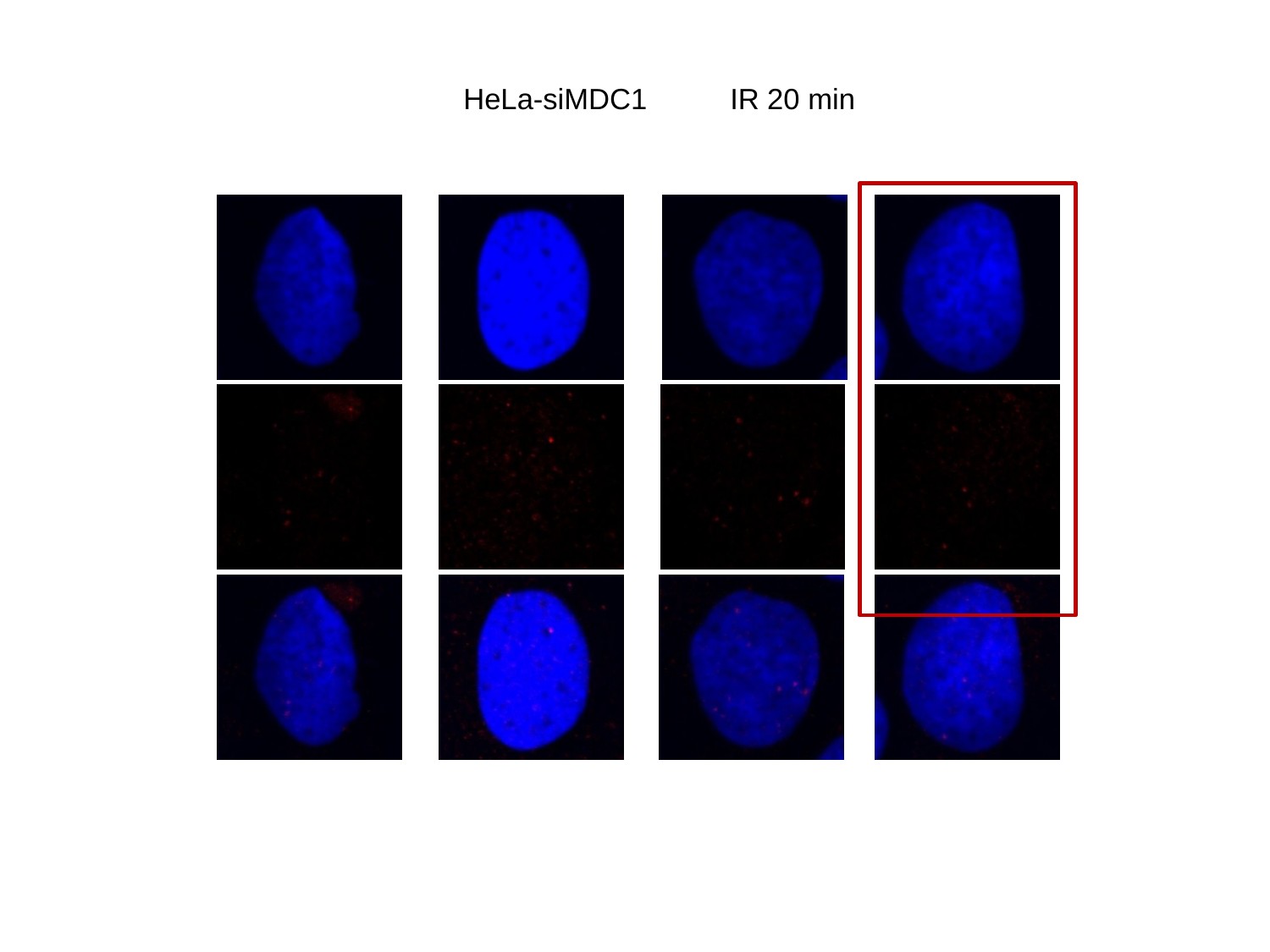

HeLa-siMDC1
IR 20 min

## Slide 29
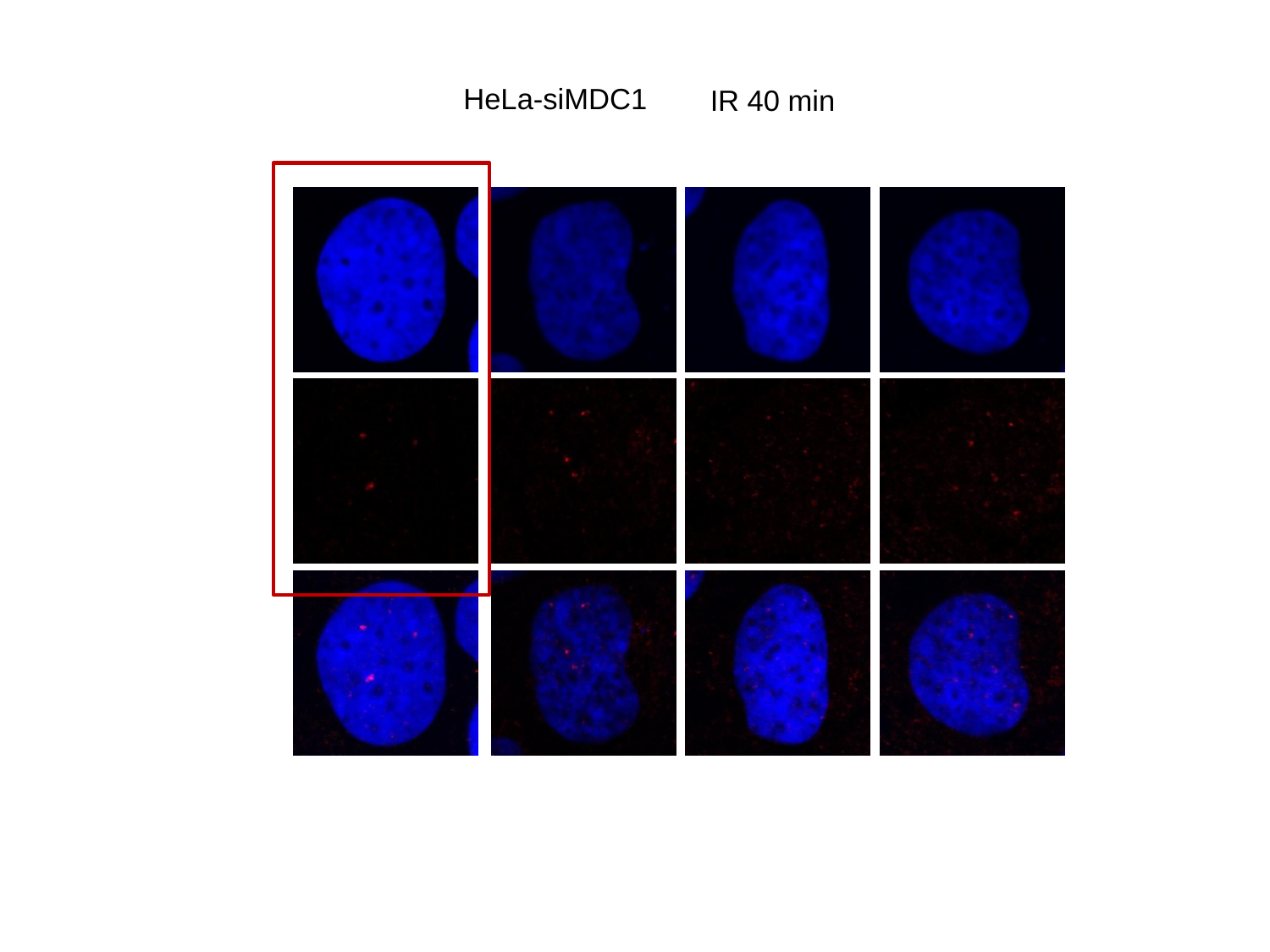

HeLa-siMDC1
IR 40 min

## Slide 30
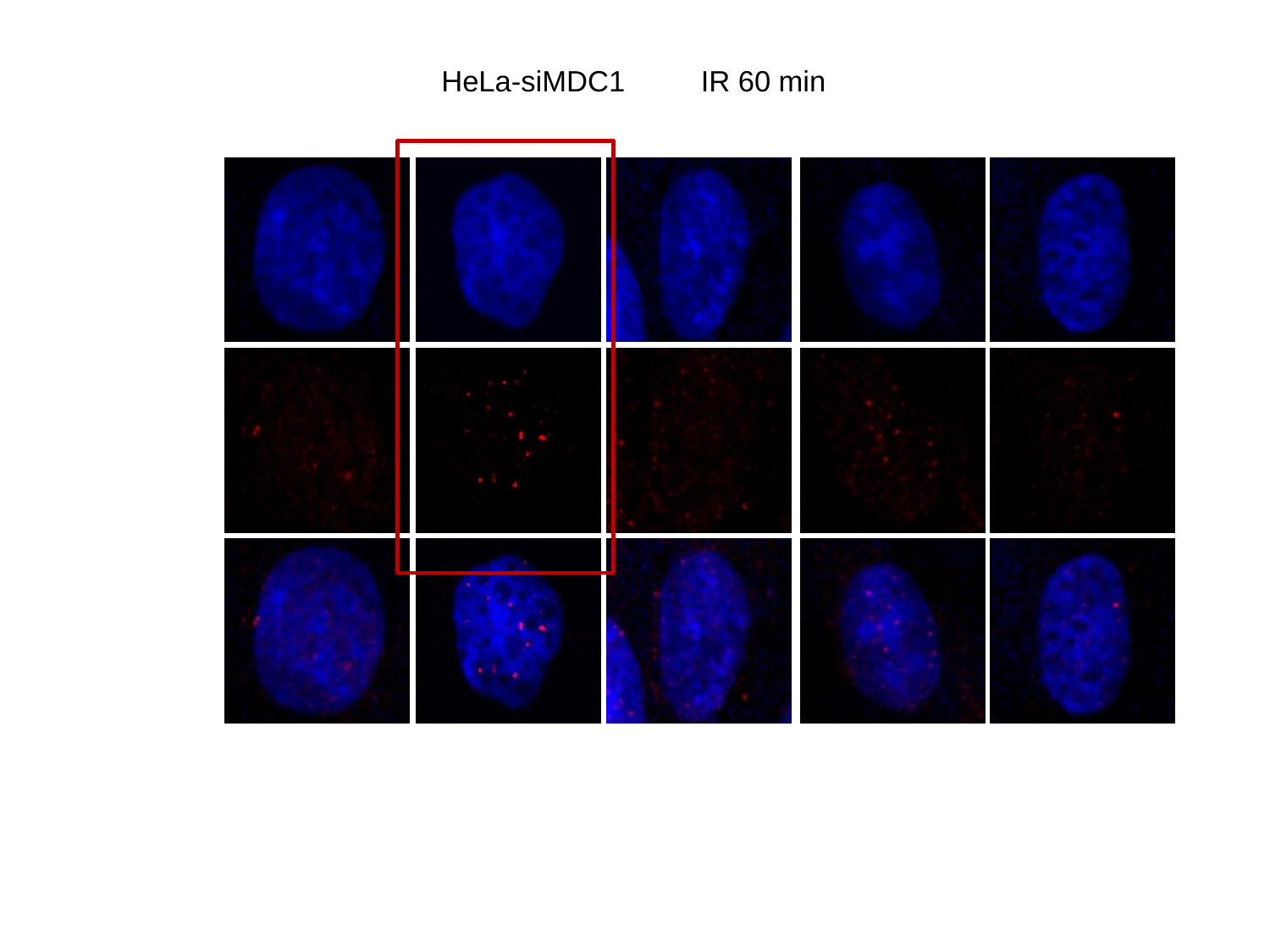

HeLa-siMDC1
IR 60 min

## Slide 31
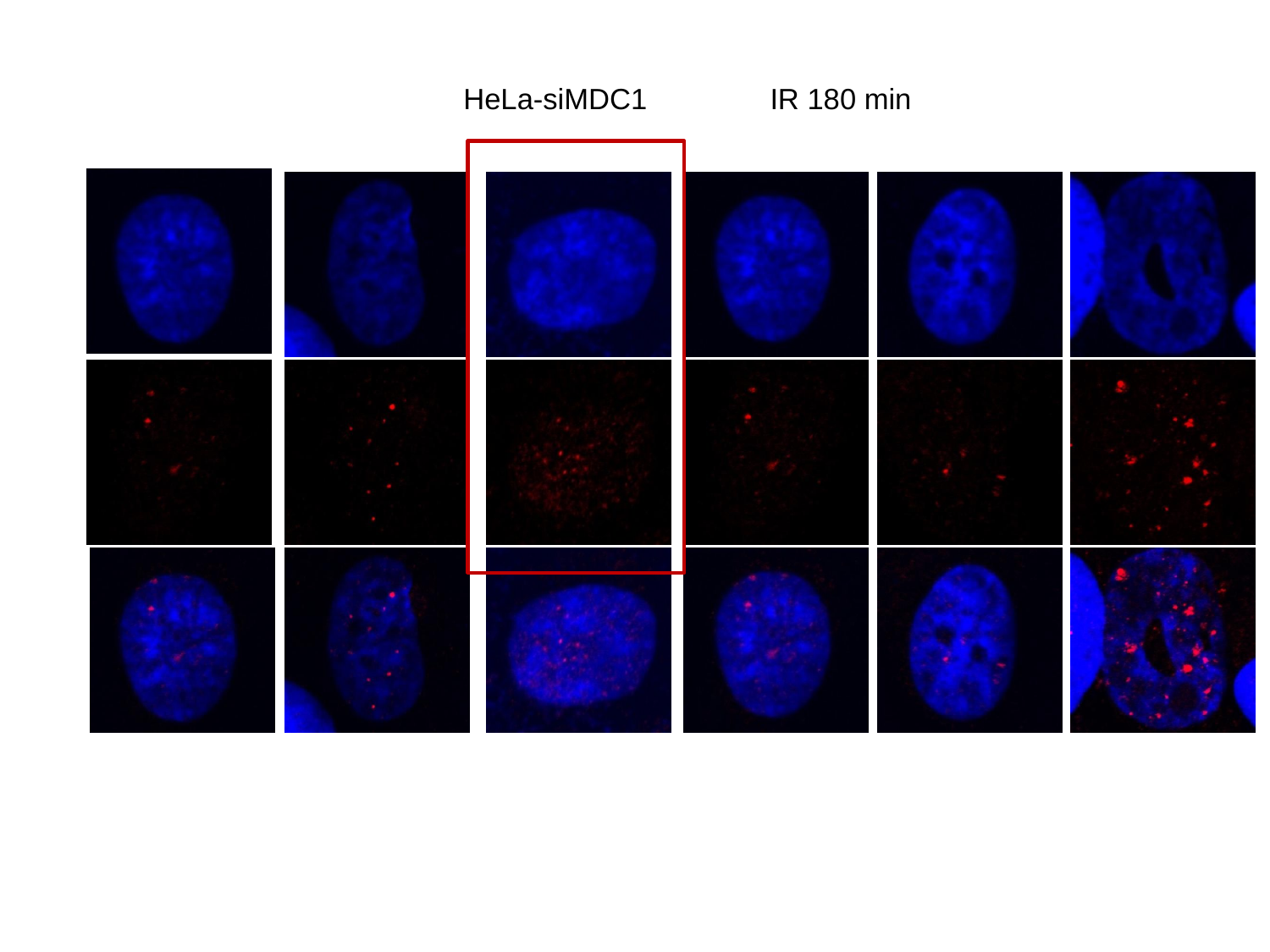

IR 180 min
HeLa-siMDC1
