# Supplementary material for: Author Correction: ID3 regulates the MDC1-mediated DNA damage response in order to maintain genome stability
Source: Nat Commun. 2018 Jun 6;9:2284. doi: 10.1038/s41467-018-04599-6 (PMC5989224; doi:10.1038/s41467-018-04599-6)
Supplement: Supplementary file 3 — Supplementary Data 3 [file 41467_2018_4599_MOESM3_ESM.zip › Fig7b/Fig 7b.pptx]

## Slide 1
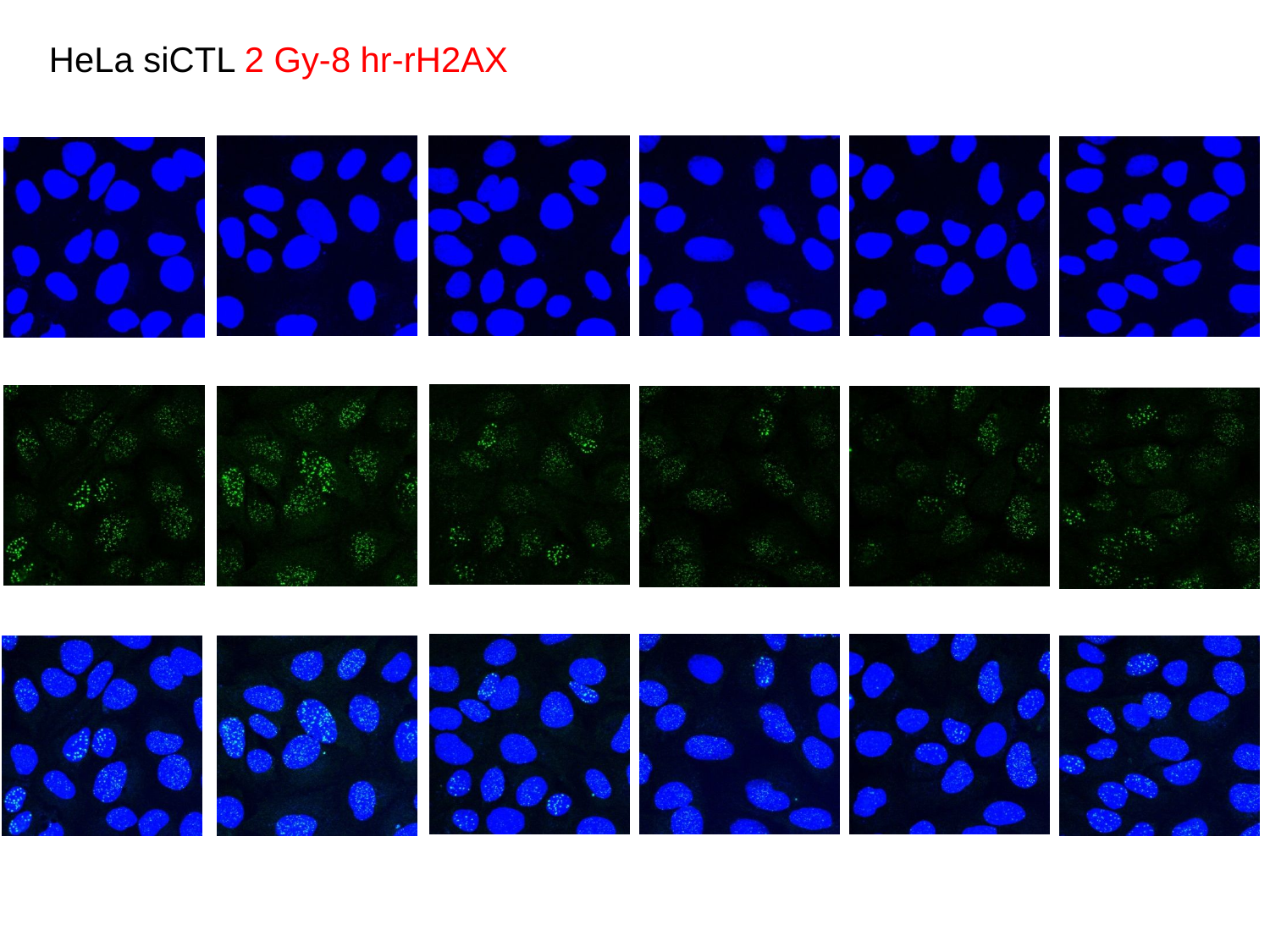

HeLa siCTL 2 Gy-8 hr-rH2AX

## Slide 2
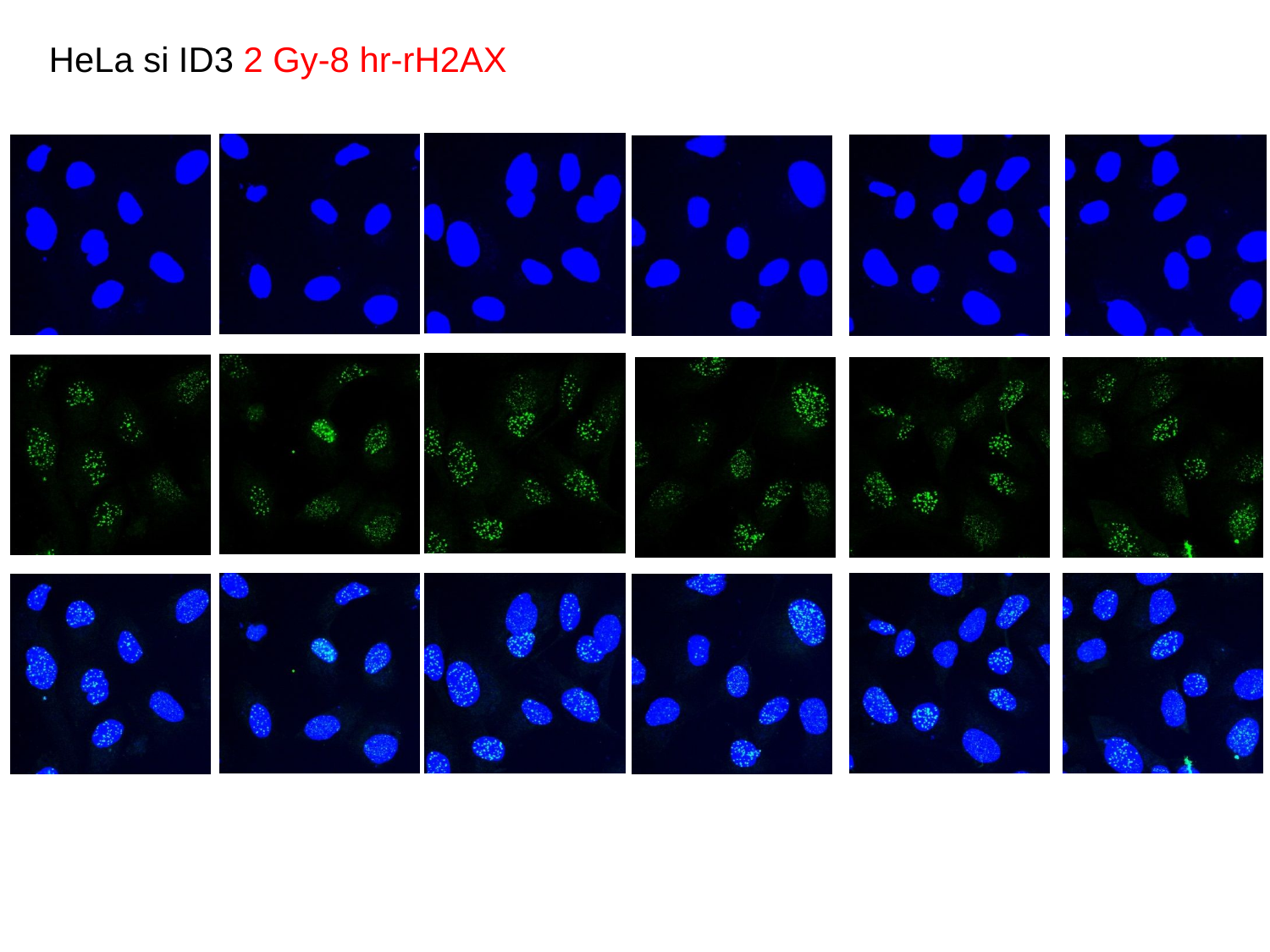

HeLa si ID3 2 Gy-8 hr-rH2AX

## Slide 3
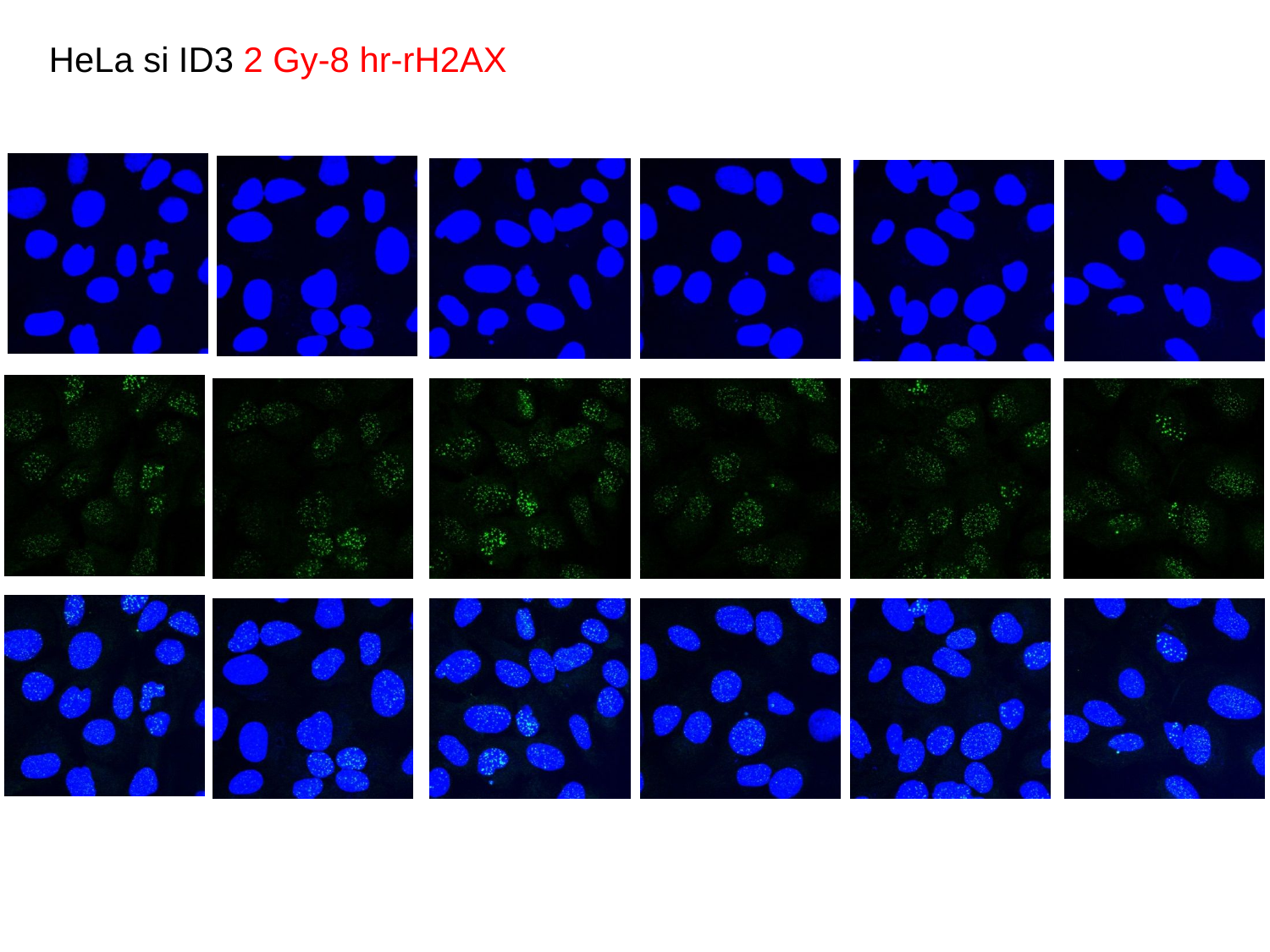

HeLa si ID3 2 Gy-8 hr-rH2AX

## Slide 4
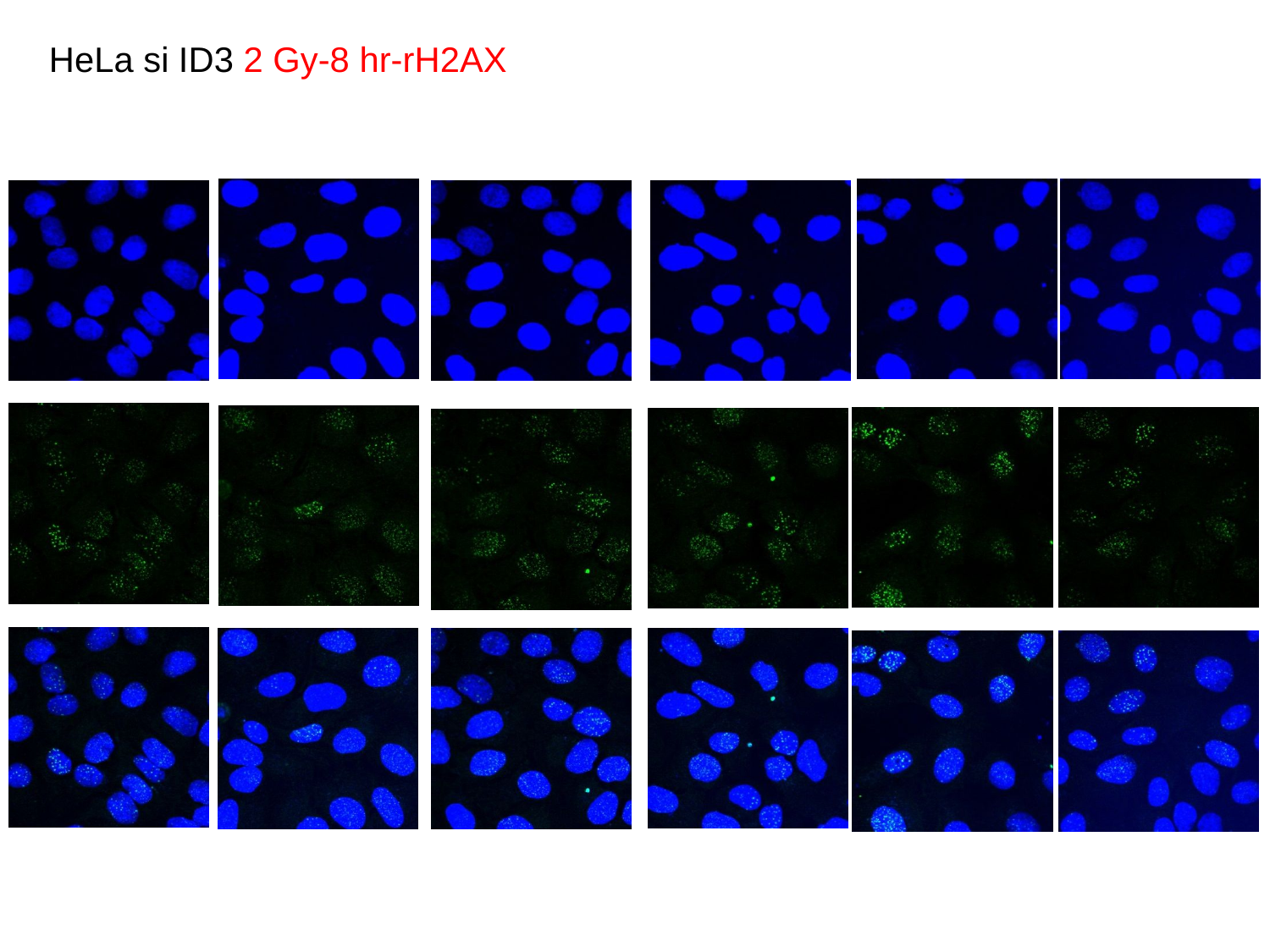

HeLa si ID3 2 Gy-8 hr-rH2AX

## Slide 5
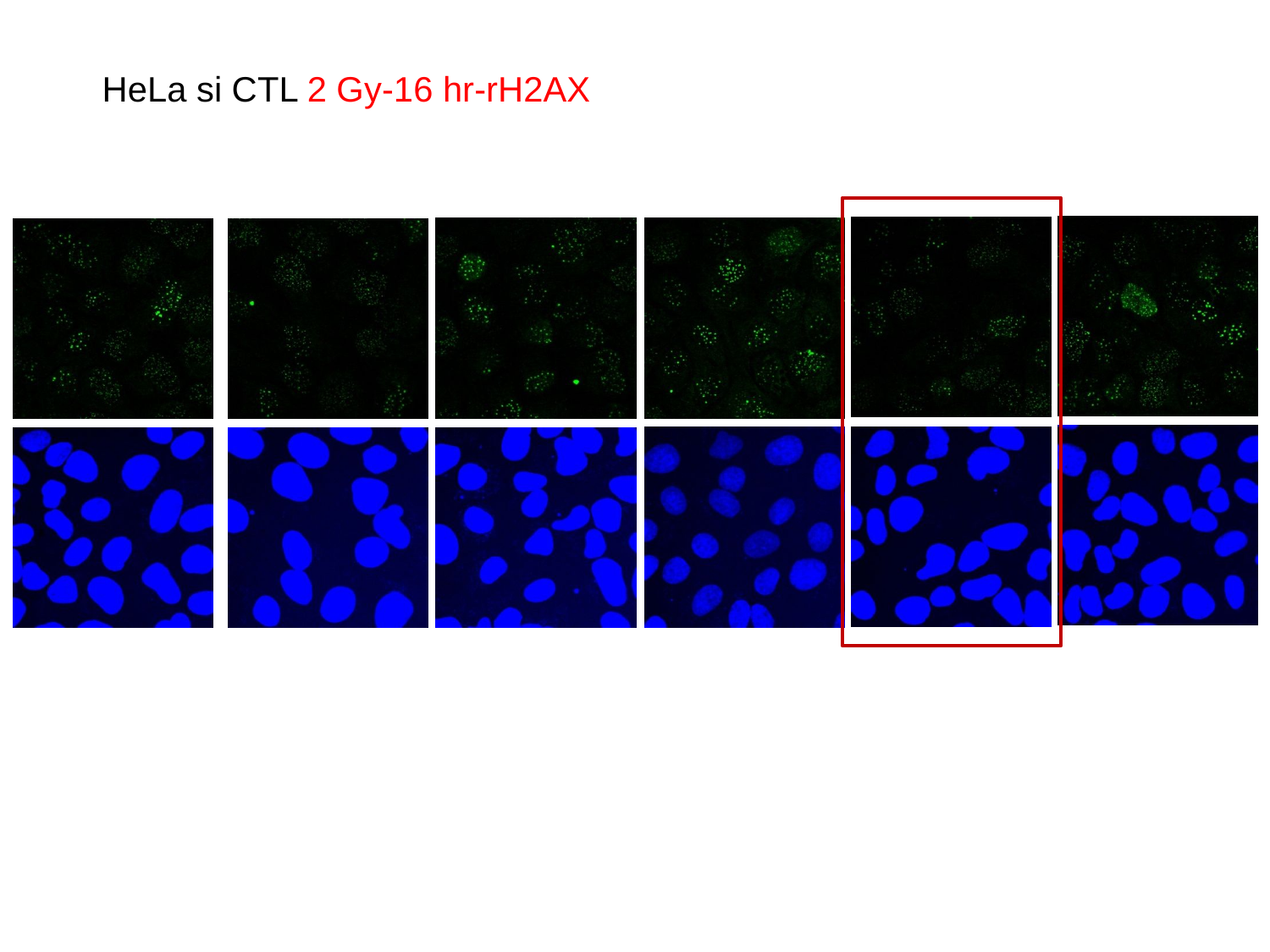

HeLa si CTL 2 Gy-16 hr-rH2AX

## Slide 6
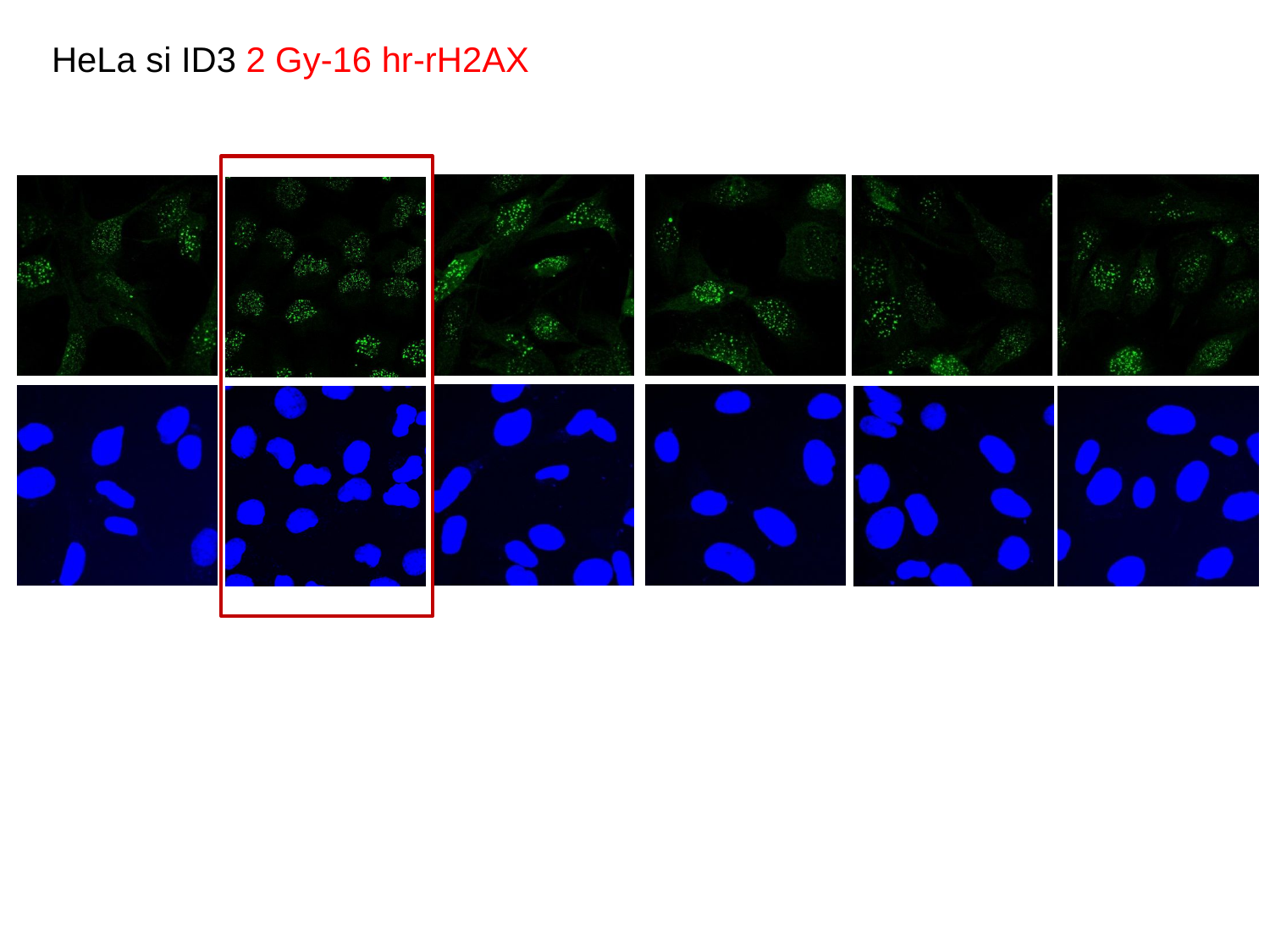

HeLa si ID3 2 Gy-16 hr-rH2AX

## Slide 7
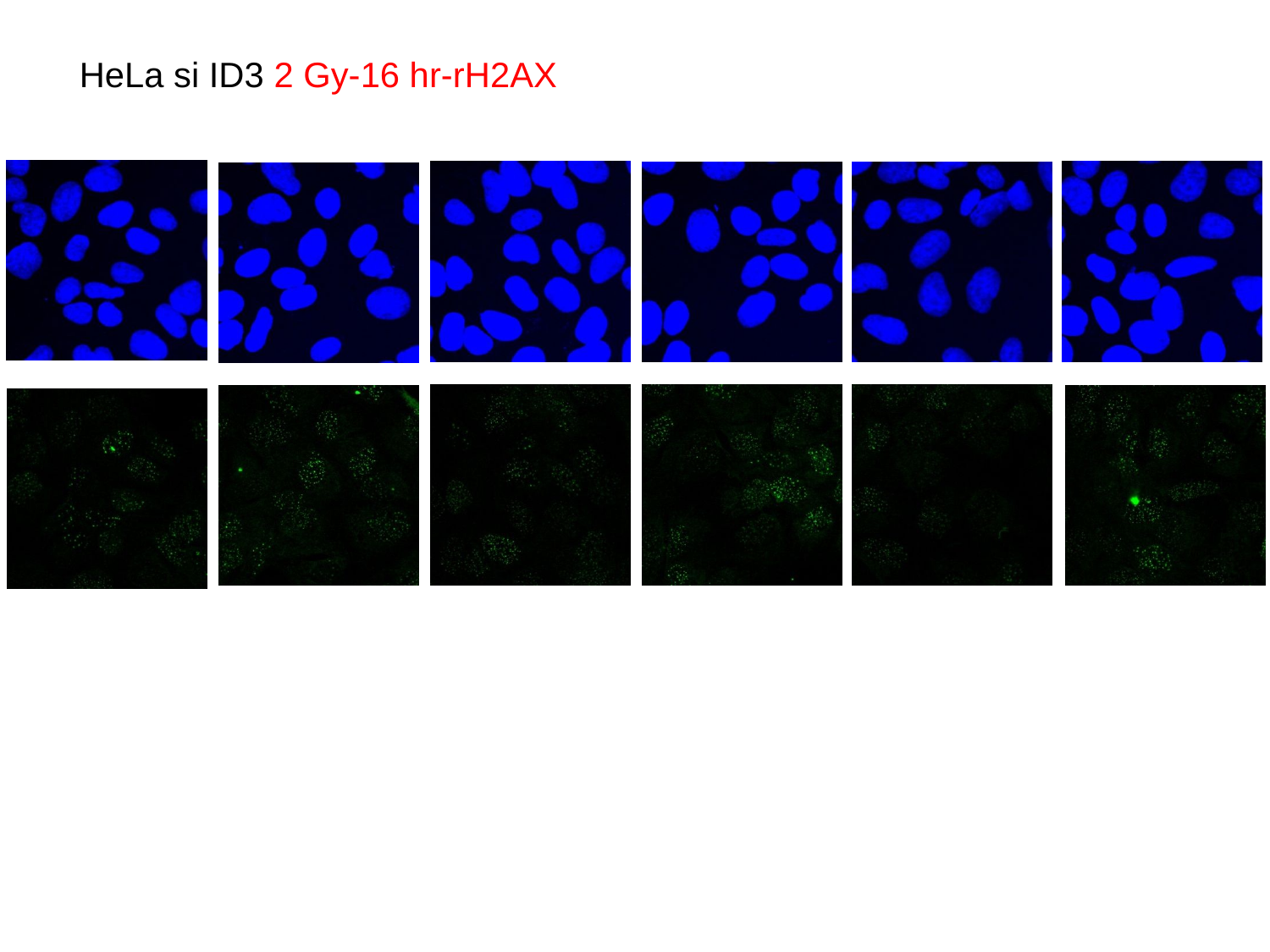

HeLa si ID3 2 Gy-16 hr-rH2AX

## Slide 8
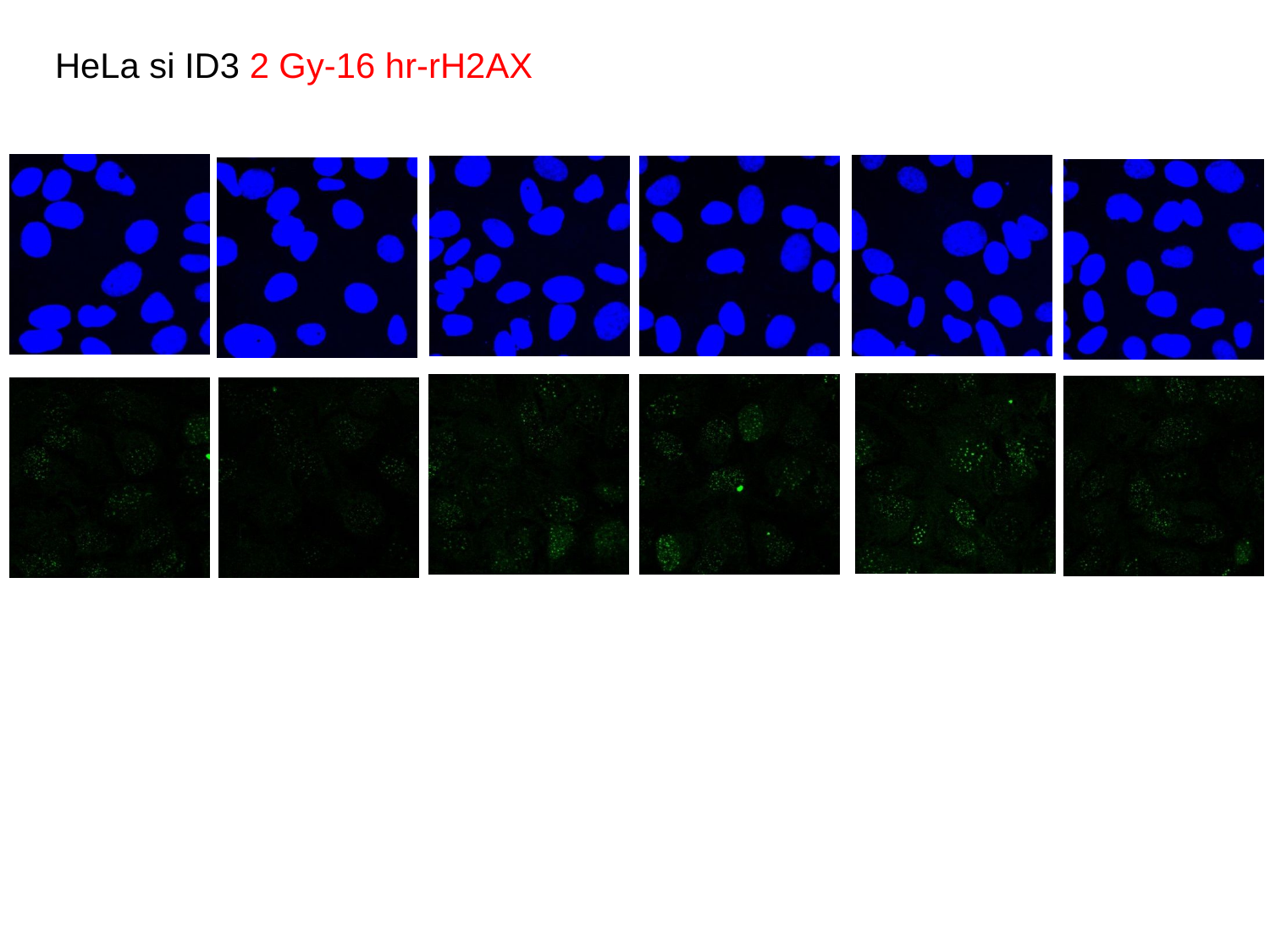

HeLa si ID3 2 Gy-16 hr-rH2AX

## Slide 9
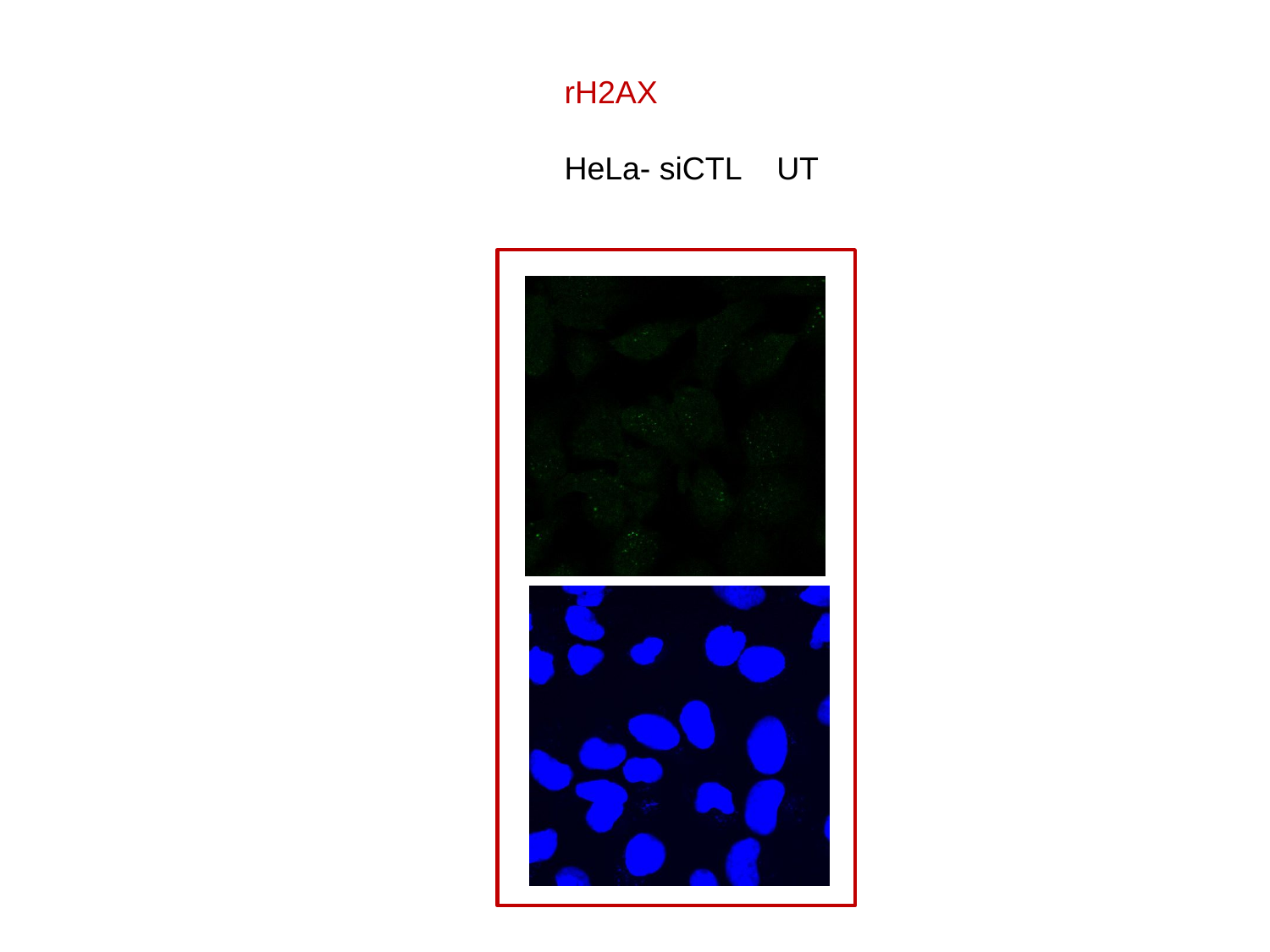

rH2AX
HeLa- siCTL UT

## Slide 10
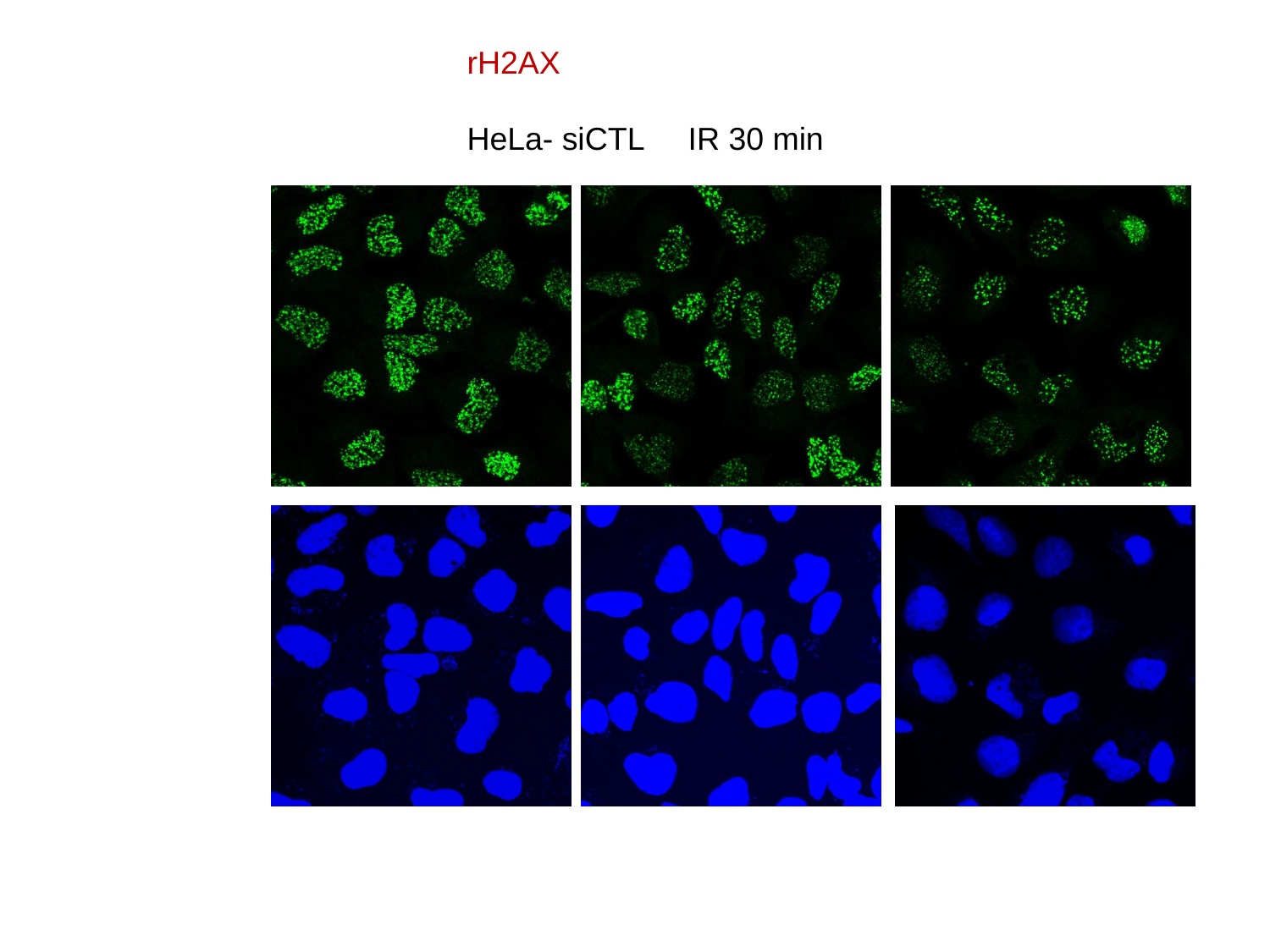

rH2AX
HeLa- siCTL IR 30 min

## Slide 11
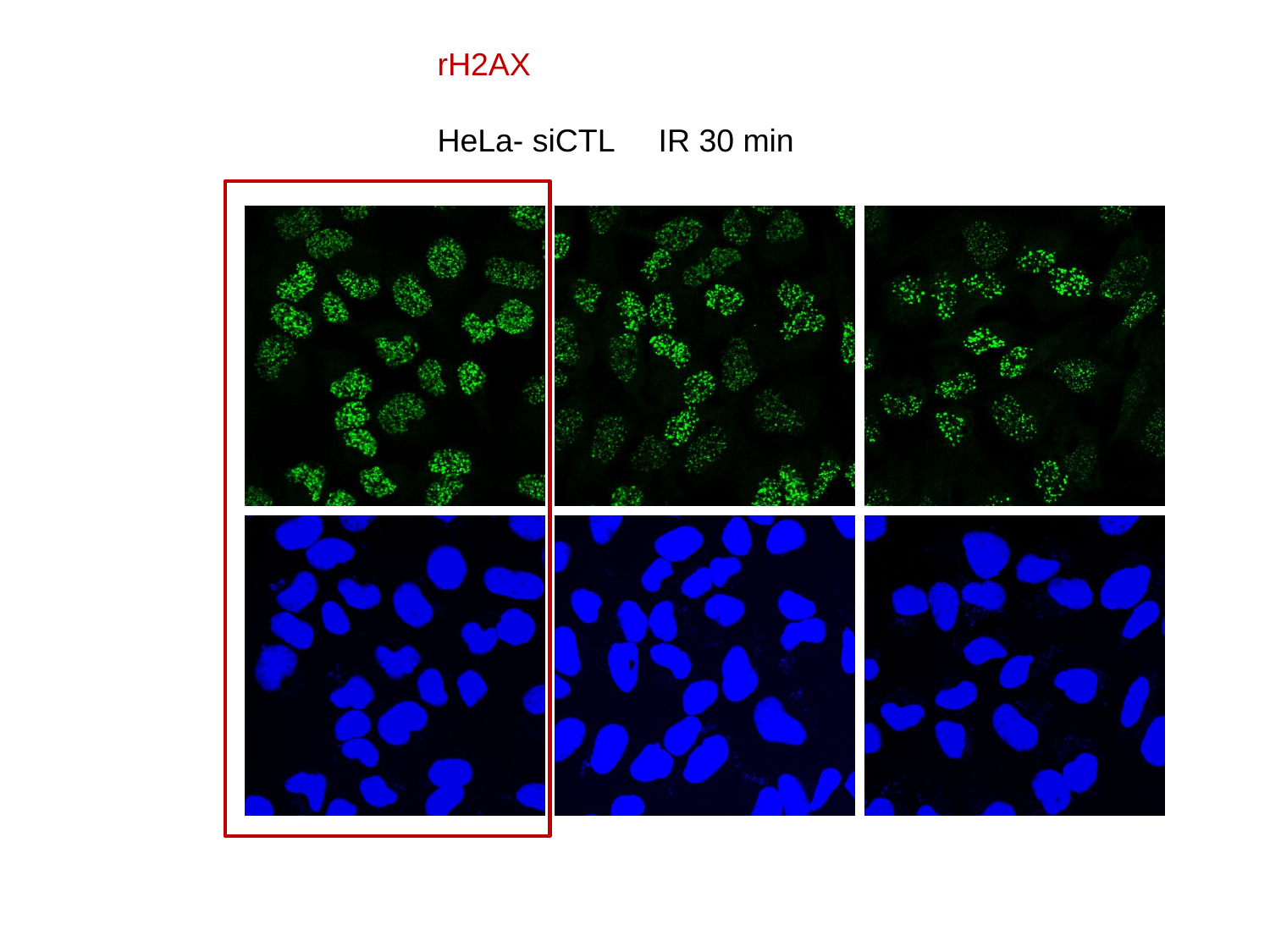

rH2AX
HeLa- siCTL IR 30 min

## Slide 12
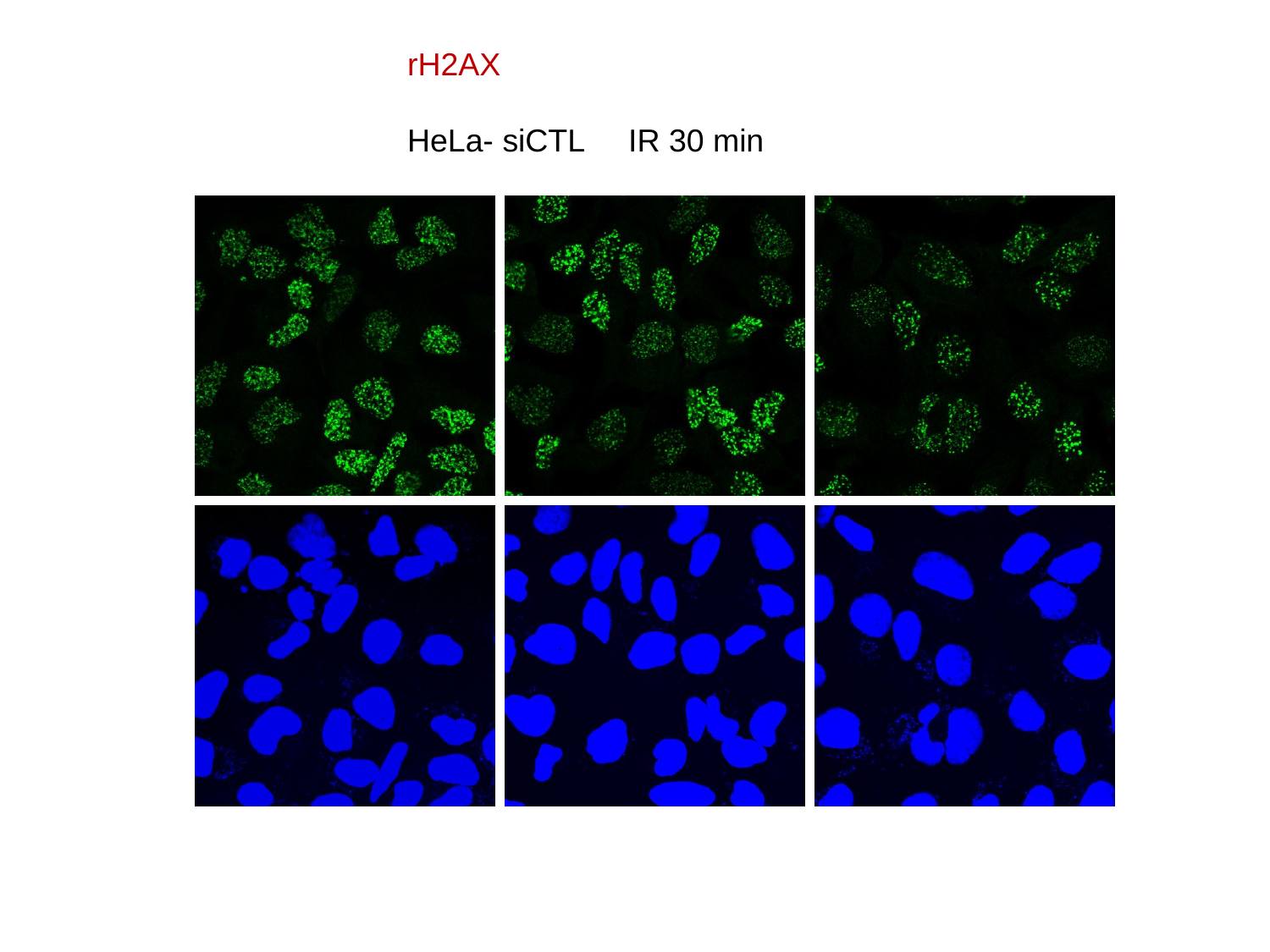

rH2AX
HeLa- siCTL IR 30 min

## Slide 13
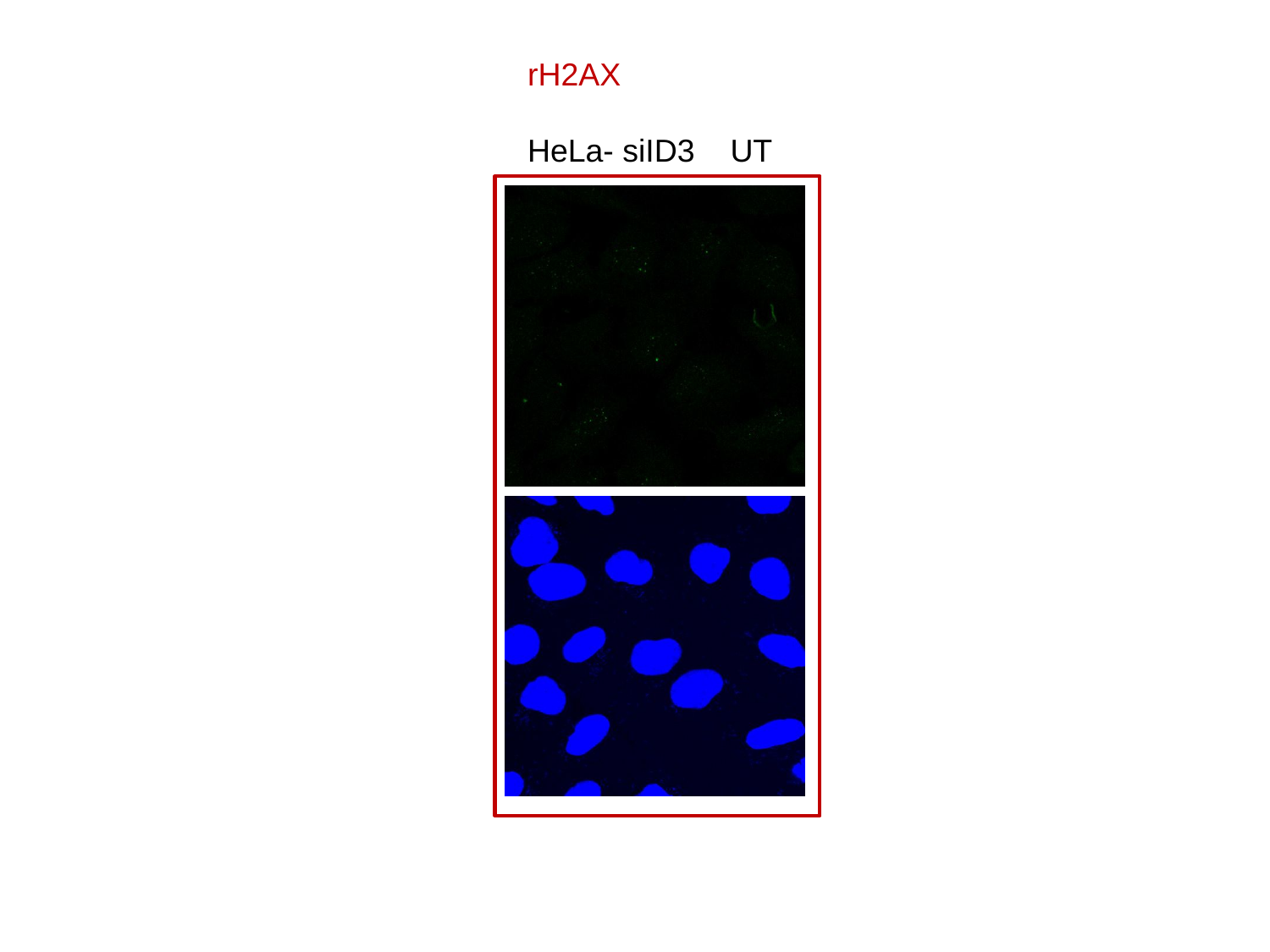

rH2AX
HeLa- siID3 UT

## Slide 14
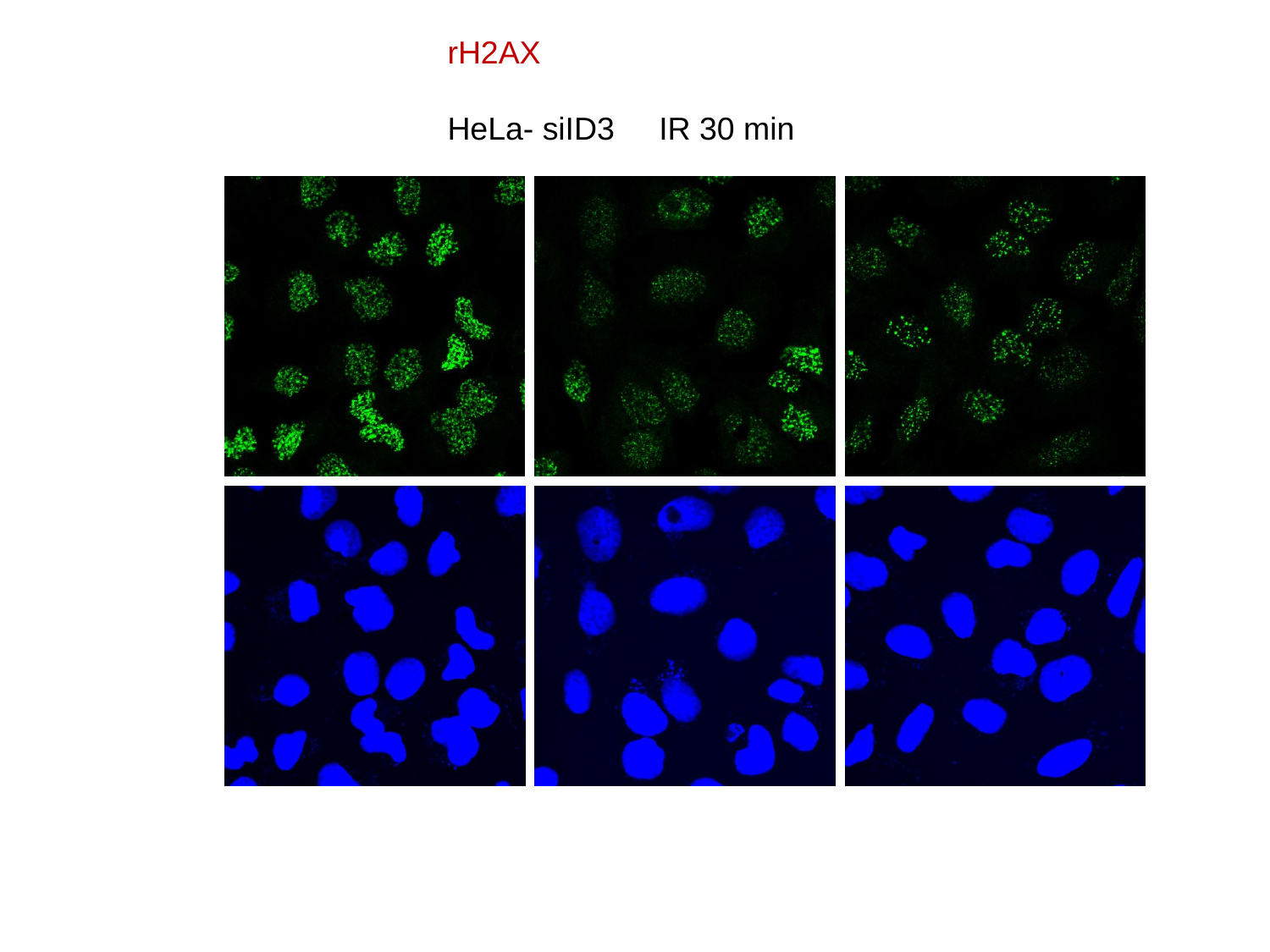

rH2AX
HeLa- siID3 IR 30 min

## Slide 15
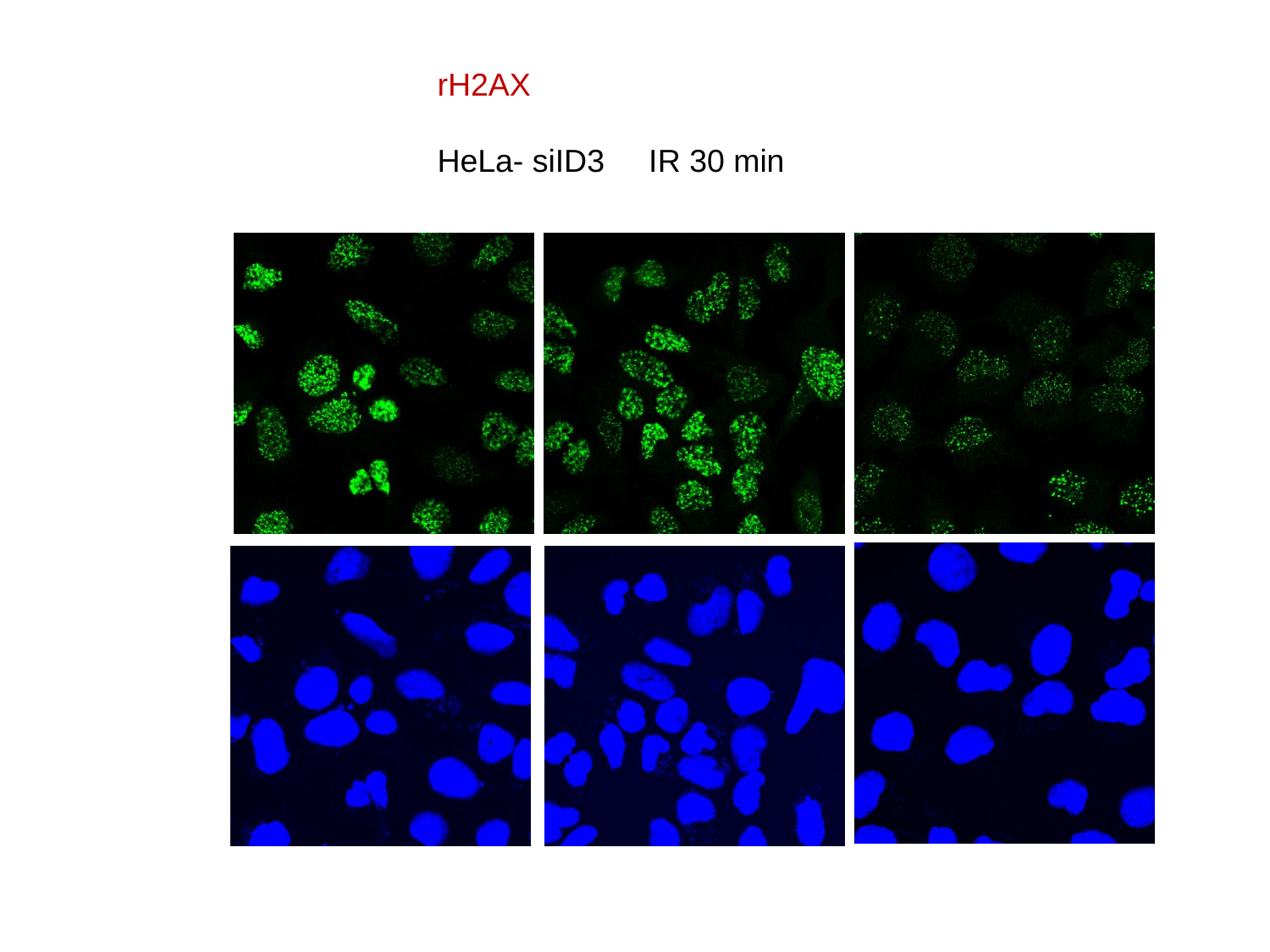

rH2AX
HeLa- siID3 IR 30 min

## Slide 16
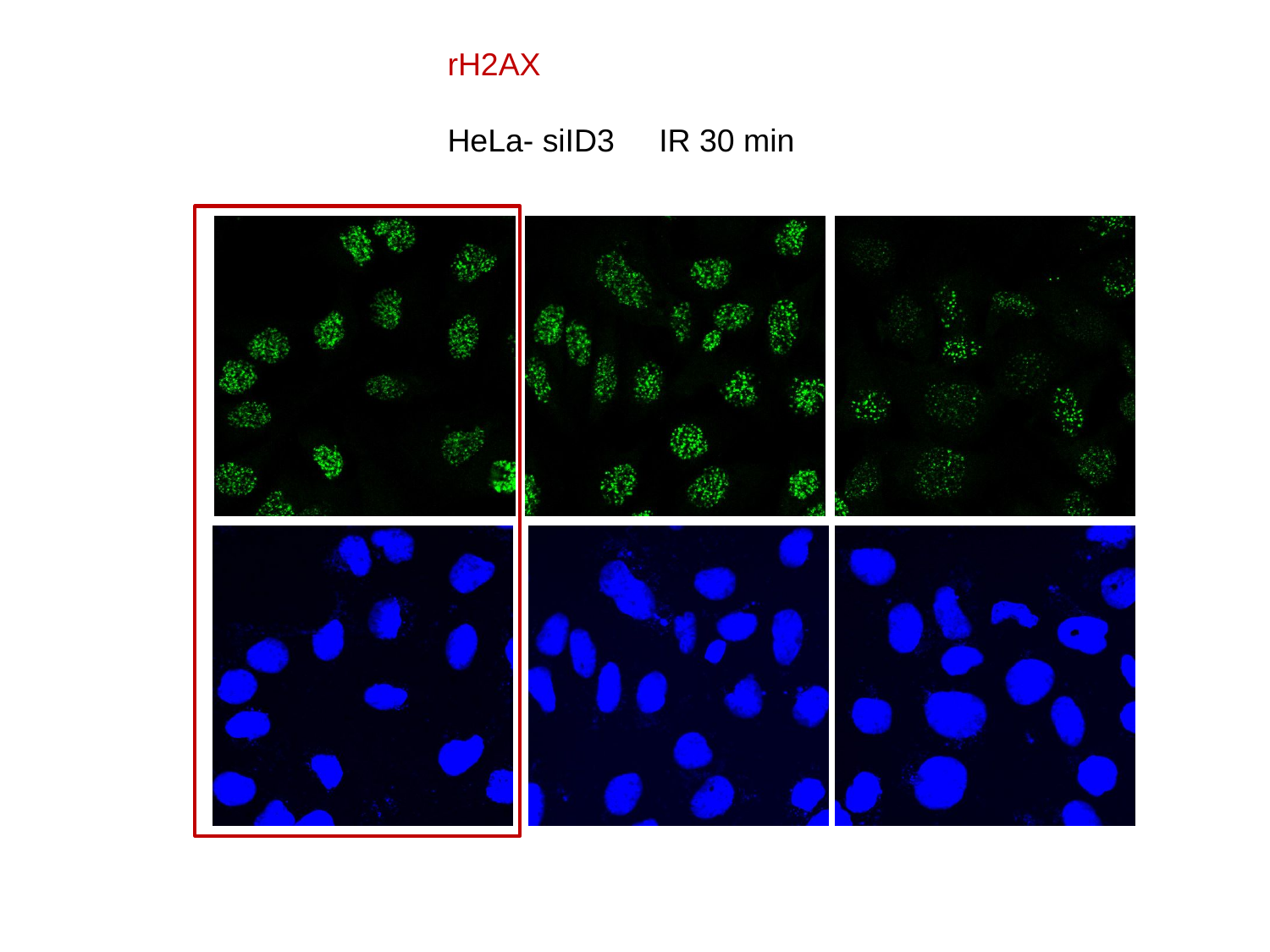

rH2AX
HeLa- siID3 IR 30 min
