# Supplementary material for: Author Correction: ID3 regulates the MDC1-mediated DNA damage response in order to maintain genome stability
Source: Nat Commun. 2018 Jun 6;9:2284. doi: 10.1038/s41467-018-04599-6 (PMC5989224; doi:10.1038/s41467-018-04599-6)
Supplement: Supplementary file 3 — Supplementary Data 3 [file 41467_2018_4599_MOESM3_ESM.zip › fig7gi/Fig 7i.pptx]

## Slide 1
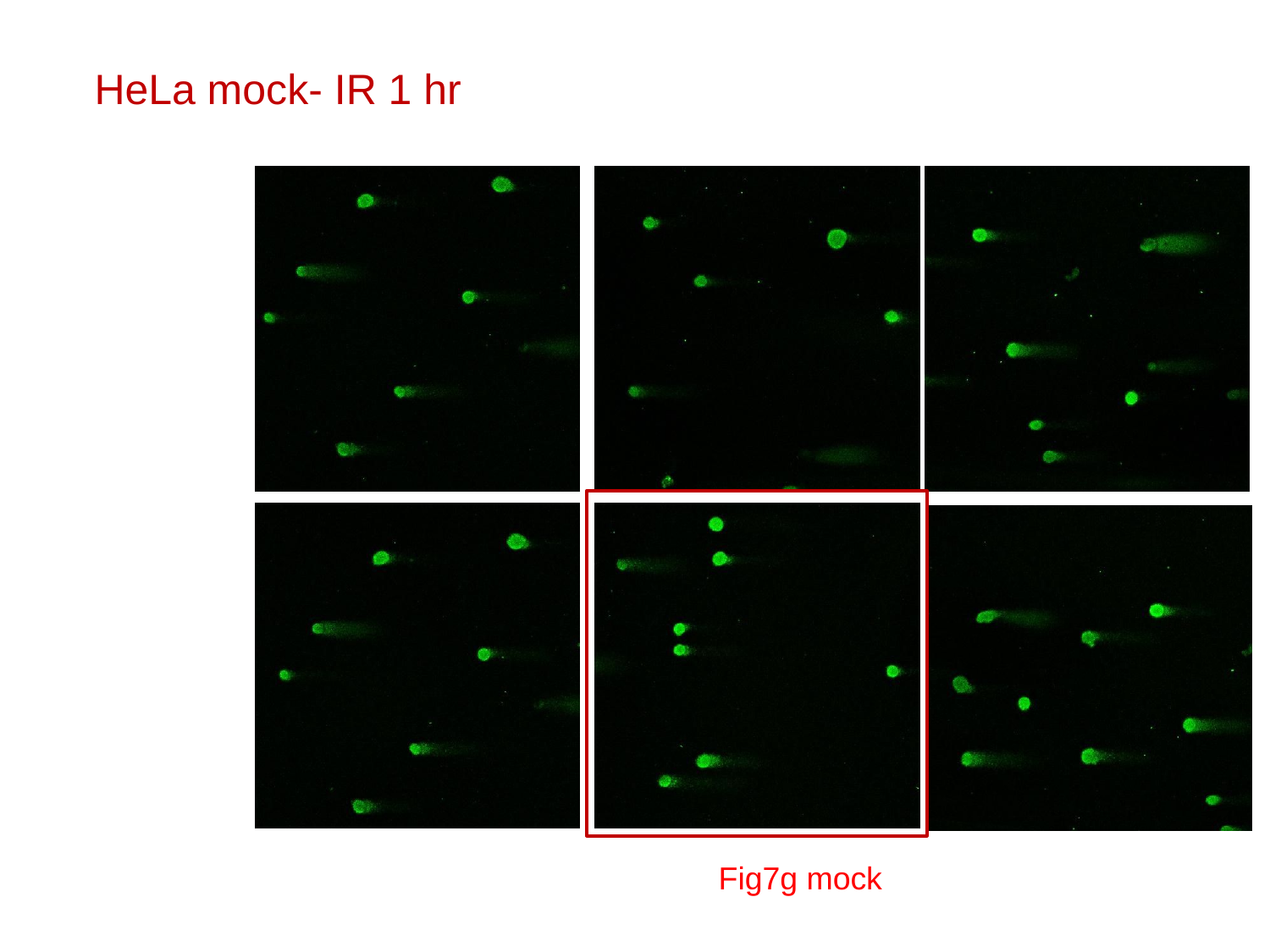

HeLa mock- IR 1 hr
Fig7g mock

## Slide 2
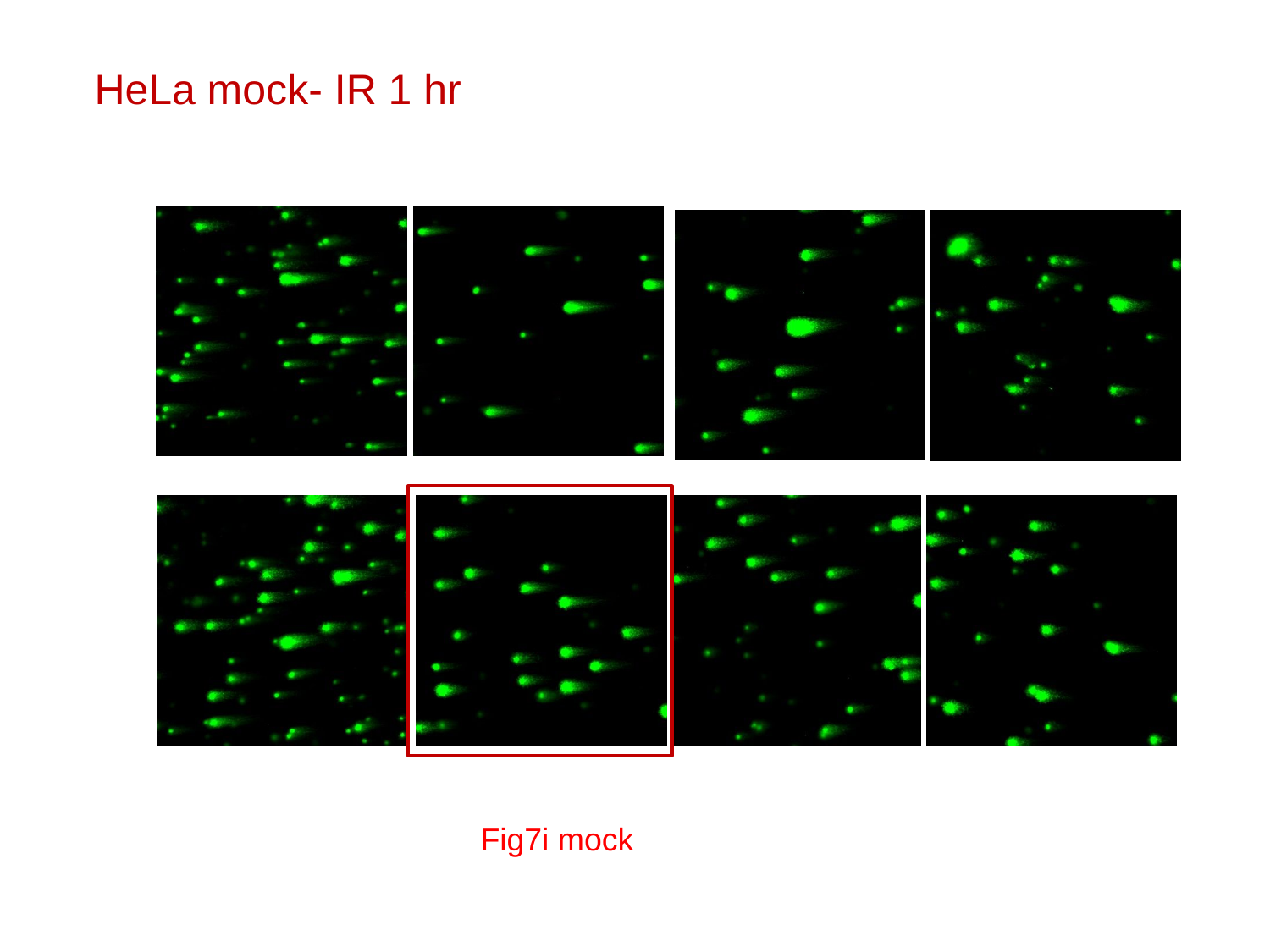

HeLa mock- IR 1 hr
Fig7i mock

## Slide 3
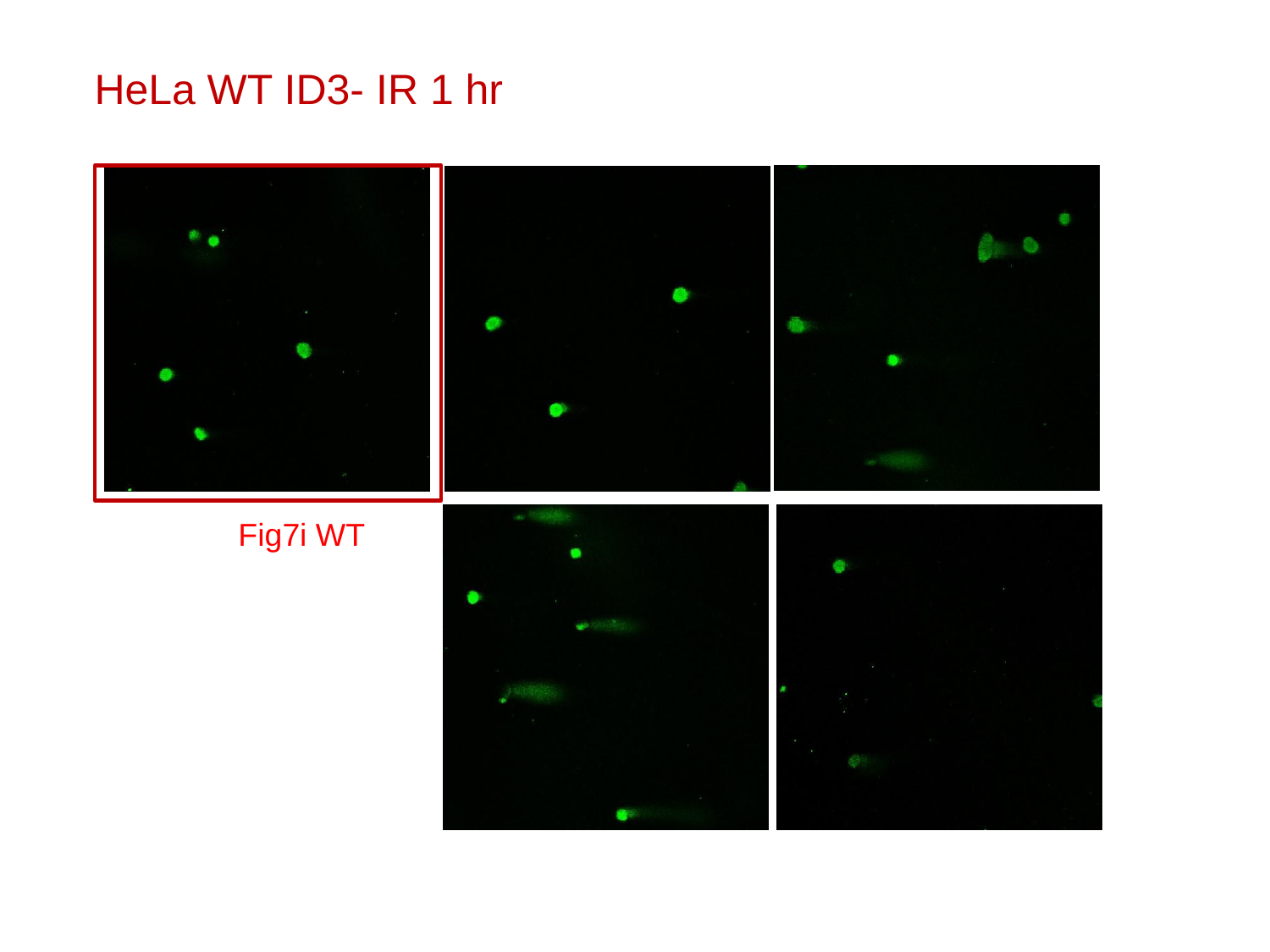

HeLa WT ID3- IR 1 hr
Fig7i WT

## Slide 4
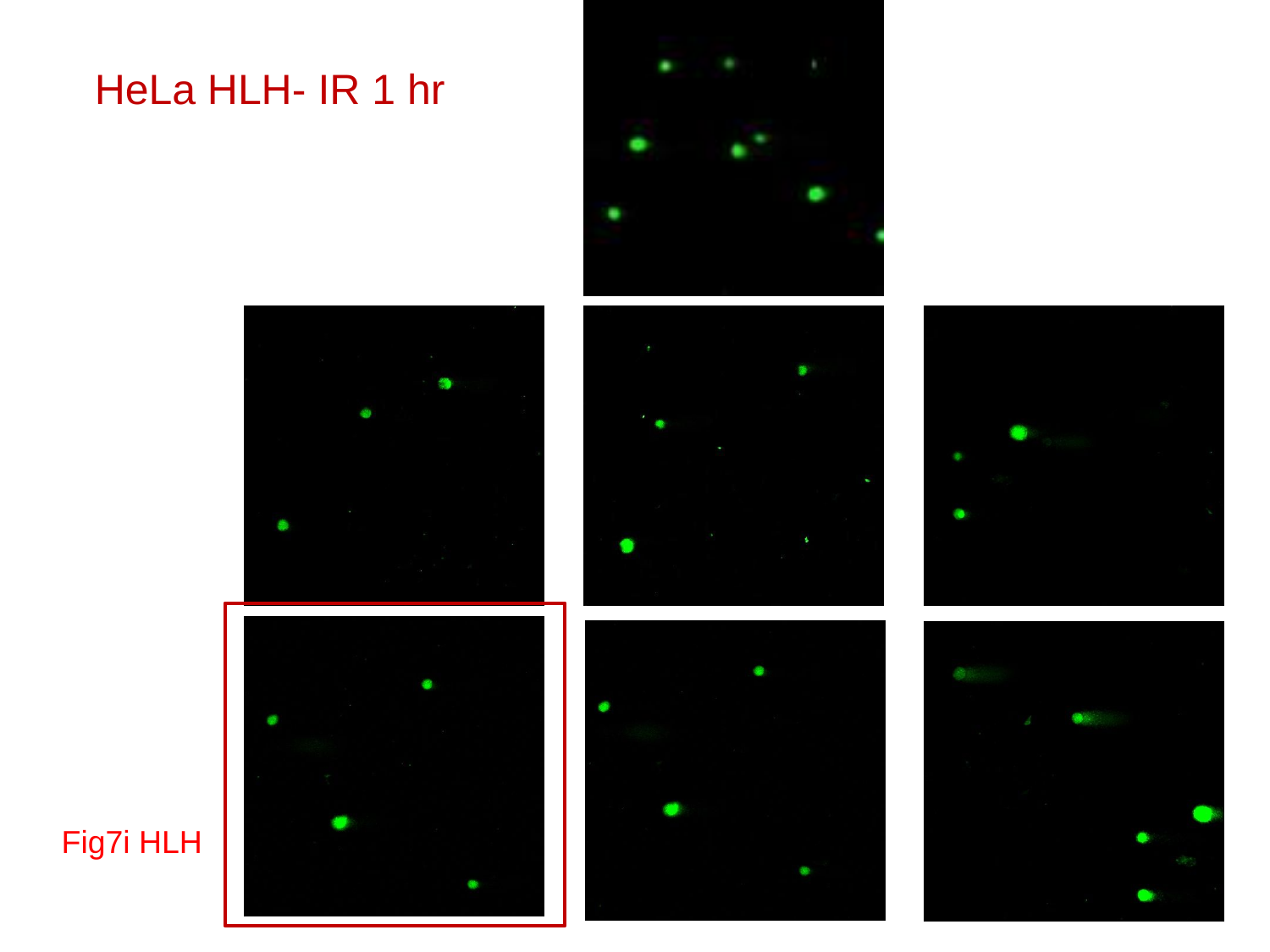

HeLa HLH- IR 1 hr
Fig7i HLH

## Slide 5
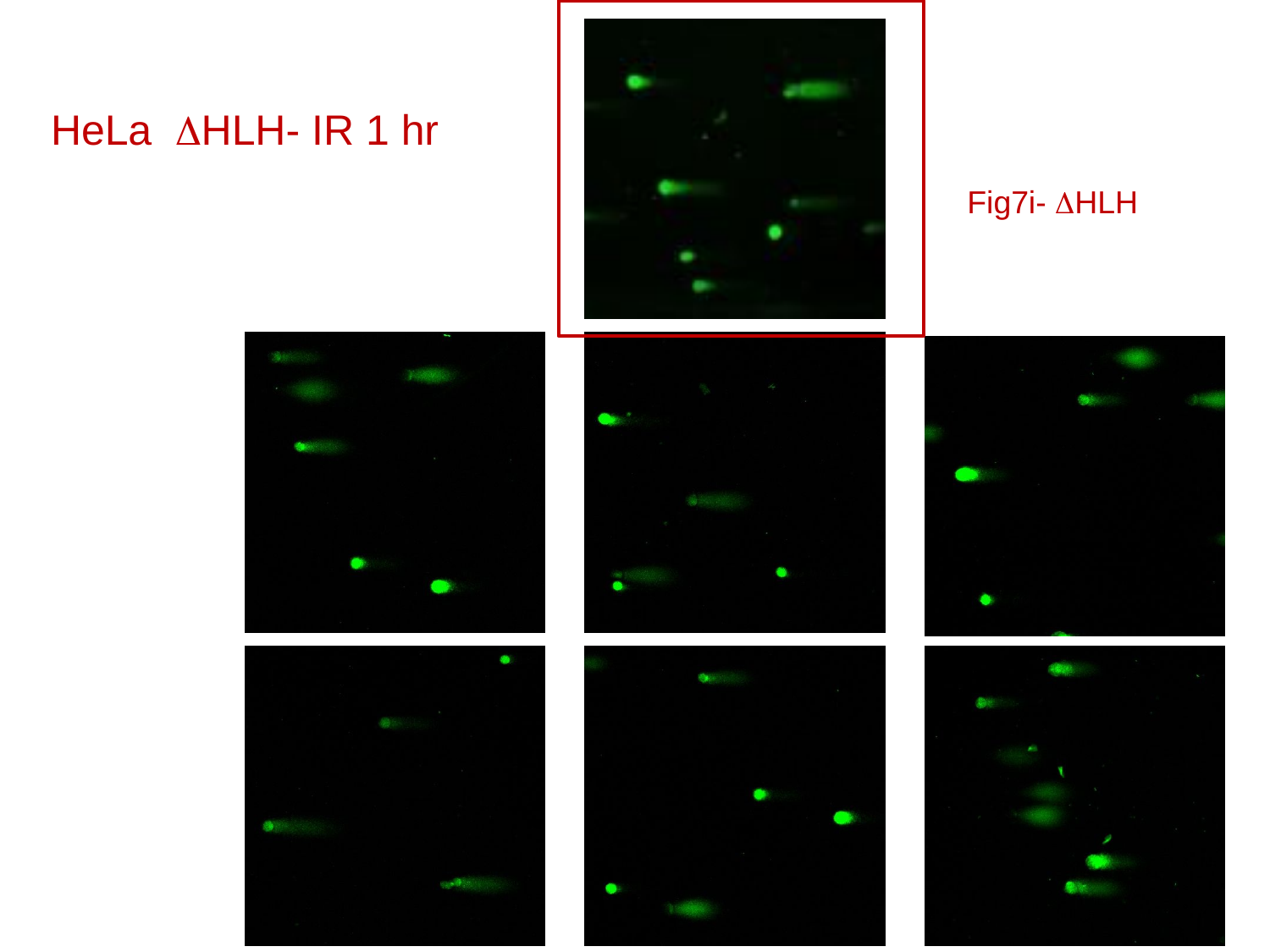

HeLa ΔHLH- IR 1 hr
Fig7i- ΔHLH
